# From Neandertals to modern humans: New data on the Uluzzian

**DOI:** 10.1371/journal.pone.0196786

**Published:** 2018-05-09

**Authors:** Paola Villa, Luca Pollarolo, Jacopo Conforti, Fabrizio Marra, Cristian Biagioni, Ilaria Degano, Jeannette J. Lucejko, Carlo Tozzi, Massimo Pennacchioni, Giovanni Zanchetta, Cristiano Nicosia, Marco Martini, Emanuela Sibilia, Laura Panzeri

**Affiliations:** 1 University of Colorado Museum, Boulder, Colorado, United States of America; 2 Istituto Italiano di Paleontologia Umana, Rome, Italy; 3 School of Geography, Archaeology and Environmental Studies, University of the Witwatersrand, Johannesburg, South Africa; 4 Laboratoire Archéologie et Peuplement de l’Afrique, University of Geneva, Geneva, Switzerland; 5 Dipartimento Civiltá e Forme del Sapere, Università di Pisa, Pisa, Italy; 6 Istituto Nazionale di Geofisica e Vulcanologia, Roma, Italy; 7 Dipartimento di Scienze della Terra, Università di Pisa, Pisa, Italy; 8 Department of Chemistry and Industrial Chemistry, University of Pisa,Pisa, Italy; 9 Dipartimento di Studi Umanistici, Università di Roma 3, Rome, Italy; 10 Centro Informazioni Geotopografiche Aeronautiche, Italian Military Air Force, Pomezia, Rome, Italy; 11 Dipartimento dei Beni Culturali, Università di Padova, Padova, Italy; 12 Dipartimento di Scienza dei Materiali, Università di Milano-Bicocca, Milano, Italy; University of Oxford, UNITED KINGDOM

## Abstract

Having thrived in Eurasia for 350,000 years Neandertals disappeared from the record around 40,000–37,000 years ago, after modern humans entered Europe. It was a complex process of population interactions that included cultural exchanges and admixture between Neandertals and dispersing groups of modern humans. In Europe Neandertals are always associated with the Mousterian while the Aurignacian is associated with modern humans only. The onset of the Aurignacian is preceded by “transitional” industries which show some similarities with the Mousterian but also contain modern tool forms. Information on these industries is often incomplete or disputed and this is true of the Uluzzian. We present the results of taphonomic, typological and technological analyses of two Uluzzian sites, Grotta La Fabbrica (Tuscany) and the newly discovered site of Colle Rotondo (Latium). Comparisons with Castelcivita and Grotta del Cavallo show that the Uluzzian is a coherent cultural unit lasting about five millennia, replaced by the Protoaurignacian before the eruption of the Campanian Ignimbrite. The lack of skeletal remains at our two sites and the controversy surrounding the stratigraphic position of modern human teeth at Cavallo makes it difficult to reach agreement about authorship of the Uluzzian, for which alternative hypotheses have been proposed. Pending the discovery of DNA or further human remains, these hypotheses can only be evaluated by archaeological arguments, i.e. evidence of continuities and discontinuities between the Uluzzian and the preceding and succeeding culture units in Italy. However, in the context of “transitional” industries with disputed dates for the arrival of modern humans in Europe, and considering the case of the Châtelperronian, an Upper Paleolithic industry made by Neandertals, typo-technology used as an indicator of hominin authorship has limited predictive value. We corroborate previous suggestions that the Middle-to-Upper Paleolithic transition occurred as steps of rapid changes and geographically uneven rates of spread.

## Introduction

**Fig 1:**
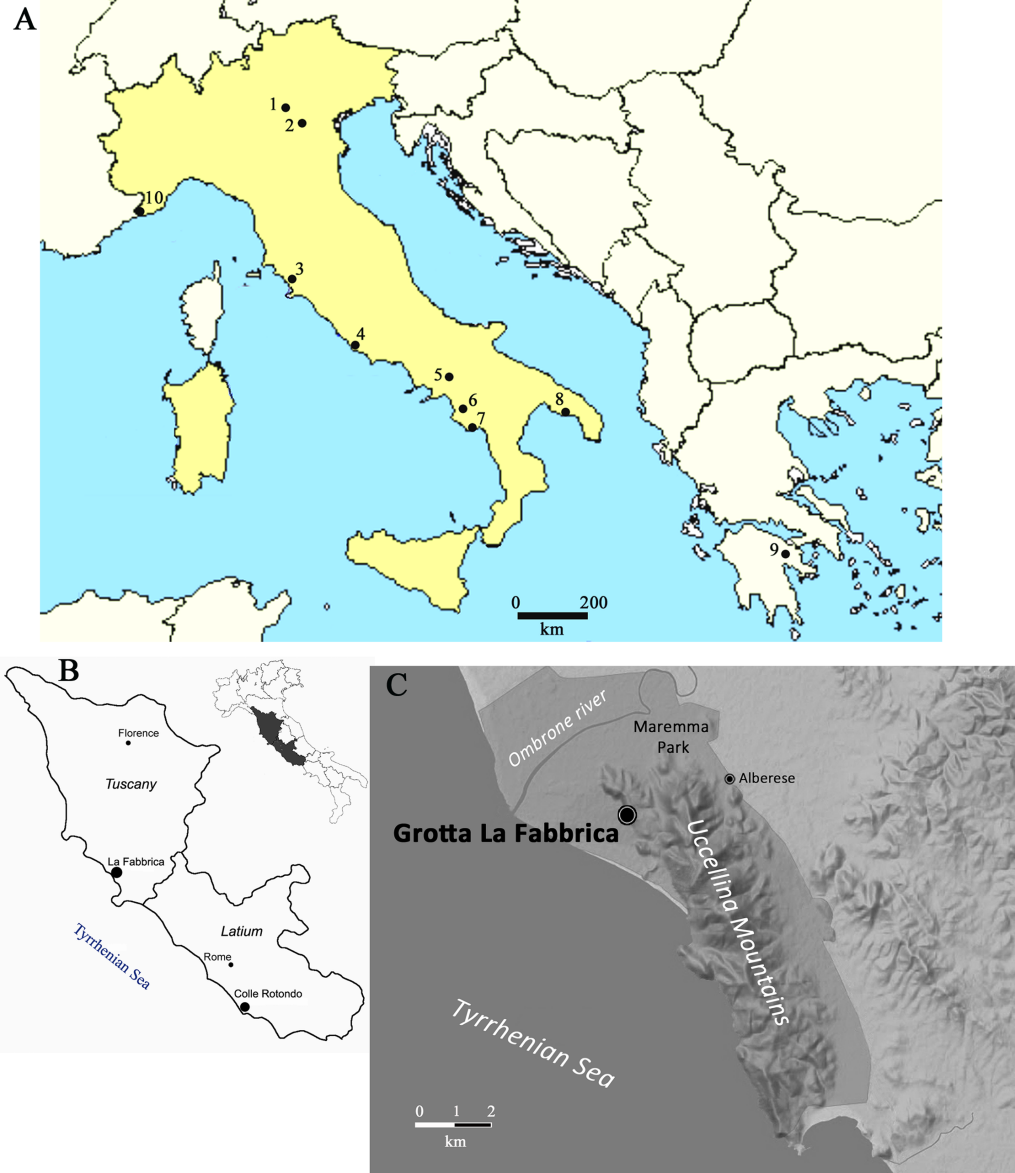
**(A) Map with location of main Uluzzian sites (1–4, 6–9) and some Protoaurignacian sites (5, 10) discussed in the text.** 1. Fumane; 2. Broion; 3. La Fabbrica; 4. Colle Rotondo; 5. Serino; 6. Castelcivita; 7. Grotta La Cala; 8. Cavallo, Grotta di Uluzzo, Grotta Bernardini; 9. Klissoura in the Peloponnese; 10. Riparo Mochi and Riparo Bombrini. (B) Map showing the location of Grotta La Fabbrica in southern Tuscany and of Colle Rotondo in Latium. (C) Detailed location of Grotta La Fabbrica, map by Iacopo Conforti modified from title figure in Dini et al. 2007 (S5 File).Maps 1A and 1B by Massimo Pennacchioni.

**Fig 2:**
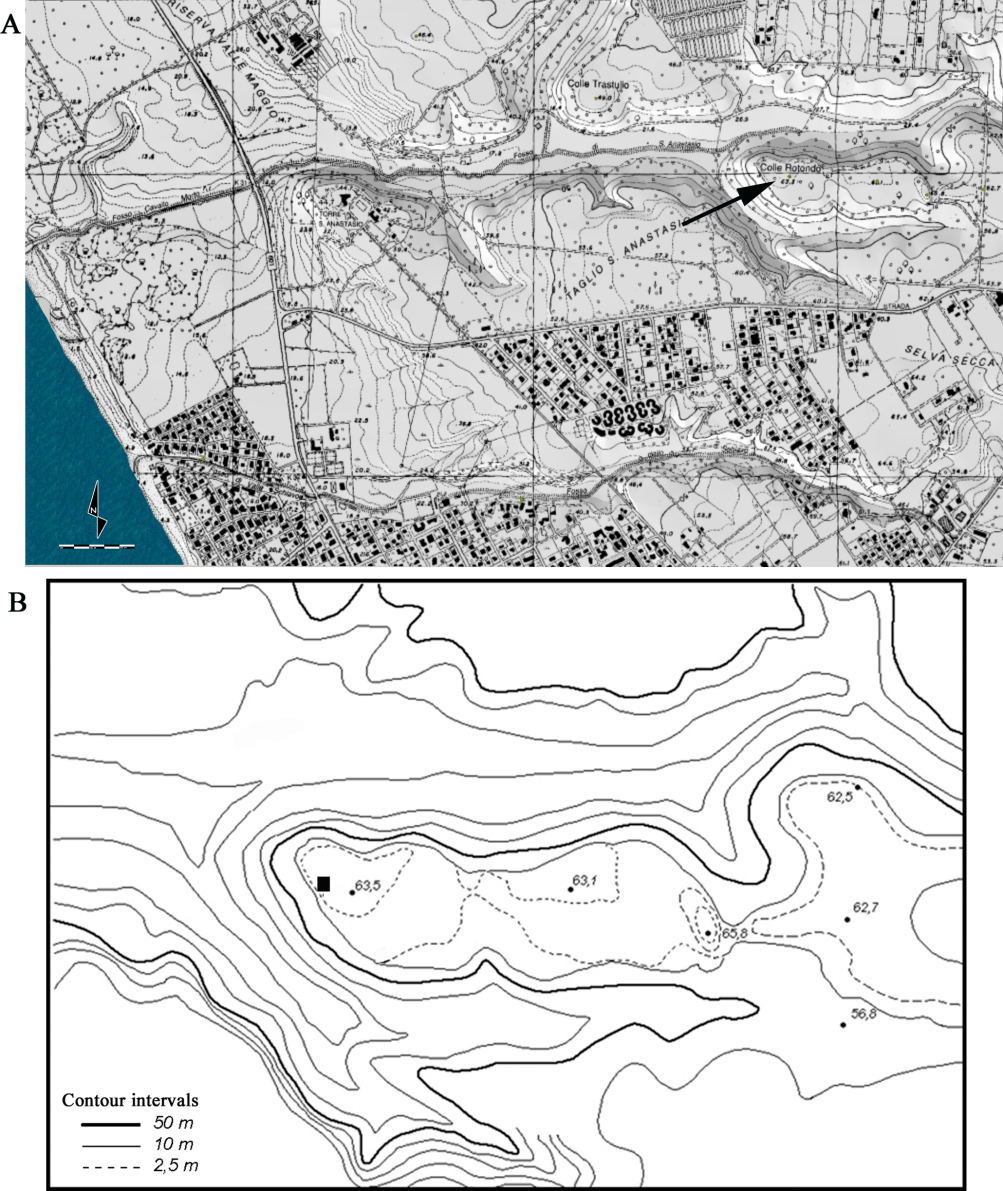
**(A) View of the Colle Rotondo area in southern Latium**. The black arrow points to the site location. Modified from a portion of a Latium map at 1:10,000 with 1 km grid (Lazio, CTR 10,000, 1990.91, F. 399080–399070; S5 File). Courtesy of Luca Galletti. The scale at bottom left has units of 50 m. (B) Detailed map of Colle Rotondo, the black square indicates the excavation area.

The Uluzzian is a cultural unit dated within the time interval between 45 and 40 ka B.P., the time interval during which Neandertals were replaced by a population of modern humans. It is known from a number of sites in Southern Italy (Fig 1): Castelcivita and Grotta La Cala in Campania, and Grotta del Cavallo, Uluzzo C and Grotta Bernardini in Apulia [1–5]. The Uluzzian occurs in northeastern Italy at the cave site of Fumane and at Riparo del Broion [6–8] but is absent in northwestern Italy (e.g. at Riparo Bombrini and Riparo Mochi in Liguria) [9]. In central Italy the Uluzzian is found at Grotta La Fabbrica (Tuscany) [10, 11] and at the open air site of Colle Rotondo (Latium). The two sites from central Italy are the focus of this paper (Figs 1 and 2). There is a long list of open air sites in Tuscany and other regions but most assemblages are surface collections that may represent a mixture of younger and older materials and are not considered here. Outside Italy the only published assemblage defined as Uluzzian comes from the cave site of Klissoura in the Peloponnese (Greece) [12–14].

Grotta La Fabbrica (42°39’14” N; 11°23’24” E) is located in the Parco Naturale della Maremma, at the base of the western slopes of Monti dell’Uccellina and 1.5 km from the Tyrrhenian Sea. Colle Rotondo (41°51’80 N; 12°60’74 E) is a single layer open-air site in south-central Latium. It lies on the flat top of a hill about 65.8 m asl and 2.3 km from the Tyrrhenian sea. It is a recent discovery proving the presence of the Uluzzian in a region where it was supposed to be completely absent [15, 16] and was excavated in 2011 and 2012 under the direction of Massimo Pennacchioni.

### The beginning of the Uluzzian

The Uluzzian overlies the Mousterian at all cave sites but not at the open air site of Colle Rotondo which contains a single layer only. Based on new radiocarbon results from Cavallo, Fumane, Castelcivita and Klissoura, a Bayesian statistical approach, and dates for the underlying Mousterian levels, Douka et al. [5] conclude that the Uluzzian appears in Italy by about 45,000 or shortly before. This is the time suggested by dates for Fumane. The oldest level of the Uluzzian sequence at Grotta del Cavallo (layer E III) could not be dated due to the absence of collagen in bones, lack of charcoal in the stored collection and scarcity of shells for dating. Douka et al [5] suggested that it may be older than Fumane. In 2013 the discovery of a Neandertal deciduous incisor in layer FII, which underlies layer E III, and is dated to a time range of 45,600–42,900 cal BP (at 68% of probability) [17] provided a terminus post quem for the bottom of the Uluzzian deposit. More recently geochemical analyses of three volcanic ash layers identified in the Mousterian and Uluzzian sequence of Grotta del Cavallo [18] have provided precise chronological markers. The three ash layers are: layer G in the middle of the Mousterian sequence, layer Fa at the Mousterian-Uluzzian boundary and layer CII above the Uluzzian. Of direct relevance here is the tephra of layer Fa. Chemical fingerprinting allows correlation of the tephra in Fa with the Green Tuff of Pantelleria volcanic island (southwest of Sicily) precisely dated by ^40^Ar/^39^Ar to 45.5 ± 1.0 ka. This date marks the beginning of the Uluzzian at Cavallo

The start of the Uluzzian at Castelcivita has not yet been established. There is, however, a radiocarbon date of 40.5–42.0 ka cal BP for the upper layer which agrees well with the stratigraphic position of the sample, below the Protoaurignacian. The youngest Uluzzian site appears to be Klissoura in Greece. Its thin Uluzzian layer is contaminated by earlier and later elements but the final date is provided by tephra of the Campanian Ignimbrite that occurs at the top of the Uluzzian layer [5].

### The end of the Uluzzian

Two important chronostratigraphic markers signal the end of the Uluzzian: the Protoaurignacian and the Campanian Ignimbrite. The Protoaurignacian is a culture-stratigraphic unit that appears in Southwestern Europe (France, northern Spain and Italy) at about 42–41 ka. The earliest appearance in Italy is at the Riparo Mochi in Liguria between 42.7 and 41.6 ka cal BP [5, 9]. Because of its similarity to the Early Ahmarian in the Near East, associated with modern humans, it was suggested that the Protoaurignacian reflected a modern human advance westward from the Near East [19]. The analysis of two deciduous incisors from the Protoaurignacian layers of Riparo Bombrini and Fumane [8] supports the idea that the makers of the Protoaurignacian were modern humans.

The Uluzzian underlies the Protoaurignacian at Fumane, La Fabbrica, Castelcivita and Grotta La Cala. In southern Italy the final stages of the Protoaurignacian at Serino and Castelcivita are marked by the Campanian Ignimbrite eruption. Northwest of Campania, however, the Campanian Ignimbrite does not occur for reasons explained below. Thus the end of the Protoaurignacian in Central and Northern Italy and its relation to the Early Aurignacian are not yet determined with certainty [20, 21].

The Campanian Ignimbrite (thereafter CI) is a major volcanic eruption that originated from the Phlegrean Fields west of Naples, in the Bay of Pozzuoli. The eruption is dated to 39.88 ± 0.12 ka [22]. CI ashes are found east or southeast of the Phlegrean Fields in southern Italy, in Mediterranean cores in the Ionian Sea and around Crete, east in Crvena Stijena in Montenegro, Klissoura in Greece, Temnata in Bulgaria and Kostienki in Russia. There are no known CI ash falls or cryptotephra in Latium (i.e. northwest of Campania), no CI tephra in the Central Apennines (where volcanic deposits have been intensively investigated [23] and no known occurrences in Tuscany and northwest Italy. The reason for the eastward distribution of CI ashes is that the dominant winds in the northern hemisphere are from west to east. The lack of CI ash-falls west of the Bay of Naples reflects the northwest orientation of the Italian peninsula. The trade winds blow from east to west but they are subtropical and blow over the Atlantic below 30 degrees of latitude.

The Campanian Ignimbrite overlies the Protoaurignacian at Castelcivita and Serino in Campania [24] and is directly above the last layer (layer D) of the Uluzzian sequence at Grotta del Cavallo and at Klissoura cave in the Peloponnese [5]. The chemical composition of layer CII, the last tephra layer in the Grotta del Cavallo sequence matches the chemistry of the Campanian Ignimbrite confirming previous suggestions that the CI occurred at Cavallo [18]. Based on the calendar chronology of several Italian sites with Uluzzian and Protoaurignacian levels [22] and the OSL date of the Uluzzian at La Fabbrica (40 ± 1.6 ka; see below section "La Fabbrica") it is clear that the Uluzzian disappeared sometime before the eruption of the Campanian Ignimbrite and was replaced by the Protoaurignacian. This is documented by the stratigraphies of Fumane, La Fabbrica, Castelcivita and Serino. Did the Uluzzian last longer at Grotta del Cavallo? According to Zilhao et al. [25] the occurrence of some Protoaurignacian elements in layer D of Cavallo (particularly Dufour bladelets) suggests that layer D was formed during Protoaurignacian times, with later elements (Aurignacian and Early Epigravettian) in derived position. The occurrence of Aurignacian artifacts in layer D is contested [18, 26] although the occurrences of Dufour bladelets, a typical Protoaurignacian artifact, are hardly mentioned. Nevertheless the controversy does not affect the chronology of the Uluzzian which clearly ended before 39 ka. After layer D, the cave site of Cavallo was abandoned for about 30,000 years until the final phases of the Paleolithic.

The objectives of our paper are: (a) to date the Uluzzian of Grotta La Fabbrica on the basis of newly acquired OSL dates and to place the open-air site of Colle Rotondo in its morpho-stratigraphic context; (b) to present the results of a detailed technological, typological and taphonomic analysis of the two lithic assemblages for a more precise characterization of their features; c) to view our results in the broader context of the emergence, duration and disappearance of the Uluzzian industries in Italy.

## Materials and methods

### Repositories

The La Fabbrica collections are housed in the Dipartimento di Civilta e Forme del Sapere, University of Pisa, under the care of Carlo Tozzi. The Colle Rotondo collection is housed in the laboratory of the Dipartimento di Studi Umanistici, University of Roma 3, under the care of Massimo Pennacchioni. Tozzi and Pennacchini are co-authors of this paper. Hence permits were not required for this study.

### Sorting and sampling

We followed the sorting procedures used by us in the analysis of other assemblages. We select all cores, core fragments, tools, tool fragments, complete or broken flakes preserving the platform, blades and bladelets, whether complete or fragments. Some debitage byproducts such as flakes from scaled pieces, Kombewa flakes and flakes with thermal scars are also recorded.

Our sorting procedures exclude from technological analysis flake fragments (broken flakes without the platform) flakes < 1 cm and chunks, Siret breaks, tested cobbles and cobble fragments. These materials are bagged by large categories and remain available for specific studies. Flakes between 1 and 2 cm are classed by raw material only but flakes from scaled pieces and bipolar flakes > 1 cm are extracted and counted in debitage tables. All other unretouched flakes preserving the platform are recorded in individual Excel files by type, raw material, platform type and measurements.

### Specimen numbers

The La Fabbrica lithics had square and layer indicated but no systematic catalogue number. Some pieces had numbers assigned occasionally for a particular research purpose. Thus we assigned individual numbers in sequential order to retouched pieces and cores which are individually bagged in reusable zipper bags (Minigrips) with preprinted labels.

The Colle Rotondo materials had year, square and sublevel marked in ink. Many had numbers but they were not in consecutive order by sublevel, the numbering was restarted by sublevel, a possible source of confusion. Hence we numbered them using sublevel as a prefix followed by the original number. The retouched pieces and cores are individually bagged with preprinted labels and stored in plastic drawers or larger plastic bags by class. Unretouched flakes and other small items have labels with their square and layer provenience and are bagged by large categories.

### Methods

Our analysis of lithic assemblages is directed to the identification and description of similarities and differences in reduction sequences, in the objective of production, in the selection of blanks for tool manufacture and in the typology of formal tools.

Once sorted with the procedures described above, retouched pieces (the terms small tools and retouched pieces are used interchangeably) and cores are recorded by catalogue number, provenience, raw material and state of preservation in separate Excel files. Length, breadth and thickness were measured in mm by digital caliper according to the morphological axis (if a tool) or the debitage axis (if a flake or a blade). Incomplete measurements (if the artifact is broken and incomplete) are identified by a symbol in front of the number. Raw material is identified by macroscopic examination with a 10x hand-lens and comparison with known geological samples.

Most analytical details (taphonomic attributes indicative of the state of preservation and formational history, attributes related to the mode of production such as types of blanks, type and percentage of cortex, type of flakes or blades, platform types, knapping accidents) are coded and described in the S3 File. Specific analytical details used in the classification of the Colle Rotondo and La Fabbrica unretouched flakes, formal tools and cores are also described in the S3 File. These artifacts are important for interassemblage comparison which is a main objective of this paper.

We read on each core and flakes the direction and sequence of removals thus reconstructing the manufacturing steps for each piece. On cores this information is represented in a visual way that reveals the general structure of the piece. The white lines indicate the scar outline; the scar orientation is indicated by a white arrow, a white dot indicates the presence of a negative bulbs. Repeating patterns of technical features allows the definition of technical types.

A dynamic understanding of each assemblage is provided by the ordering of static types into a sequence of manufacturing steps. This is the principle of chaînes opératoires which originated in the 1960s and came into common use in the 1990s [27]. The objectives of the lithic production are thus defined by categories counted in tables. These procedures, based on technical attributes, can be replicated.

#### Distinguishing scaled pieces from bipolar cores

Scaled pieces and bipolar cores are characteristic of the Uluzzian yet bipolar cores are often confused or lumped with scaled pieces (see section “Discussion”). Actually scaled pieces and bipolar cores can be distinguished on technical features because they result from different chaînes opératoires. The aim of bipolar cores is to produce flakes or blades to be used as such or retouched, while scaled pieces are made on flakes or blades and are retouched as a consequence of utilization. The bipolar technique consists in resting a core (a pebble, a block or a flake) on anvil and striking it with a percussor (Figure A in S3 File). This flaking technique can be used for initiating a reduction sequence. Flakes and cores produced with this technique are characterized by a non-conchoidal fracture, in contrast to most rocks knapped in prehistory, whether by direct or indirect percussion or pressure. Bipolar flakes and blades show crushing, splintering or shattering of the platform and of the opposite edge (Figure D: B-C in S1 File). Following [28–31] we define scaled pieces as having at least one thin, chisel-like edge and showing traces of bipolar percussion on anvil, i.e. crushing and splintering of edges at opposite ends. The edge opposite to the chisel-like edge may present a flat platform but in intensively reduced pieces the platform is shattered. They may be made on flakes or blades, have a thin, often lenticular section and are generally interpreted as intermediary pieces to split materials such as bone or wood. In other words scaled pieces are tools by utilization, not by retouch; they are not present in Bordes’ typology but are defined in Upper Paleolithic industries [29]. Some of the bipolar flakes were used as blanks for retouched pieces while the byproducts of scaled pieces were never retouched. Flakes produced during use of scaled pieces are generally small (generally < 3 cm), have a portion of chisel-like edge and smalls step scars and splintering in the impact area or the distal end (Fig D: D-E in S1 File).

Bipolar cores are generally thicker with flake scars extending the full length of the core and detached from several faces; the striking platform is rarely preserved and one or both ends display battering with step fractures (Fig D:A and F:A-C in S1 File).

## Results

### La Fabbrica

**Fig 3:**
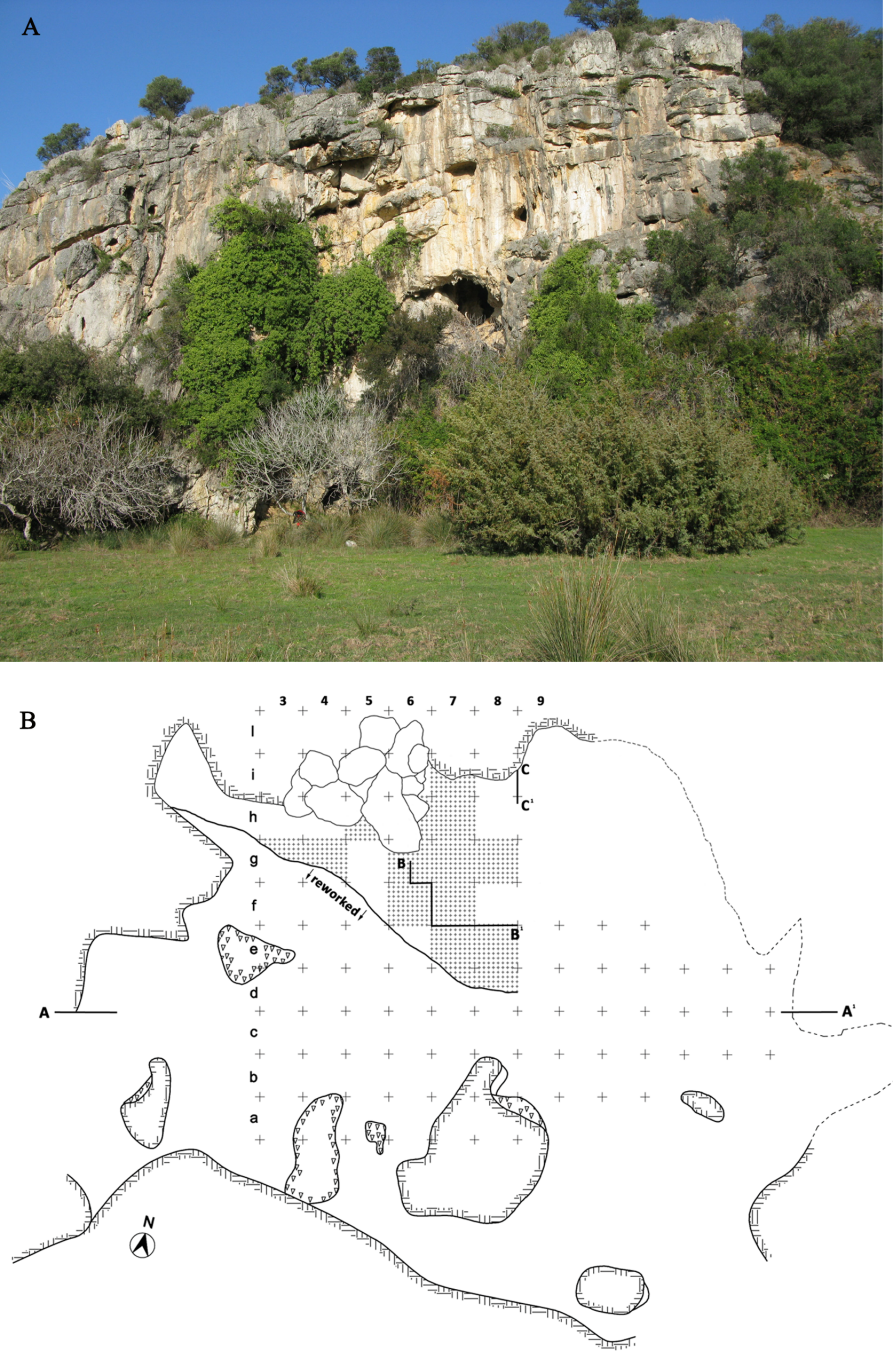
**(A) View of Grotta La Fabbrica**. **(B) Plan of the excavation with 1 m grid**. The squares with a dotted pattern have yielded Uluzzian material in situ, studied in this paper. Materials from reworked deposits south of the black line have been excluded from analysis.

Grotta La Fabbrica opens 7 m above the coastal plain on a cliff of massive limestone (Fig 3A). The cave opening is now receded with respect to the original entrance due to cliff erosion, documented by stalagmites and portions of cemented fillings outside the entrance. Faults in the karstic system and connections with underlying cavities have created an alternation of erosion and redeposition and the cave deposits are sloping forward. A large part of the deposits are redeposited and only deposits near the northern wall of the cave are in situ. Our analysis is based on a complete taphonomic revision of the cave deposits and assemblage integrity based on excavations record. All reworked materials have been excluded from the analysis.

Following preliminary exploration in 1964–65, systematic excavations were carried out under the direction of C. Tozzi of the University of Pisa between 1969 and 1973 [10]. The reworked deposits were removed very cautiously and the intact deposits preserving in situ materials were mapped. The stratigraphic sequence includes layer 1 at the base with Mousterian artifacts, layer 2 with Uluzzian, layers 3–4 with Protoaurignacian and a small remnant of a layer almost completely destroyed by erosion containing Gravettian and Epigravettian materials.

Layers 1 and 2, studied in this paper, are made of sandy silts with clasts of decalcified limestone. The associated animal bones are not well preserved, mostly fragile and teeth are the most abundant elements [10].

**Fig 4:**
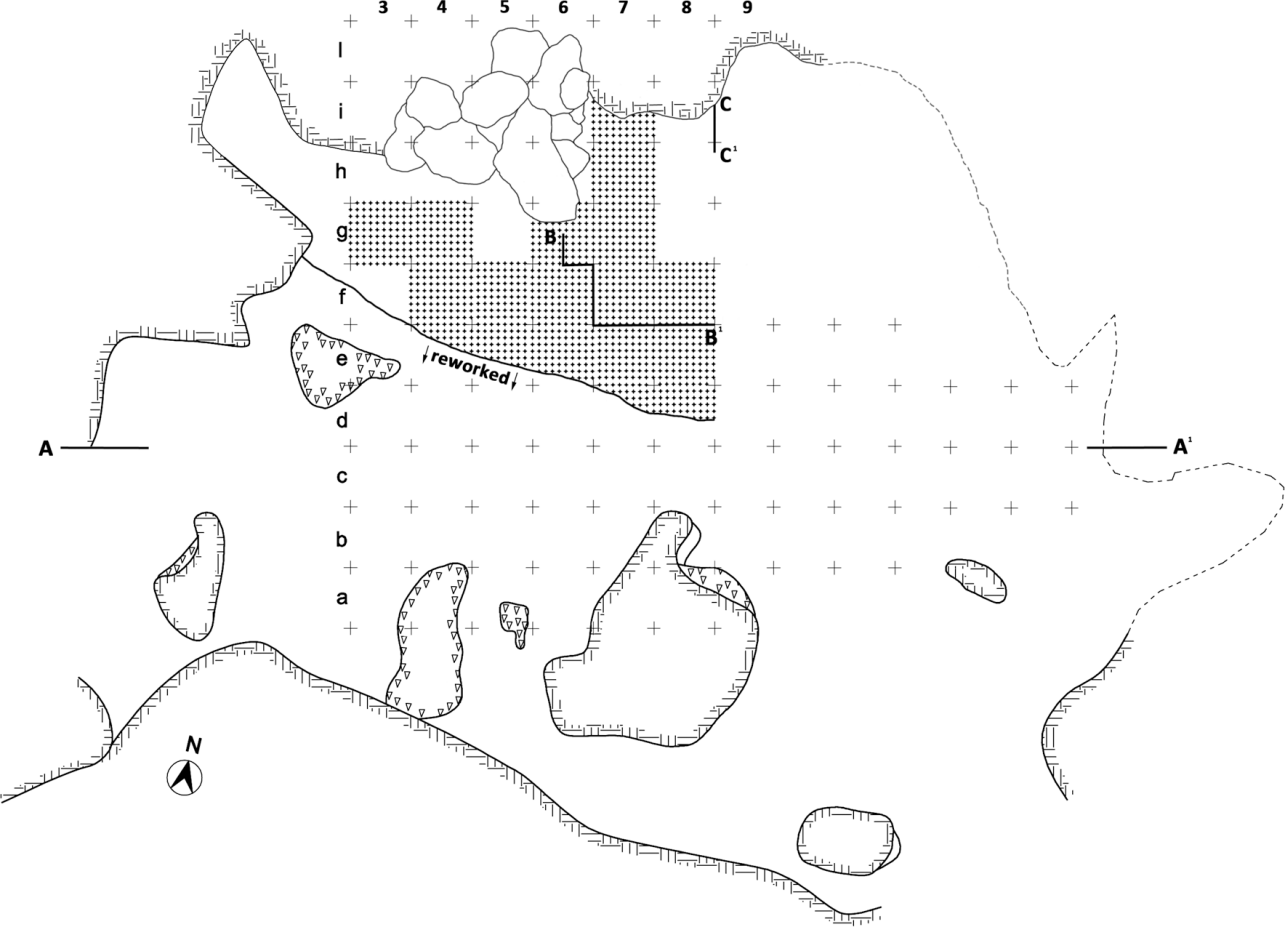
La Fabbrica. **Plan of the excavated squares with Mousterian materials in situ (dotted pattern) studied in this paper.** The Mousterian layer 1 (1a -1c) rests on the bedrock. Sections A-A^1^, B-B^1^ and C-C^1^ are illustrated in Fig 5.

**Fig 5:**
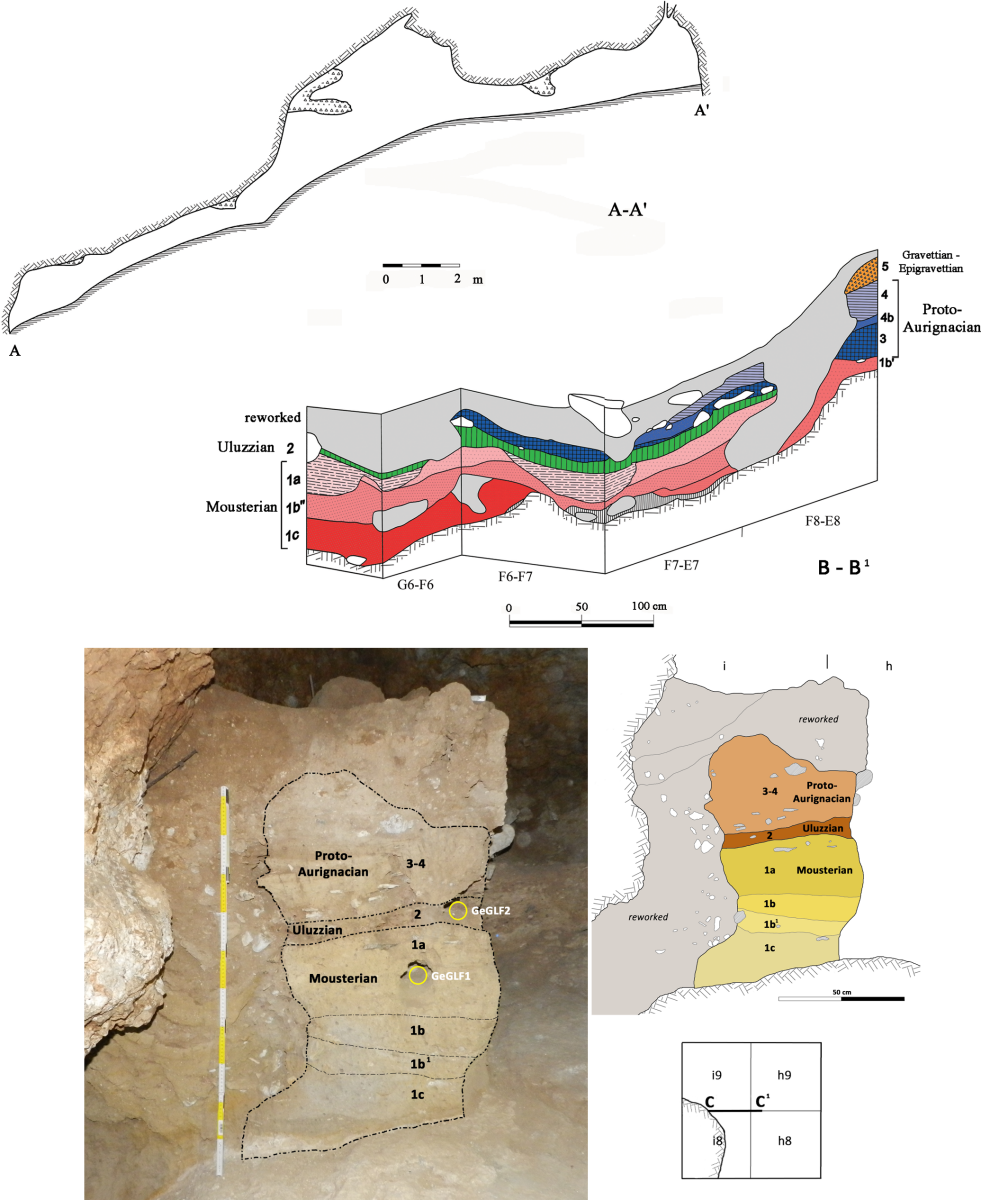
La Fabbrica sections. Section A-A^1^. The cave floor slopes of about 30 degrees to the west, the ceiling is generally low. Section B-B^1^ illustrates the sequence of occupations. Section C-C^1^ shows the location of the two OSL samples taken in intact deposits in 2012.

Two OSL dates were obtained in the Mousterian and Uluzzian layers by the laboratory of the University of Milano-Bicocca (Italy; L. Panzeri, M. Martini, E. Sibilia). The Mousterian is dated to 44 ± 2.1 and the Uluzzian is dated to 40 ± 1.6 ka (S2 File). An erosional surface separates the Uluzzian from the underlying Mousterian. Our technological and typological analysis of the Mousterian and Uluzzian layers is based exclusively on the in situ material (Figs 4 and 5), checked by a combination of spatial data, and excavation records. Our artifact counts are less than those previously published for the Uluzzian assemblage [10]. The older counts incorporated artifacts from reworked areas. However, it is fair to say that these differences do not affect the general assemblage diagnoses as the reworked and in situ materials show substantial homogeneity.

We had originally planned to study in the same way the Protoaurignacian of which a technological and typological analysis was published in 2012 [32] based on materials from 11 squares from the 1969–1973 excavations and on renewed excavations by Dini starting in 2008. Our counts of the published materials show that the assemblage consists of about 673 pieces. During analysis it became clear that the assemblage, as published, actually contains artifacts from reworked squares. Thus in [32] figures 7, 9 and 10 illustrate 14 artifacts of doubtful provenience. Many artifacts derive from squares that were only partly reworked. Careful reading of the excavation journals suggests that it is in fact possible to correctly identify the in situ artifacts and separate them from the reworked pieces. However, this represents a very large amount of work since it has to be done one piece at the time. We have therefore regretfully excluded the Protoaurignacian from this work.

#### La Fabbrica Mousterian, layer 1

The layer rests on bedrock. During excavation it was subdivided in 4 levels (1a,1b, 1b^2^, 1c) based on differences in color and the presence of discordant surfaces. The in situ material comes from approximately 15 sq m and was in part protected from erosion by large blocks fallen from the roof (Fig 4). The material from the in situ and from the redeposited squares (Fig 4) appears to be essentially homogeneous [24] but we chose to study only the in situ material which we treated it as one layer (Table 1). The OSL sample taken in level 1a (underlying the Uluzzian level) was dated to 44 ± 2.1 ka BP (Fig 5 section C-C^1^; S2 File).

**Table 1:** La Fabbrica Mousterian, layer 1. Assemblage composition.

Classes of artifacts, all raw materials (quartz specimens in parenthesis)	N	%
Debitage	117 (10)	53.7
Retouched pieces	76 (0)	34.9
Cores and core fragments	25 (3)	11.5
Total	218	
		
Chunks, flakes < 2 cm, flake fragments, whole cobbles, Siret breaks	189	

Table 1 shows the classification of the assemblage into major artifact categories. Chunks, flakes <2 cm, flake fragments (flakes without the platform) and Siret breaks are listed but not included in the analysis. All the materials are fresh.

**Raw material**. The majority of artifacts are made on pebbles from the alluvial deposits of the Ombrone river which is about 1.5 km from the site. The main raw materials are jasper, flint or chert, quartzite and siliceous limestone (Table 2). Following the example of other archaeologists, we distinguish flint as characterized by a fine, homogeneous glossy texture with smooth surfaces and chert as being coarser-textured and opaque. Jasper can be fine-textured and coarse-textured, the two types may occur on the same cobble or block. It often has fissures and fracture planes. Only a small number of jasper artifacts show a type of cortex indicating primary sources about 10 km or less from the site [11]. Veins of milky quartz were available in the immediate vicinity of the cave; it occurs in the form of slabs or slightly rounded blocks with many fracture planes and is often very coarse-textured. Rock crystal is extremely rare. The poor quality of quartz has considerably influenced the production of supports, so that retouched artifacts are very poorly represented and badly defined [33].

**Table 2:** La Fabbrica Mousterian, Layer 1. Counts of raw materials by main artifact classes.

Raw material	Debitage	%	Small tools	%	Cores and core fragments	%
Jasper	72	61.5	56	73.7	10	40.0
Flint	8	6.8	7	9.2	2	8.0
Chert	13	11.1	11	14.5	7	28.0
Siliceous limestone	9	7.7	1	1.3	0	0
Quartzite	5	4.3	1	1.3	3	12.0
Quartz	10	8.5	0	0	3	12.0
Total	117		76		25	

**Fig 6:**
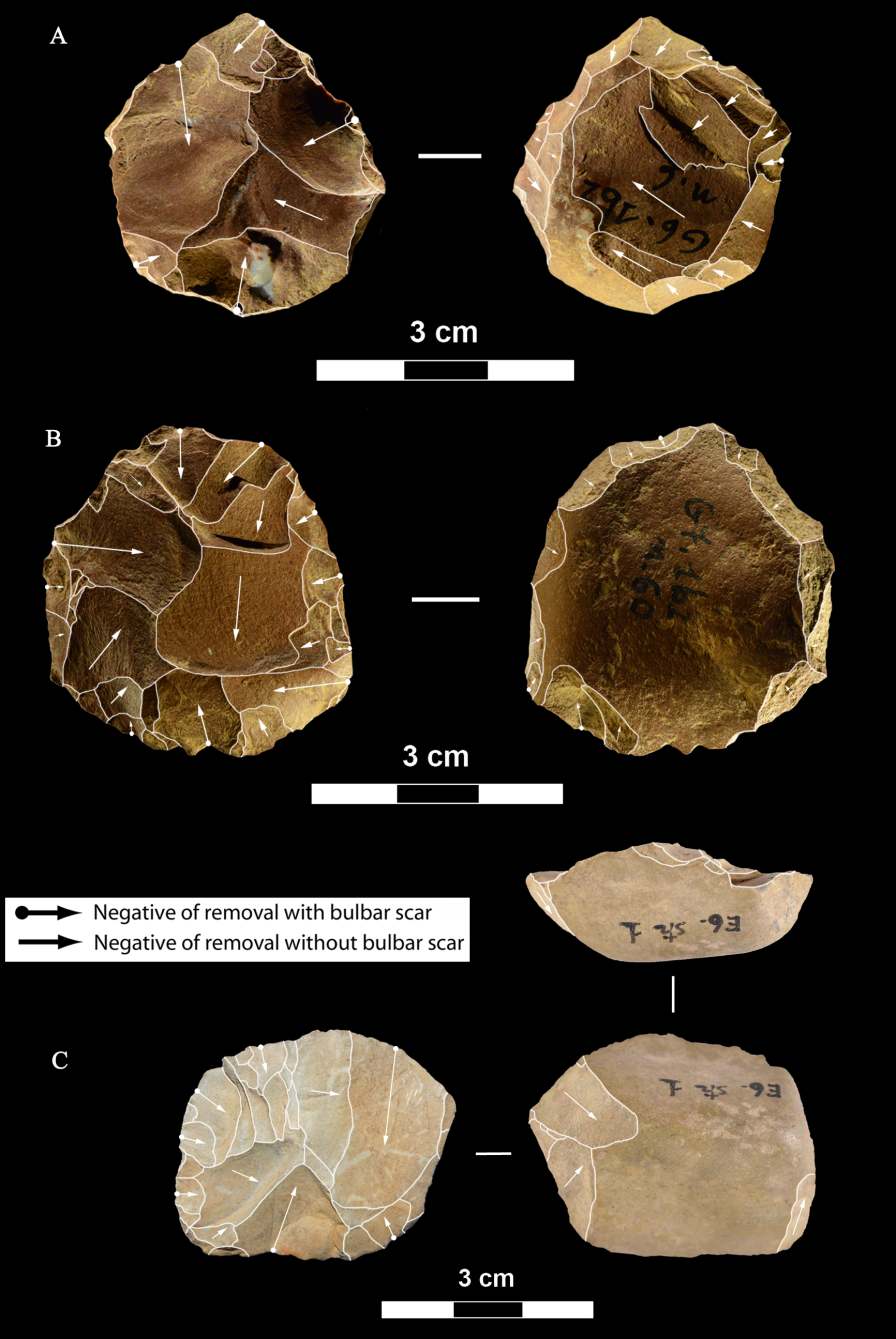
La Fabbrica, Mousterian cores from layer 1. Cores on small flint pebbles and of Levallois recurrent centripetal type. Maximum Length (L) of A, B, C: 35, 39 and 42 mm. Catalogue number 4, 6, 21. For symbolic conventions used in this and other figures see S3 File.

**Fig 7:**
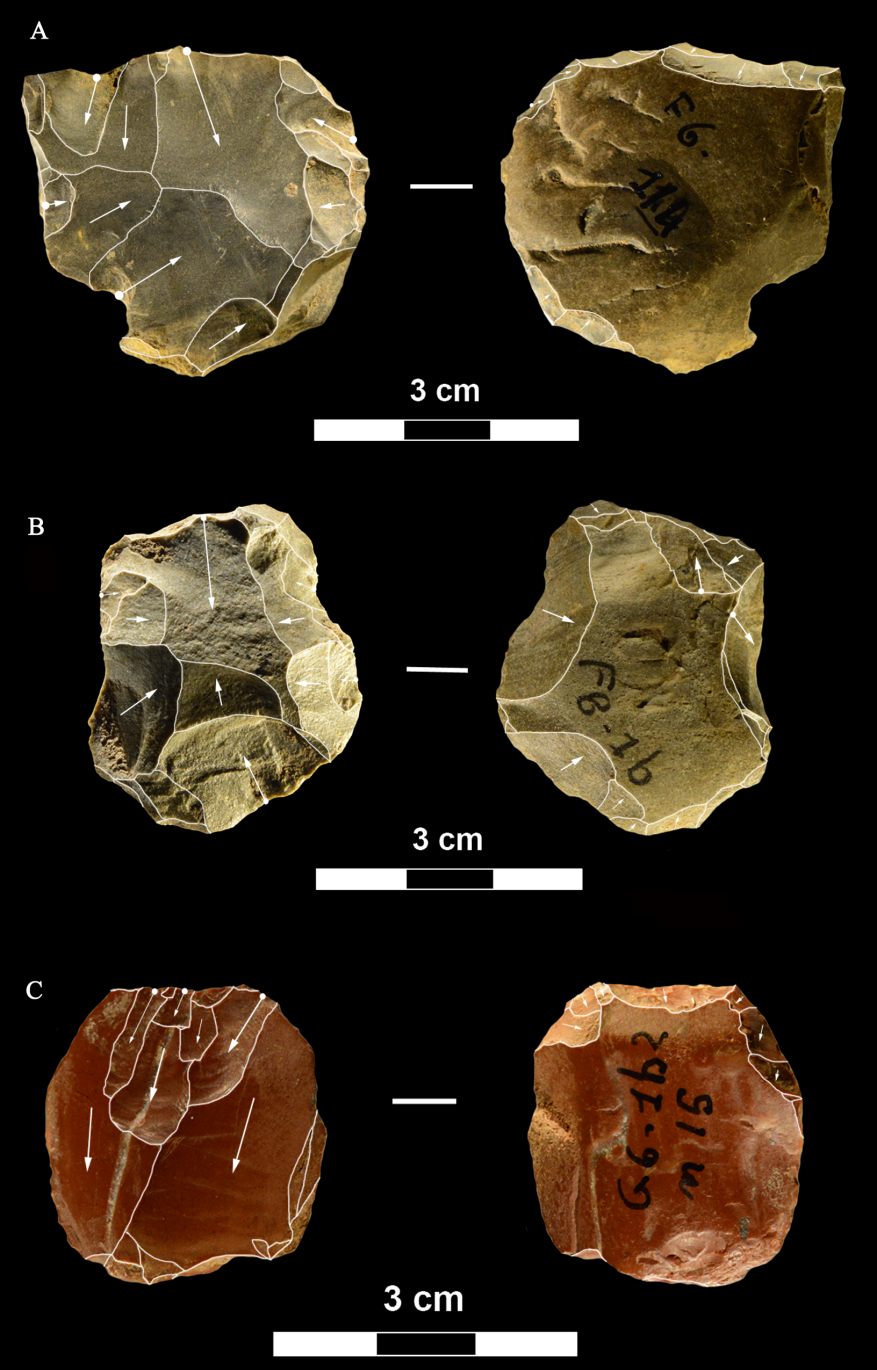
La Fabbrica, Mousterian cores from layer 1. (A-B) Levallois recurrent centripetal cores on a flint and a quartzite pebble. (C) Unidirectional core on a single debitage surface and partial platform preparation but no convexities, on a pebble of fine-grained jasper. L of A, B, C: 37, 36 and 30 mm. Catalogue numbers: 1, 2, 3.

**Fig 8:**
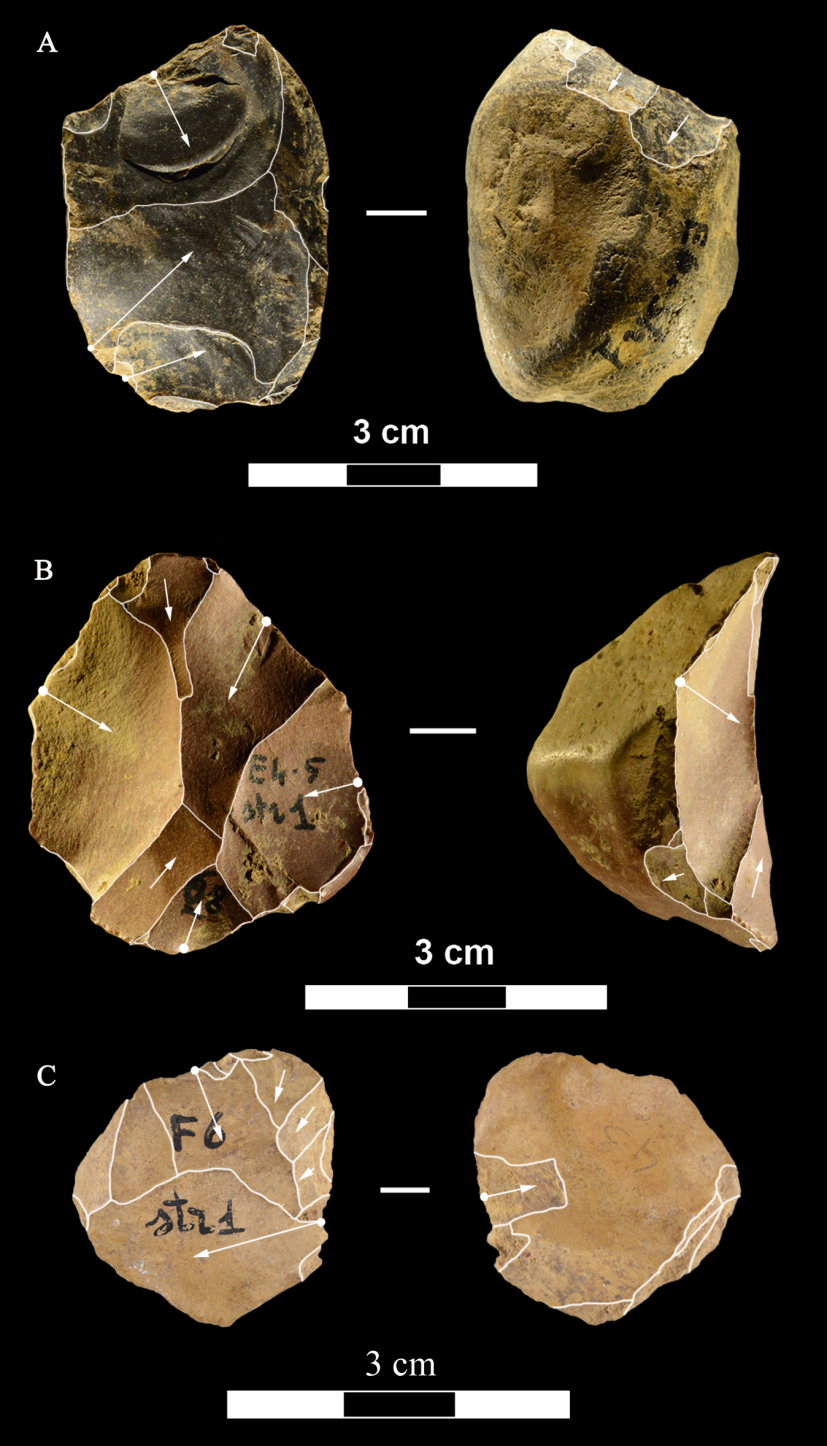
La Fabbrica, Mousterian cores from layer 1. (A) Bidirectional core of uncertain Levallois character on a flint pebble; note the partial platform preparation and presence of convexities on two sides only. (B) Centripetal (non-Levallois) core with a single debitage surface on a jasper pebble, front and left side view. (C) Core of recurrent Levallois type on a chert pebble; the last scar is invasive and seems to have removed the convexities. L of A, B, C: 39, 40 and 23 mm. Catalogue numbers: 7, 5 and 22.

**Core types**. Levallois cores are the most characteristic component of lithic production in this layer which also includes a simpler kind of cores without any special preparation of the debitage surface or shaping of the core (Table 3, Figs 6–8). The most common raw material is jasper (40%) followed by microcrystalline silica (36%), quartz and quartzite are 12% each.

**Table 3:** La Fabbrica, Mousterian, layer 1. Counts of cores.

Core type (quartz specimens in parenthesis)	N	%
Levallois core with recurrent centripetal removals	6	24.0
Levallois recurrent centripetal with a final large scar	1	4.0
Core with bidirectional removals, the Levallois character is uncertain	1	4.0
Core with centripetal (non-Levallois) removals	1	4.0
Core with unidirectional removals	5 (2)	20.0
Core with multidirectional removals	1	4.0
Core with bidirectional removals	1	4.0
Core fragments	5	20.0
Undetermined core	4 (1)	16.0
Total	25	

**Debitage.** There are very few unretouched Levallois flakes (Table 4 and Fig A: A, B, D in S1 File) but they are complemented by six Levallois flakes retouched into scrapers or denticulates (Table 5). Knapping was done by direct internal (hard hammer) percussion; the bipolar percussion is rare (mainly documented in quartz, as could be expected). Blades and bladelets are even more rare and were not retouched and used as tool blanks. Types of flakes produced by the bipolar technique and by direct percussion from unprepared cores are described in “Methods” and in the S3 File.

**Table 4:** La Fabbrica Mousterian, layer 1. Counts of flakes and blades.

Debitage (quartz specimens in parenthesis)	N	%
Levallois flakes	3 (0)	2.6
Flakes from non-Levallois cores, with unidirectional, bidirectional, orthogonal or convergent dorsal scars	37 (0)	31.6
Pseudo-Levallois points and *débordant* flakes	7 (0)	6.0
Centripetal flakes	4 (0)	3.4
Ordinary cortical flakes	12 (0)	10.3
Ordinary partly cortical flakes	25 (5)	21.4
Ordinary non-cortical flakes	12 (1)	10.3
Bipolar flakes	4 (3)	3.4
Blades	2 (0)	1.7
Bladelets	3 (1)	2.6
Flakes from scaled pieces	2 (0)	1.7
Undetermined flakes	6 (0)	5.1
Total	117	

**Table 5:** La Fabbrica Mousterian, layer 1. Counts of small tools.

Tool type	N	%
Scrapers (including side, transverse and convergent scrapers)	24	31.6
Denticulates and notches	16	21.1
Bec	1	1.3
Retouched and utilized	33	43.4
Tool fragments	2	2.6
Total	76	100.0

**Small tools**. The small tools are essentially scrapers and denticulates (Table 5 and Fig 1: E-I in S1 File). There are no scaled pieces but eight are present in the reworked materials which consist of about 700 pieces [34]. Flakes are the only kind of tool blanks, including six Levallois flakes.

**Concluding remarks on the La Fabbrica Mousterian**. There are essentially only two well documented chaînes opératoires: 1. a recurrent centripetal Levallois reduction sequence whose products were preferentially selected for retouch, and 2. A simpler kind of reduction sequence without any special preparation of the debitage surface or shaping of the core and with series of unidirectional, bidirectional or centripetal scars, some of which are similar to those found in the Uluzzian layer. This kind of simple core occurs throughout the Paleolithic. The bipolar technique is represented by just a few flakes.

Detailed comparisons with the following Uluzzian assemblage cannot be done since the Mousterian series is too small.

#### La Fabbrica, Uluzzian, layer 2

Layer 2 contains an abundant industry and is dated by OSL to 40 ±1.6 ka BP (S2 File). It consists of clayey silts with dispersed charcoal bits. The faunal material is fragile and consists mainly of teeth [10]. Only Uluzzian materials in situ are studied in this paper (Fig 3B; Tables 6 and 7).

**Table 6:** La Fabbrica Uluzzian. Assemblage composition.

Classes of artifacts, all raw materials (quartz specimens in parenthesis)	N	%
Debitage	405 (88)	66.6
Small tools	113 (14)	18.6
Cores	90 (20)	14.8
Total	608	100.0
Chunks, flakes <2 cm, flake fragments, tested and whole cobbles, Siret breaks, flakes with thermal scars. Bipolar flakes and flakes from scaled pieces 1–2 cm are included in debitage counts.	1221 (290)	

**Table 7:** La Fabbrica, Uluzzian. Counts of raw materials by main artifact classes.

Raw material	Debitage N	%	Retouched pieces N	%	Cores and core fragments N	%
Jasper	265	65.4	84	74.3	66	73.3
Flint	13	3.2	9	8.0	2	2.2
Chert	30	7.4	4	3.5	1	1.1
Sil Lim	6	1.5	1	0.9	1	1.1
Quartzite	3	0.7	1	0.9	0	0.0
Quartz	88	21.7	14	12.4	20	22.2
Total	405	100	113	100.0	90	100.0

**Fig 9:**
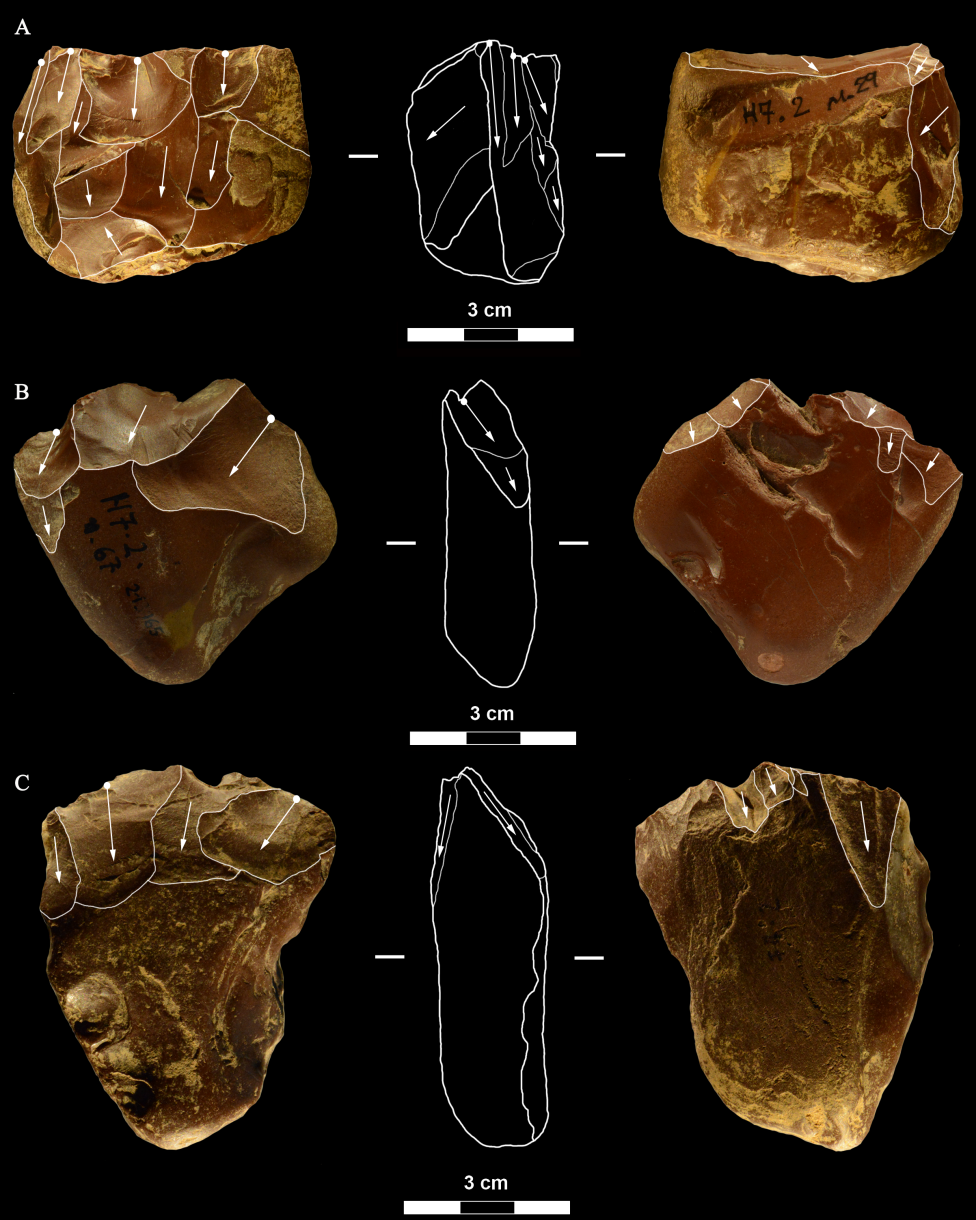
La Fabbrica, Uluzzian cores on jasper pebbles, front, left profile and back views. (A) Unidirectional core with a series of parallel removals on a single debitage surface, a large scar forms the platform. (B and C) Unidirectional cores with multiple platforms, passing to bifacial cores. L of A, B, C: 51, 59 and 69 mm. Catalogue numbers: 14, 2 and 3.

**Fig 10:**
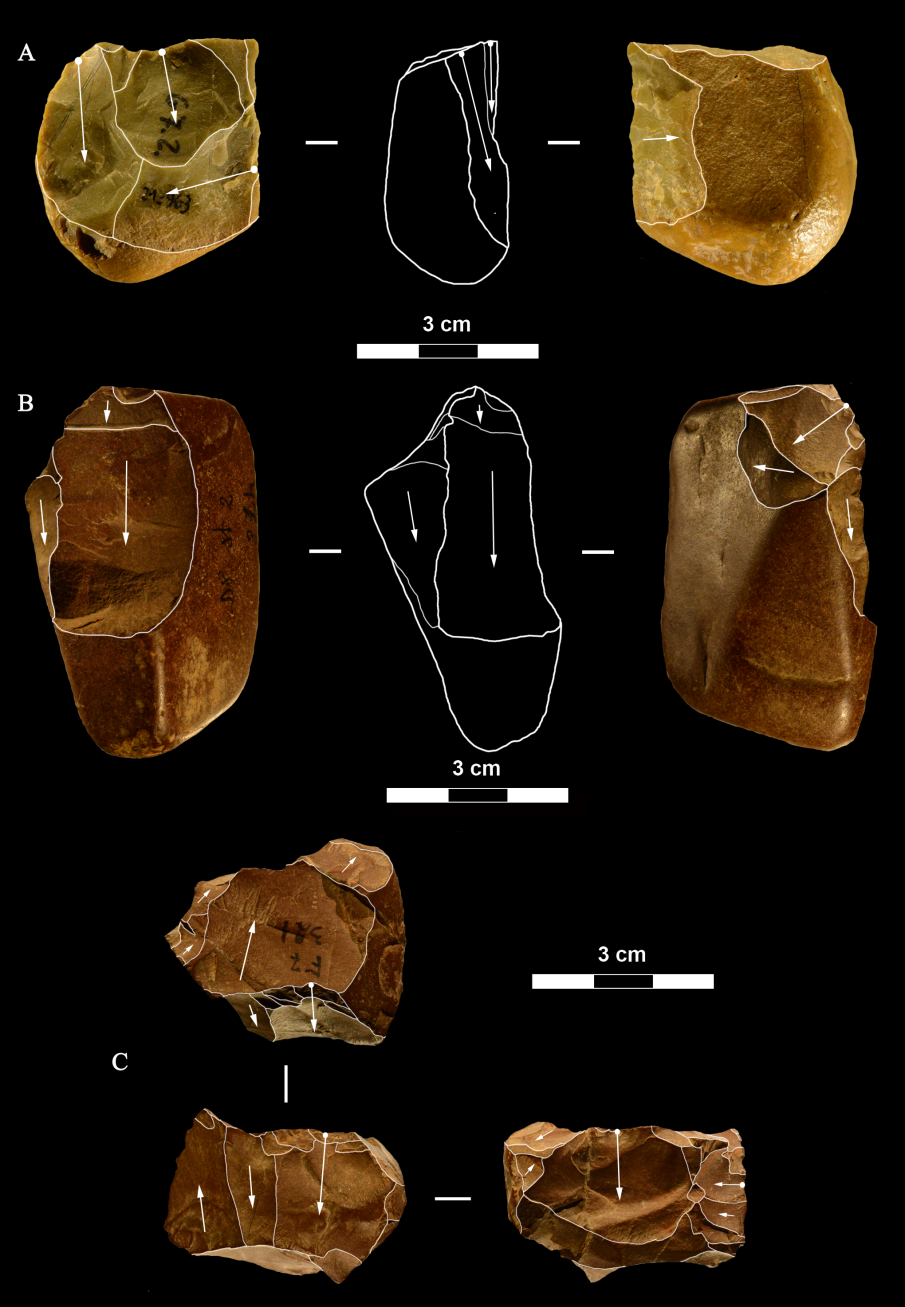
La Fabbrica. **(A) Uluzzian cores on chert (A) and jasper (B, C) pebbles**. (A) Unidirectional core with orthogonal removals on the same debitage surface. Platforms are a fracture surface (top) and a large scar (side). (B) Unidirectional core with parallel removals and with orthogonal removals on adjacent surfaces. Previous scars form the platform. (C) Multidirectional core with three debitage surfaces. L of A, B, C: 39, 60 and 39 mm. Catalogue numbers: 33, 18 and 12.

**Fig 11:**
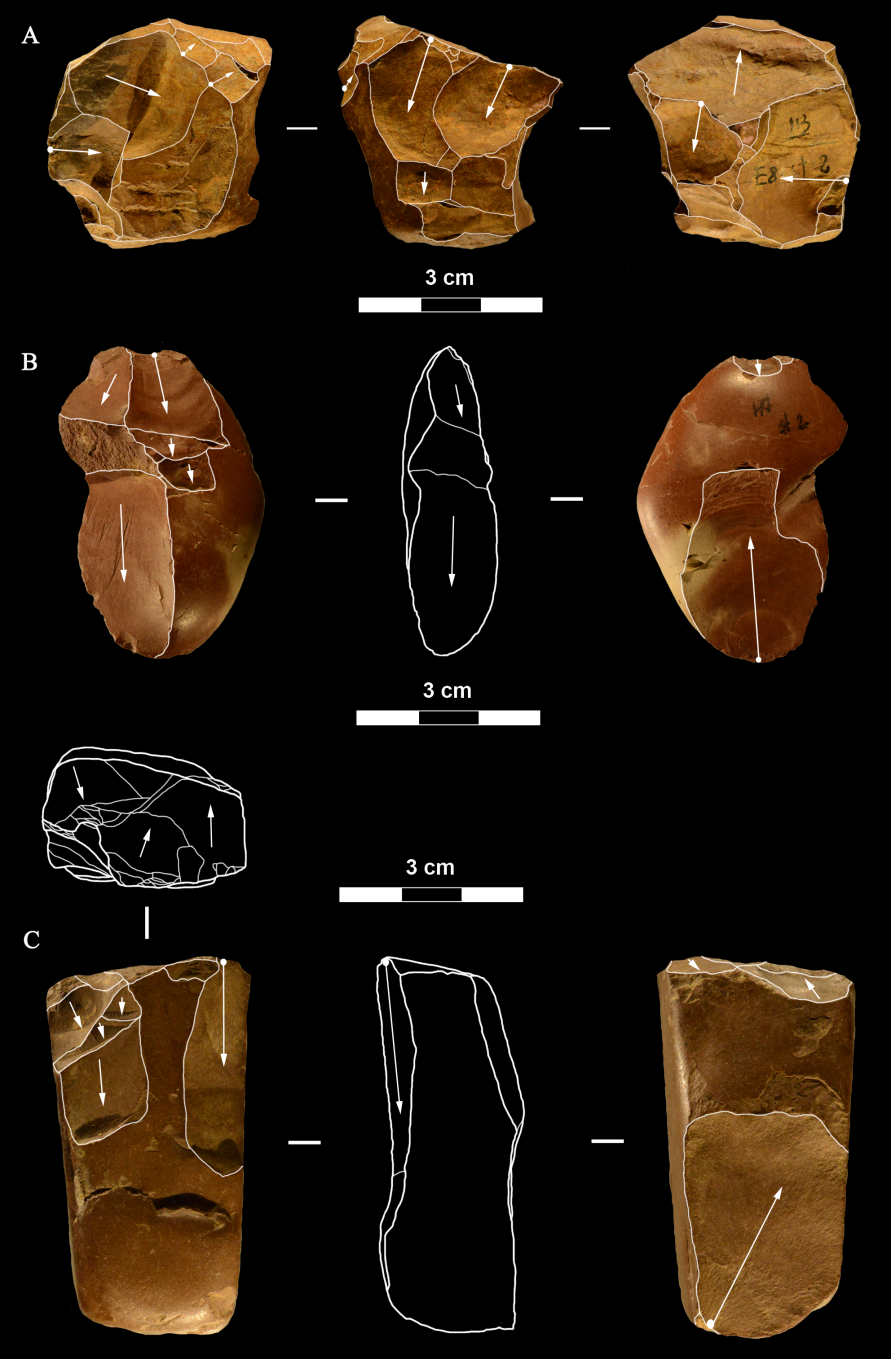
La Fabbrica, Uluzzian cores on jasper pebbles. (A) Multidirectional cores with three debitage surfaces, multiple previous scars are the platform of subsequent removals. (B) Bipolar core, products are flakes and one laminar flake. (C) Bipolar core, one removal is a cortical blade (31 x 11 mm). L of A, B, C: 40, 50 and 61 mm. Catalogue numbers: 16, 49, 13.

**Core types.** There are no Levallois cores and no discoid cores. The most common components are unidirectional cores with parallel removals on a single debitage surface or two adjacent debitage surfaces and a platform formed by a single scar or from previous scars on the opposed surface (n = 13; Fig 9); some have orthogonal removals on one or two debitage surfaces (n = 5; Fig 10A and 10B). Multidirectional cores with three or four debitage surfaces are less common (n = 6; Figs 10C and 11A). Simple debitage from unprepared cores occurs throughout the Paleolithic. The raw material (mainly jasper) is rarely homogeneous and has many fissures and fracture planes; this is also indicated by the fact that six cores are undetermined because of knapping failures.

Nevertheless the same kind of raw material did not stop Mousterian knappers from making Levallois cores, so the choice of simple knapping procedures seems deliberate. Sequences of reduction are relatively short, as indicated by the fact that 65% of ordinary flakes or flakes from unidirectional or bidirectional cores are cortical or partly cortical. (179/277)

**Fig 12:**
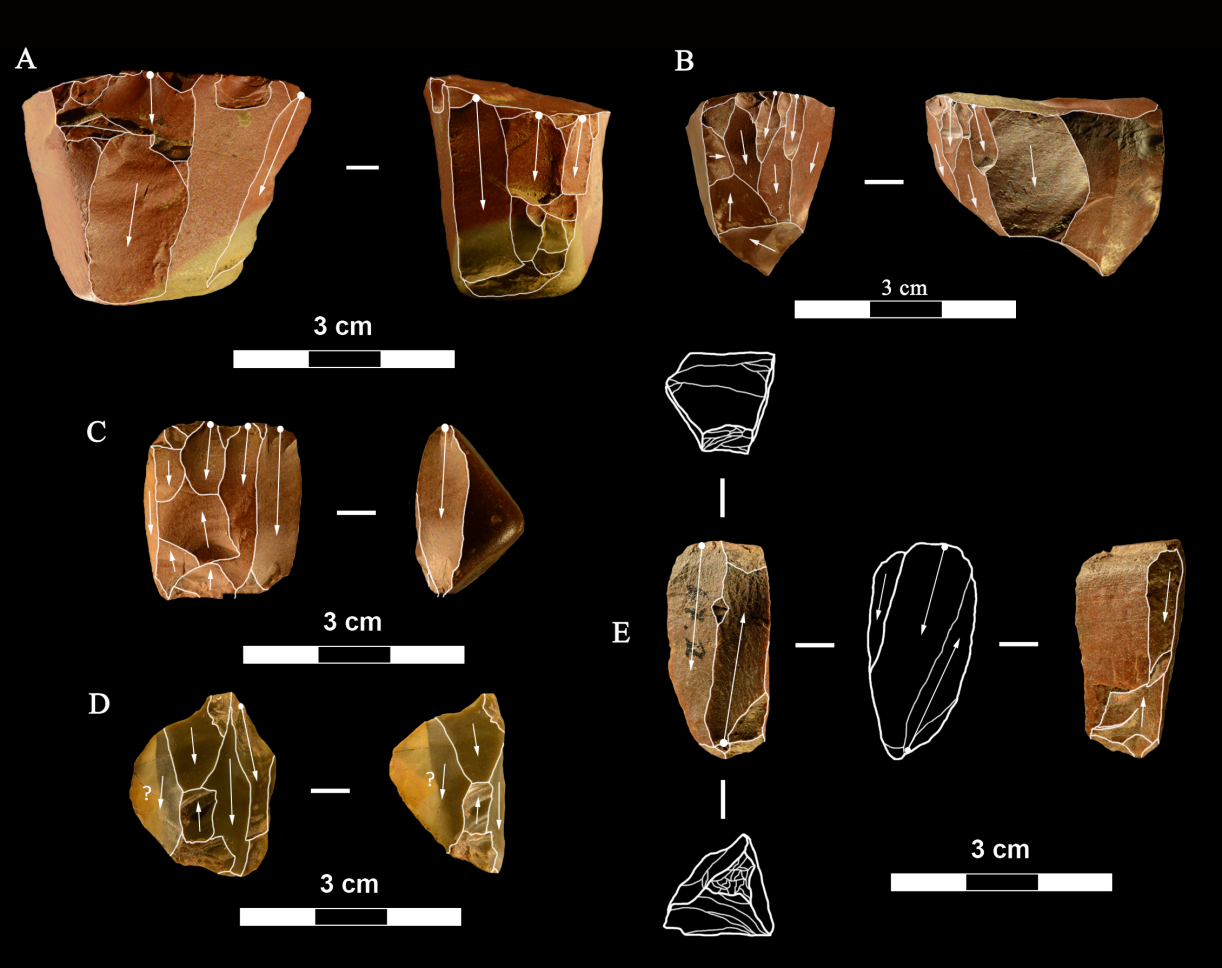
**La Fabbrica, Uluzzian bladelet cores on jasper pebbles (A-C, E) and flint (D).** (A) and (B) have semi-rotating removals by direct percussion and a single platform which is a natural fracture surface. (C), (D) and (E) are bipolar cores. (A) the last scar is 28 x 10 mm. (B) the last two scars are 9.2 x 3 and 11 x 3.5 mm. (C) Bipolar core with a single debitage surface, the last scar is 22 x 9.5 mm. (D) Bipolar core, the last scar = 19.7 x 8 mm. (E) Bipolar core, L = 29 mm, last scar 24 x7 mm. L of A-E: 40, 31, 24, 26, 29 mm. Catalogue numbers: 5, 29, 54, 55, 51.

Bipolar cores are quite abundant (Table 8; Fig 11B and 11C) and occasionally produced laminar flakes and bladelets (when close to being exhausted) although flakes were the most common products. Bladelet cores and bipolar bladelet cores (Fig 12) are evidence of intentional if sporadic bladelet production; blades do not come from dedicated blade cores but were produced by unidirectional or bipolar cores in the first stages of reduction. Excluding core fragments, there are 37 bipolar cores and 34 cores by direct percussion. However bipolar products were not or were rarely selected as tool blanks (see below).

**Table 8:** La Fabbrica, Uluzzian. Counts of cores.

Core type, all raw materials (quartz specimens in parenthesis)	N	%
Core with unidirectional or bidirectional removals on a single or on adjacent debitage surfaces	26 (8)	28.9
Cores with multidirectional removals on three or more debitage surfaces	6	6.7
Bipolar cores and fragments^a^	39 (8)	43.3
Bladelet core	2	2.2
Bipolar bladelet core	3	3.3
Unclassified core fragments	8 (3)	8.9
Core of undetermined type^b^	6 (1)	6.7
Total	90	100

^a^ some produced flakes and bladelets or laminar flakes. There are five bipolar core fragments.

^b^ failed due to bad raw material with many fissures or fracture planes

**Fig 13:**
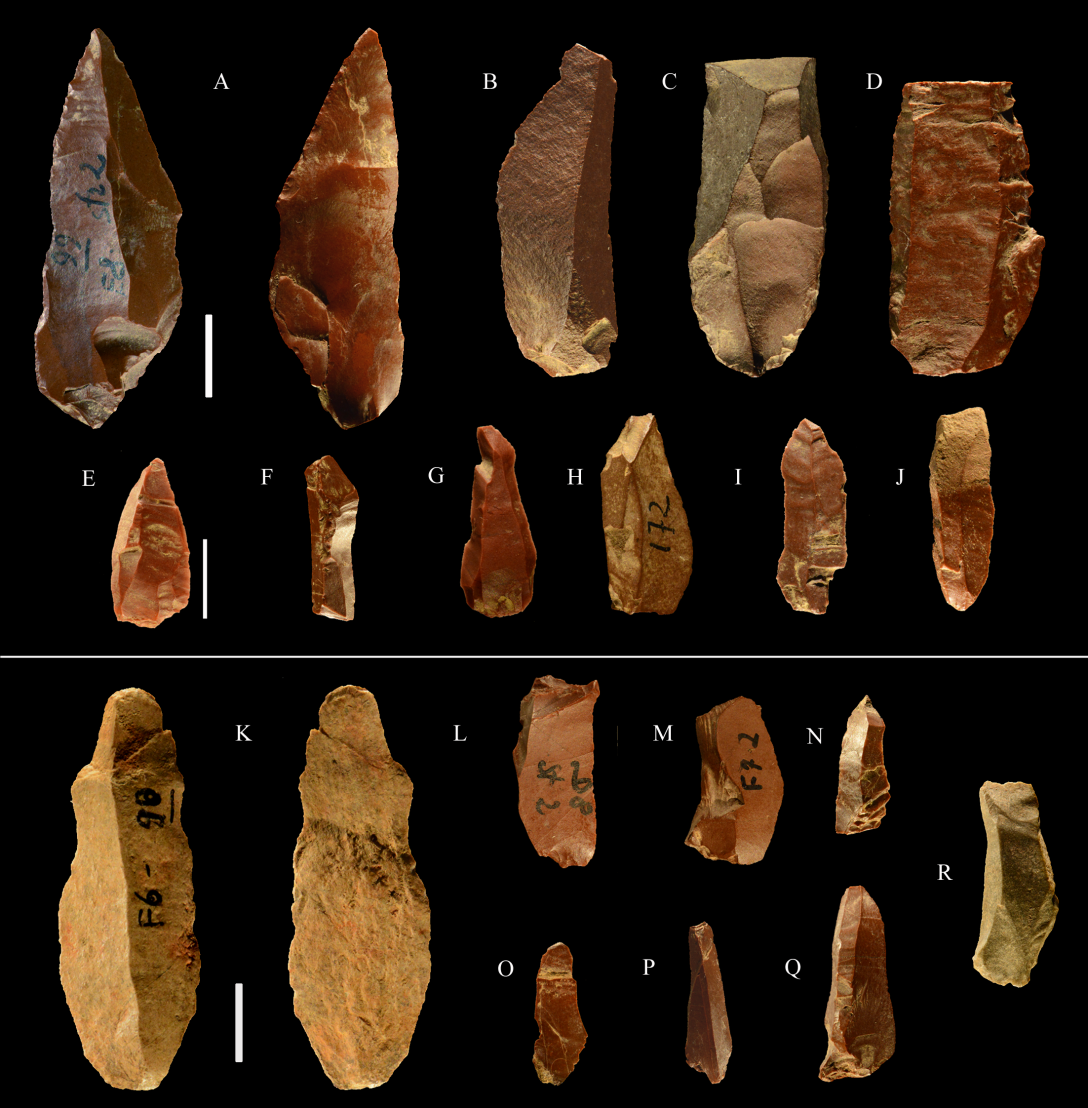
**La Fabbrica, Uluzzian blades and bladelets, made by direct percussion (top) and by the bipolar technique (bottom).** (A-D) blades. (A) has distal marginal retouch. (E-J) bladelets. There are no common dorsal patterns to these blades and bladelets, suggesting an occasional production. (K) bipolar blade. (L-R) bipolar bladelets. The irregular shape of bipolar bladelets is not surprising since bipolar reduction has very little control on the shape of the products (see SI Appendix, p. 20 in [35]). Scale bar = 1 cm.

**Debitage** (Table 9 and Fig 13)

**Table 9:** La Fabbrica Uluzzian. Counts of flakes and blades.

Debitage, all raw materials (quartz specimens in parenthesis)	N	%
Flakes from non-Levallois cores, with unidirectional, bidirectional, orthogonal or convergent dorsal scars	95	23.5
Pseudo-Levallois points and débordant flakes	11	2.7
Ordinary cortical flakes	40 (3)	9.9
Ordinary partly cortical flakes	73 (30)	18.0
Ordinary non-cortical flakes	58 (19)	14.3
Bipolar flakes	40 (24)	9.9
Flat flakes (see S3 File for description)	11	2.7
Blades^a^	13	3.2
Bladelets^b^	30 (6)	7.4
Bipolar blades	5	1.2
Bipolar bladelets	12 (3)	3.0
Flakes from scaled pieces	16	4.0
Undetermined flakes	1	0.2
Total	405	100.0

^a, b^ Ten blades and three bladelets have some marginal partial retouch, bipolar blades and bladelets have no retouch at all.

**Fig 14:**
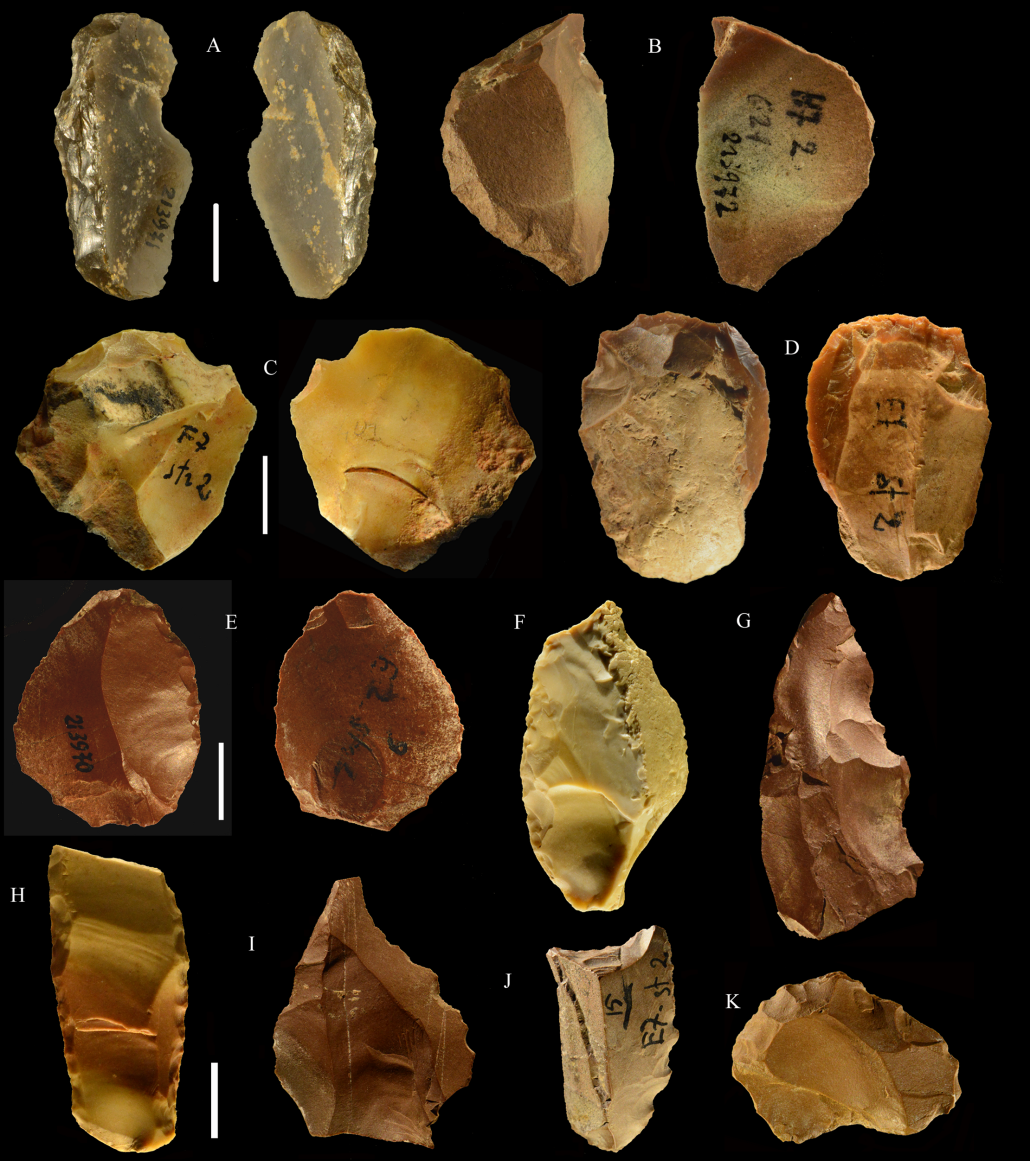
La Fabbrica. **Uluzzian retouched pieces of flint (A,C, F, H) and jasper (B, D, E, G, I-K).** (A) backed piece (lunate) on a laminar flake, the platform was removed. The backing retouch is both direct and inverse. The notch on the right side is not intentional, it is a natural fracture due to fissuring in the flint. (B) backed piece (lunate) on a flake with little retouch on the distal point, the platform was removed. (C) end scraper on flake with traces of organic residue on one side and thermal scar. (D) end scraper on a bipolar core. (E) endscraper on unidirectional flake. (F) side scraper on a unidirectional flake with a cortical side. (G) denticulate on the ventral face of a flake. (H) double scraper on a blade. (I) denticulate on a flake with convergent dorsal scars from a non-Levallois core. (J) Concave truncation and partial denticulate on the right side of a blade. (K) endscraper on flake. Catalogue numbers: 3, 5, 6, 32, 8, 14, 82, 80, 19, 18, 10. Scale bar = 1 cm.

**Small tools** (Tables 10 and 11, Fig 14)

**Table 10:** La Fabbrica, Uluzzian. Counts of small tools.

Type	All raw material except quartz	Quartz	Total	%
Scaled pieces	47	3	50	44.2
Lunates	2	0	2	1.8
End scrapers	4	0	4	3.5
Side, transverse and convergent scrapers	10	0	10	8.8
Denticulates and notches	14	1	15	13.3
Truncations	1	0	1	0.9
Bec	0	1	1	0.9
Retouched and utilized blades and flakes	19	9	28	24.8
Tool fragments	2	0	2	1.8
Total	99	14	113	100.0

**Table 11:** La Fabbrica, Uluzzian. Blanks of small tools.

Categories^a^	All raw materials except quartz	Quartz	N	%
Flakes, flake fragments	57	7	64	71.1
Bipolar flakes	4	2	6	6.7
Blades	7	1	8	8.9
Chunks, 1 bipolar core	7	1	8	8.9
Pebbles, rolled blocks	4	0	4	4.4
Total	79	11	90	100.0

^a^Undeterminate cases are excluded. Flat flakes and bipolar blades or bladelets were not selected as tool blanks; bipolar flakes were rarely used.

**Fig 15:**
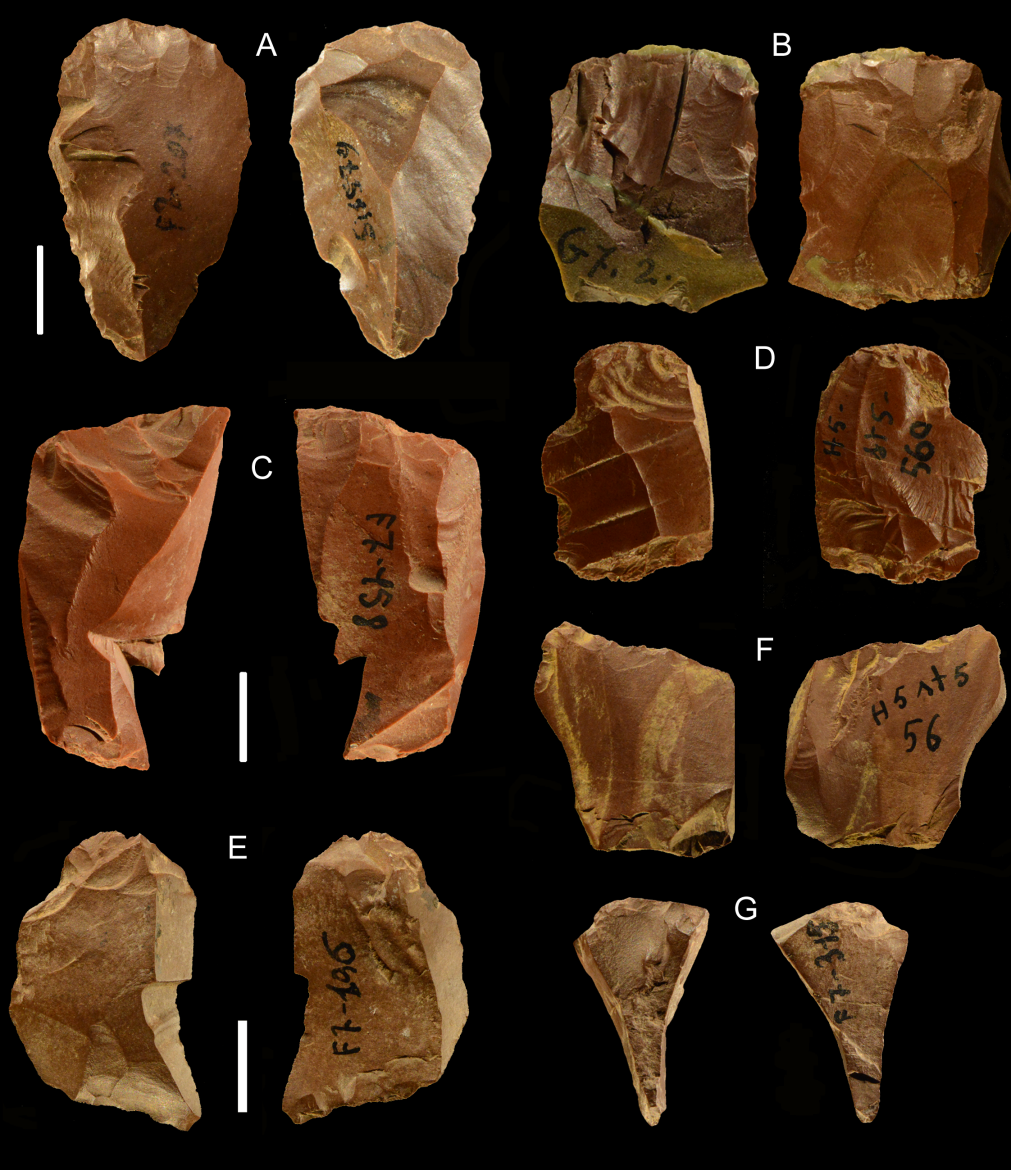
La Fabbrica. **Uluzzian scaled pieces of jasper**. Catalogue numbers: 92, 29, 23, 35, 93, 25, 49. (A) has some retouch on the left side.

**Scaled pieces** (Fig 15). As explained in “Methods” we use the term “scaled pieces” to indicate tools shaped by utilization and as defined in Upper Paleolithic assemblages [29]. A strong increase in the production of scaled pieces is observed in the Uluzzian. Its significance is discussed in the section “Are the Uluzzian innovations unequivocal indicators of a modern human culture?”

**Bone tools and ochre.** Fifteen bone tools have been discovered in Uluzzian layers: 8 at Grotta del Cavallo, 5 at Castelcivita, 1 at Grotta della Cala and 1 at La Fabbrica [36 and references therein]. Of the eight tools at Cavallo, 6 are awls and two lack the proximal and distal end. Of the 5 at Castelcivita [37] four are awls and one is pointed at both ends. The Grotta della Cala and La Fabbrica bone tools are also awls.

**Fig 16:**
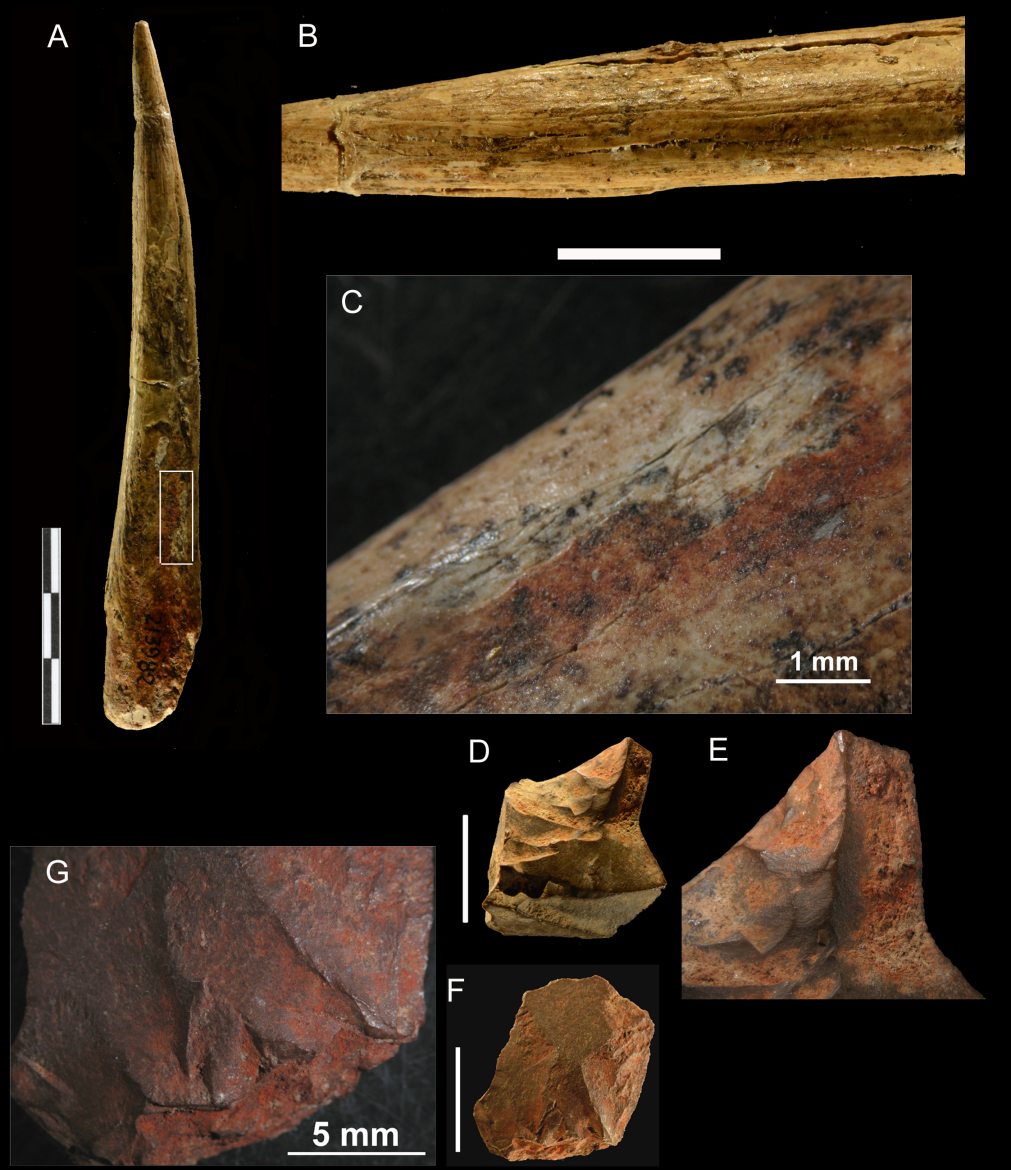
La Fabbrica, layer 2. **Ochre-stained bone awl and small flakes covered with ochre.** (A) Bone awl, 12 cm long, on a horse accessory metapodial. (B) scraping marks on the awl. (C) detail of ochre stained area. Two small flakes covered with ochre (D, F) and details of ochre coating on flakes (E,G). The scale of B, D, F is 1 cm; the scale of A is 3 cm.

The bone awl from la Fabbrica was found in square F7 [10]. It is a horse accessory metapodial. The naturally pointed bone was modified by scraping all along the shaft (Fig 16A–16C). Ochre coating is present at the base of the tool but a few small dots are also present along the shaft.

Ochre staining is present on some of the bone awls from the Still Bay phase of Blombos Cave, dated to 75–72 ka [38, 39]. The Blombos awls were made on bovid long-bone shaft fragments shaped by scraping with a stone tool. Two bone awls with residues of red pigment have been reported from the Châtelperronian of Grotte du Renne [40]. It was suggested that ochre staining came from using the tool on ochre stained hides. It is also possible that the La Fabbrica awl was completely covered by ochre and that most of the ochre was removed through the intensive use of the tool and remained only on its handle as it was protected by a hand.

Three stone flakes, all smaller than 2 cm, were found in squares F6 and G7. The red pigment on the awl and on two of the flakes (Fig 16D–16G) has been analyzed by one of us (CB).

**Analysis of the pigment.** The microscopic observation of the surfaces of the bone awl and the stone flakes reveal the presence of reddish coatings and mineral grains. Whereas the latter, mainly represented by quartz and lithic fragments, are likely related to the cave sediments, the former cannot be attributed to the cave environment where the samples were found.

**Fig 17:**
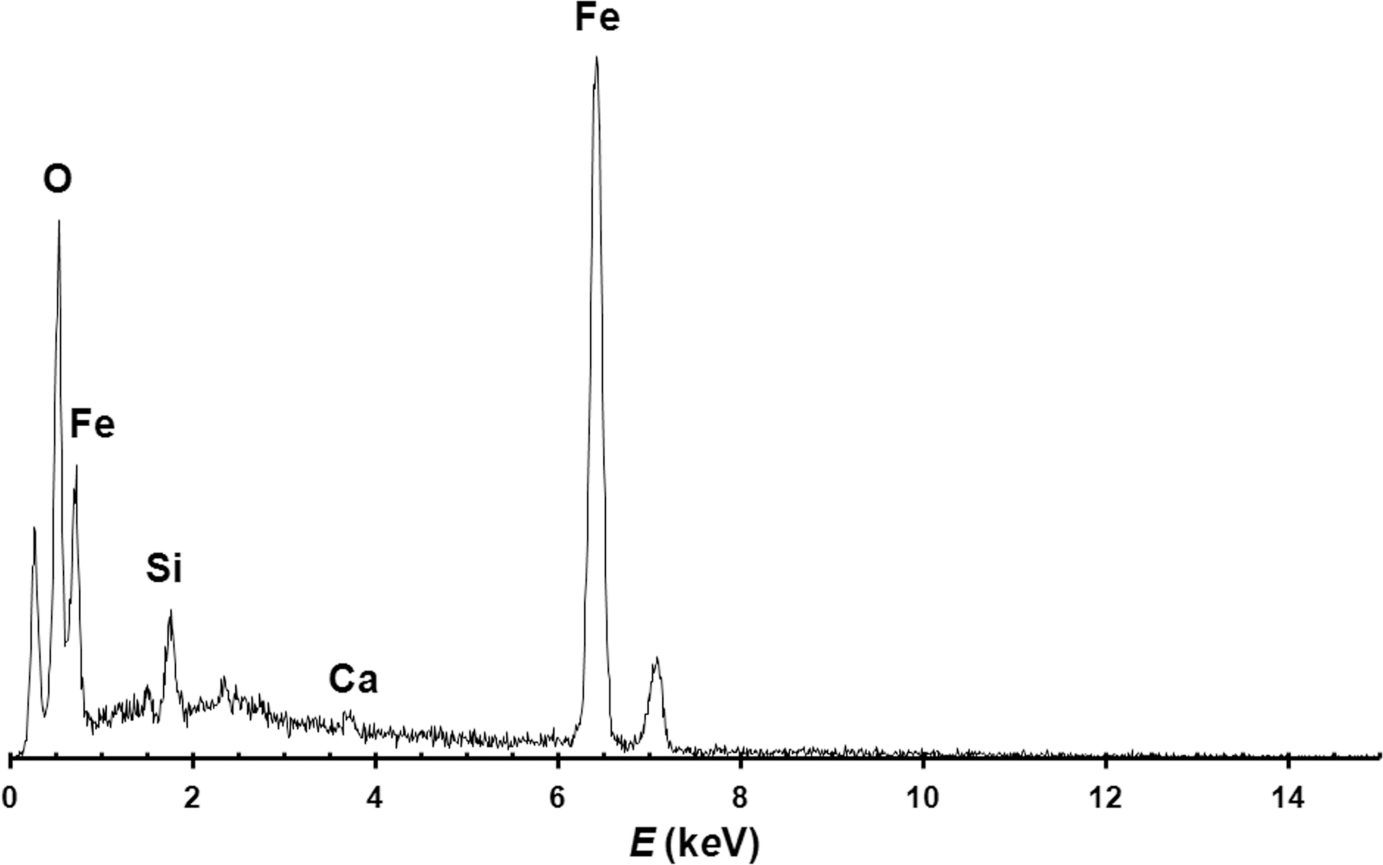
EDS spectrum of the red coating on one of the stone flakes.

The reddish coatings on the bone awl and the stone flakes were studied through a Philips XL30 scanning electron microscope (SEM) equipped with an EDAX DX4 spectrometer for qualitative chemical elemental analysis. Fig 17 shows a representative EDS spectrum of such red coatings, showing its Fe-rich composition, with minor amount of Si and traces of Ca. This composition suggests the occurrence of iron oxides/hydroxides associated with minor quartz and calcium carbonate. Owing to the small amount of available material, X-ray powder diffraction data cannot be collected on the red coatings (but see S4 File).

**Chemical analysis of hafting adhesives.** Two of us (ID and JJL) applied gas chromatography/mass spectrometry (GC/MS) to the characterization of amorphous materials on three samples: two from La Fabbrica and one from Colle Rotondo. The sediment corresponding to the sampled pieces was also analyzed to rule out external contamination. Full analytical details are provided in the S4 File.

**Fig 18:**
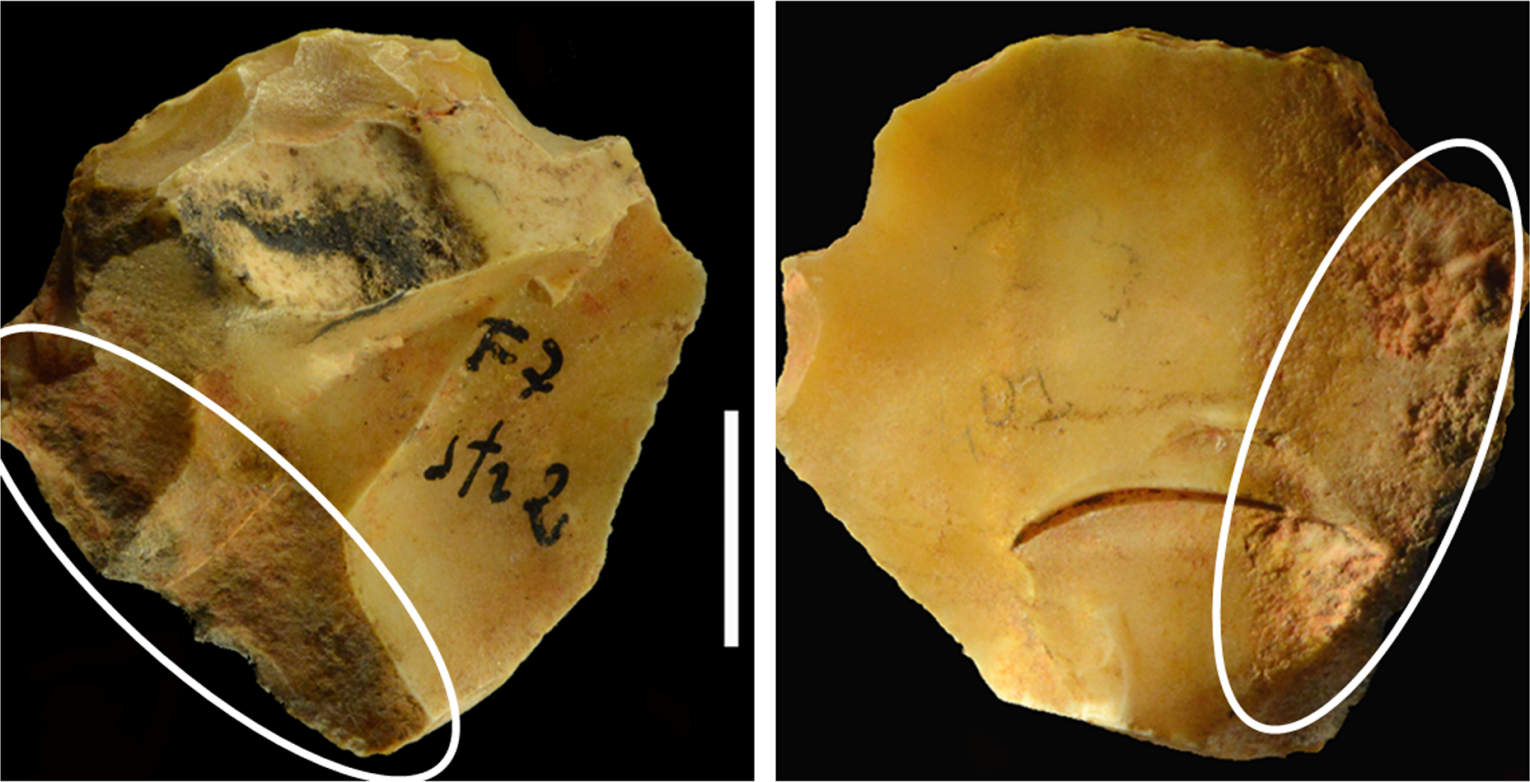
End scraper from layer 2, square F7 with hafting residue and location of sampling for GC/MS (S4 File).

The three samples had clearly visible residues on a lateral edge. They are 1) an unretouched flake from Colle Rotondo, 2) one side scraper from the Mousterian layer 1b of La Fabbrica and 3) one end scraper from the Uluzzian layer of La Fabbrica (Fig 18). Microsamples (less than 0.1 mg) were collected from the specimens and submitted to a combined analytical procedure for the identification of lipids, waxes, and resinous materials [41]. With regard to the sample collected from Colle Rotondo, the chromatographic profile of the sample extract and of the sediment perfectly matched. No hafting material was identified. The profile of Mousterian side-scraper of La Fabbrica differs from that of the sediment in that more peaks were identified in the side scraper than in the sediment (Fig E: a, b in S4 File) but their profile is similar by the absence or by an insignificant amount of diterpenes. Thus no conclusions can be drawn on the presence of hafting adhesives.

The Uluzzian end scraper instead gave significant results (Fig 18). The chromatogram (S4 File) shows the presence of a relevant amount of diterpenes, indicating the use of a conifer resin, and a lipid material. The main components of the diterpenic fraction are dehydroabietic and 7-oxo-dehydroabietic acids, indicating a resin from a plant belonging to the conifer group such as *Pinaceae*. The profile of the lipid components suggests the co-presence of plant and animal fats, due to the occurrence of fatty acids with even and odd carbon number, ranging from C12 (dodecanoic acid) to C25 (pentacosanoic acid) [42].

Hafting adhesives have been documented by chemical analysis such as GC/MS in Middle Paleolithic sites, such as birch bark pitch at the site of Campitello Quarry in Tuscany (Italy) dated to the end of MIS 7 (about 200 ka) and at the German site of Königsaue A with an estimated age of 80,000 years [43]. At Umm el Tlel (Syria) bitumen was used for hafting on tools dated to 71,000 and 40,000 y-old [43]. Ongoing work by two of us (ID and JJL) documents use of resin on Middle Paleolithic tools in Latium. This is however the first time that hafting is documented at an Uluzzian site.

### Colle Rotondo

Colle Rotondo in Latium is a recent discovery proving the presence of the Uluzzian in a region where it was supposed to be completely absent [15–16]. It was excavated in 2011 and 2012 under the direction of Massimo Pennacchioni.

The site is located in southern Latium, 8 km north of Anzio (Fig 2A). It lies on top of a flat hill, about 63 m asl and 2.3 km from the Tyrrhenian sea (Fig 2B). The topsoil consists of dark brown (Munsell Soil Chart 10YR, 3/3) sandy silts heavily disturbed by agricultural practices. The single layer with stone artifacts was found at a depth of 75 cm from the surface and is of variable thickness from 6 to a maximum of 20 cm, with thickness increasing east to west, and overlying a sterile unit. It consists of dark reddish brown silty sands (Munsell Soil Chart 2.5 YR, 2.5/3) with small nodules of iron oxide and/or manganese. The sandy layer was compacted and consolidated with localized evidence (in one square only) of channeling and run-off where artifacts were not lying horizontally but were inclined (Fig 7 in S1 File).

**Fig 19:**
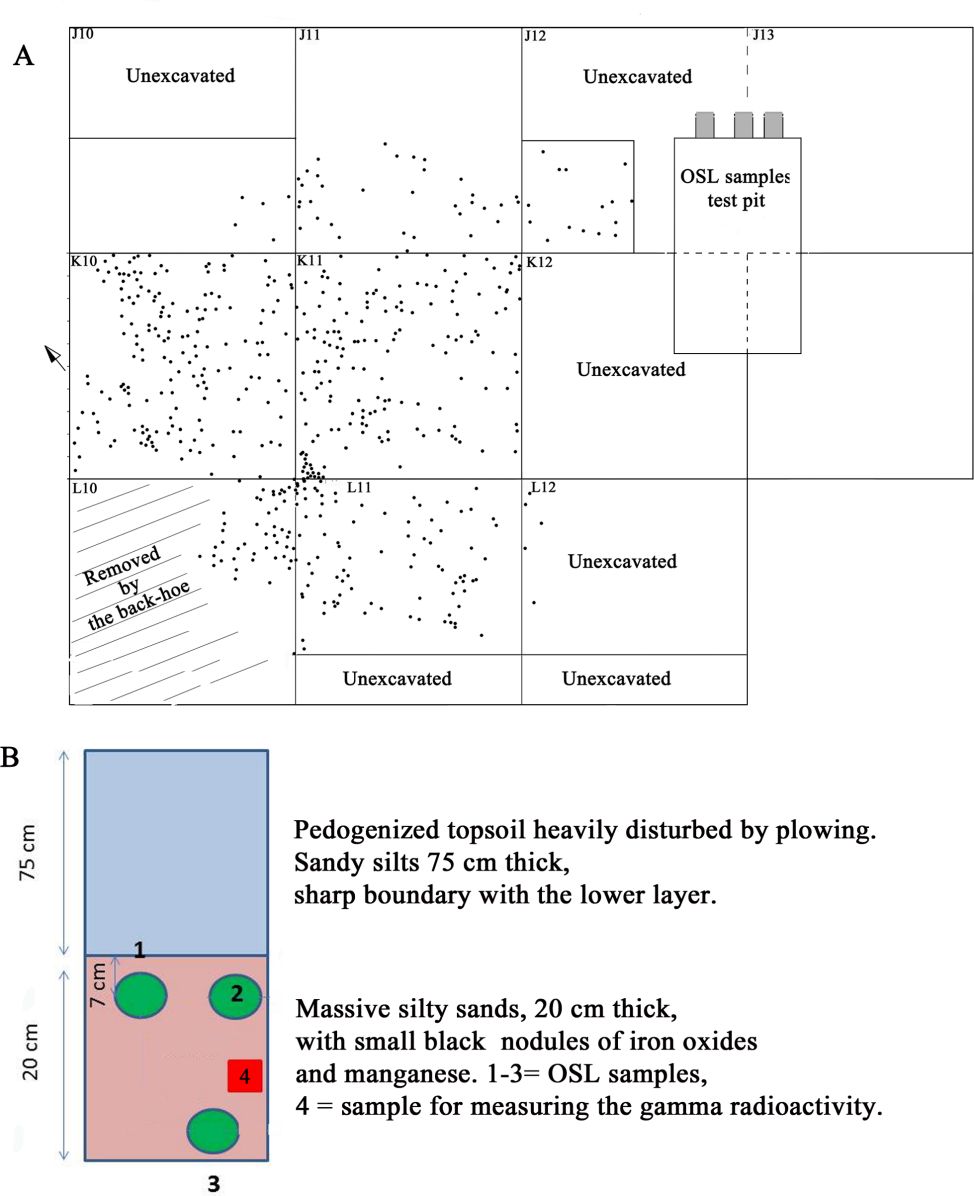
Colle Rotondo plan of the excavation and stratigraphic scheme for OSL sampling. (A) Plan of the excavation with 1 m grid showing all artifacts > 1.2 cm, and location of the test pit for OSL samples. The limits of the artifact distribution were found in squares J10 and J11. In square L10 the deposit was damaged by the back-hoe used to remove the topsoil; elsewhere it was unexcavated. (B) Stratigraphic scheme indicating the position of OSL samples CR 1–3. Sample CR 4 was not used for OSL dating since the radioactivity was measured directly on samples 1–3. Drawing by G. Zanchetta.

The area excavated in 2011 and 2012 by one of us (Massimo Pennacchioni) is about 6 sq m. (Fig 19A). The excavation proceeded by levels 1–2 cm thick but refitting between elements of different levels (Figure B: L+M in S1 File) indicate that some elements had been vertically displaced; thus the five excavation levels have been treated as a single unit. All the sediment was dry-screened with 2 mm mesh. OSL samples for dating were collected by G. Zanchetta (Fig 19B) and analyzed in the laboratory of the University of Milano-Bicocca (S2 File). The OSL dating results are discussed in the section on “Geomorphology”.

#### Taphonomy

More than 80% of all artifacts come from the first three excavation levels (including flakes <1 cm, retouched pieces and cores that have a maximum length of 6 and 7 cm). The lowest two excavation levels (levels 4–5) were found only in one square. The most common raw material is flint.

On a random sample of 100 artifacts, 23% are slightly patinated, often covered with reddish-brown concretions and black manganese stains. Patination, a form of weathering where sunlight and water play a certain role [44] might suggest that some artifacts remained exposed to the air for a while. However all artifacts are fresh with sharp edges. Only 28% have a few minute microfractures (abrupt scars ≤ 1 mm), none have macrofractures or scars without bulb of percussion >2 mm.

There were very few badly altered bone fragments and no charcoal. One small Levallois core (with a preferential flake scar) and one small Levallois flakes were found in square K10 and L10 respectively (Fig E in S1 File). The core shows water polish and numerous microscars on the edges [45–46] two features that are not present in the rest of the assemblage, suggesting that the piece is redeposited or was collected by the Uluzzians. The small flake is slightly polished. We do not consider them as part of the assemblage. Mousterian artifacts were found in the east area of the hill mixed with protohistoric materials in a rampart built during the Bronze Age [47].

#### Stratigraphy and micromorphological analyses

**Fig 20:**
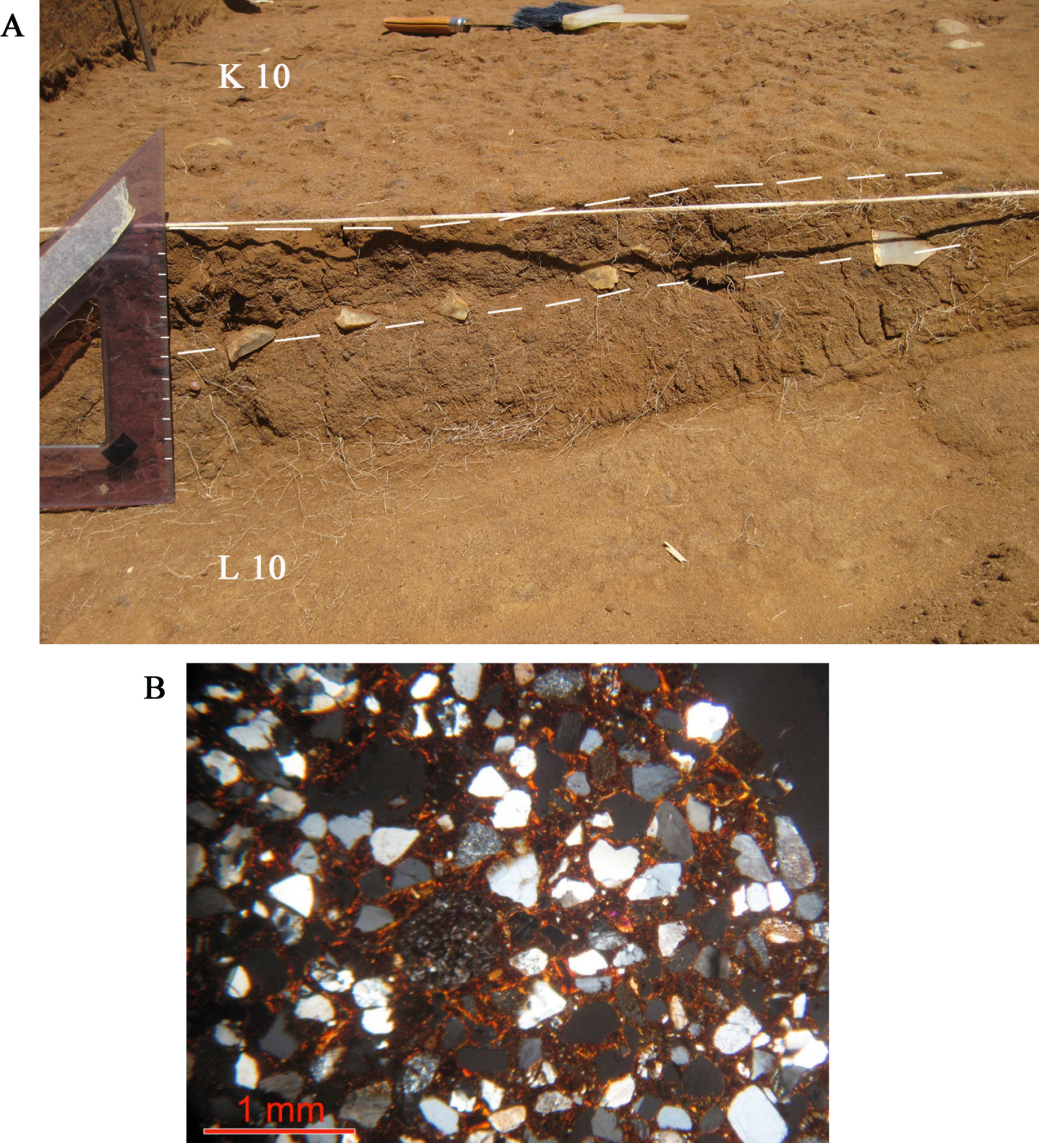
Colle Rotondo excavated layer and thin section. (A) This excavation photo shows a section between squares L10 and K10 as uncovered by the backhoe. The triangle rests on the ground open by the backhoe. Here the level with the artifacts, marked by dashed lines is 6 cm thick while in the OSL test pit to the east it is about 20 cm thick. The flat surface at the top shows the beginning of the excavation in square K10. (B) Thin section of sample CR1. Thin clay coatings around skeletal grains and stipple speckled b-fabric. Both characteristics point to an incipient level of reorganization of the groundmass and therefore to a weak pedogenesis. Cross-polarized light (XPL). The thin section of sample CR1 was manufactured according to methods of [48] and described by using the terminology of [49]. Microphoto by C. Nicosia.

The stratigraphic features of the terrains exposed by archaeological excavation at Colle Rotondo are shown in Figs 19B and 20A. A surface pedogenized horizon constituted by brown sandy silt, heavily disturbed by plowing, occurs in the upper 75 cm. It overlies a reddish-brown silty sand horizon, incorporating mm-sized Mg and/or Fe concretions. The lithic industry concentrates in the top 6–20 cm thick portion of this layer (Fig 20A).

The thin section of sample CR1 (Fig 20B) shows that the sand fraction is composed of a mixture of silicoclastic and volcaniclastic (i.e. pyroxenes–augite) mineral species. The moderate sorting, the granulometry, centered on the medium sand granulometric class, and the slight rounding of the grains are compatible with an aeolian sediment. There are no traces of reworking due to surface runoff or similar water and gravity-triggered slope processes. Pedogenesis is at an initial stage, according to the incipient reorganization of the groundmass, giving rise to a stipple-speckled b-fabric. Weak traces of incipient clay illuviation, such as thin clay coatings around skeletal grains, also point to an initial/moderate level of pedogenesis. The weatherable minerals in the coarse fraction–especially pyroxenes–are abundant and do not yet show traces of weathering.

#### Geomorphology

The Colle Rotondo site is located on the flat surface of an E-W elongated hill, bordered by the steep flanks of two convergent streams (Fig 2B). The canyon-like morphology in this area is the result of the intensive erosion of a suite of ancient paleo-surfaces, due to the interplay between a regional uplift of ca. 50 m occurred since 250 ka and the intervening glacio-eustatic fluctuations [50].

The relic paleo-surface of Colle Rotondo was mapped by Marra et al. [50] who recognized a suite of terraces along the Tyrrhenian coast of Latium between Civitavecchia and Anzio.

Geochronological constraint provided by the 40Ar/39Ar age of 129±1 ka yielded by a tephra layer intercalated within the sedimentary succession forming the coastal terrace at ca. 36 m a.s.l. allowed Marra et al. [51] to correlate the corresponding paleo-surface with the MIS 5.5 terrace previously recognized in this area [52–54]. By combining this datum with previously achieved geochronological constraints on the MIS 7 aggradational succession (Vitinia Formation) [52, 55–56] a well preserved paleo-surface at 52–56 m was correlated with the MIS 7 terrace, and the entire suite of three underlying terraces was correlated with MIS 5.5, MIS 5.3, and MIS 5.1. Moreover, a less preserved terrace ranging 61–65 m a.s.l., including the Colle Rotondo paleo-surface, was tentatively correlated with MIS 9 by [50]. Studies in progress have now recognized a higher paleo-surface at 75–79 m a.s.l. as the MIS 9 terrace, whereas the underlying terrace has been correlated with MIS 7.5 and that at 52–56 m with MIS 7.3–7.1 (Fig H in S1 File).

Therefore, the Colle Rotondo paleo-surface originated circa 240 ka (age of the MIS 7.5 highstand) above the sandy deposits of an alluvial to back-beach coastal plain, and since then experienced repeated cycles of very limited erosion (during the sea-level lowstands) and eolian/colluvial deposition (mainly during the highstands). In contrast, the present day deep fluvial incisions progressively developed since the MIS 6 lowstand, and cut deeper and deeper the original morphology due to the intervening uplift that raised the coastal area for an amount of 50 m, during the last 200 ka.

Since about 60,000 years ago, corresponding to a time when the sea-level fell down to -100 m [57] the Colle Rotondo plateau has remained completely isolated with respect to the surrounding hills, and no fluvial sedimentation, nor significant colluvial deposition could affect this area. At the same time, phenomena of removal and transportation of the terrains forming the plateau surface have affected only very fine grained material (sand or finer), since the erosional agents were limited to wind and surface water runoff.

This fact implies that the lithic material was brought in situ by man after 60,000, or that it represents relic material which was deposited on the surface after its formation 240 ka, and that it experienced only limited colluvial/runoff transport. In fact, the artifacts, with diameter ranging from 2 mm to 9 cm, are incorporated within a 6 to 20 cm thick silty sand layer (bounded by the dashed lines in Fig 20A) without displaying size gradation, and are characterized by fresh, sharp edges. This feature, combined with flat morphology that characterized the surrounding area since the time of its formation around 240 ka, excludes that the coarse material was fluvially transported. After the artifacts were made and abandoned on the Colle Rotondo hill by man, the material did not experience even limited transport, (e.g., colluvial/runoff) according to the micromorphological analyses on the sediment that contains it, and consistent with the sub-horizontal, isolated morphology of the hilltop.

Moreover, the pristine nature of the sedimentary layer, showing no traces of reworking, allows us to exclude that the lithic material is a relic component, incorporated within a younger matrix.

#### OSL dates

OSL dating performed on three samples collected at the base (CR3) and at the top (CR2, CR1) of the layer incorporating the artifacts yielded unexpected young ages ranging from 18,180 ± 950 for CR3, 15,870 ± 1,100 for CR 2 and 14,640 ±960 for CR1 (Table A in S2 File). At the time indicated by the OSL dates there are in Italy only Epigravettian assemblages which are dominated by blades, bladelets and microliths and are completely different from the Colle Rotondo assemblage [58]. The geomorphologic and micromorphological analyses show that this is an eolian deposit with no traces of reworking due to runoff or slope processes and only a moderate level of pedogenesis. The presence of manganese stains on some of the artifacts is compatible with this observation. However the slight patina occurring on 23% of the artifacts strongly suggest that some artifacts remained exposed or were re-exposed to the air. Since the deposit is eolian and unconsolidated the quartz grains originally sealed from daylight must have been re-exposed by wind and a total or partial bleaching of the luminescence signal accumulated since deposition may have occurred.

The OSL dates testify that a relatively fast accumulation of ca. 75 cm of eolian sand occurred after the Last Glacial Maximum (ca. 18 ka) since the Last Glacial termination (ca. 14 ka). This picture is consistent with the overall climatic condition during the last 60 ka, characterized by cold and arid conditions during all the full glacial period 60 through 18 ka, favoring eluviation processes rather than eolian deposition and soil formation, followed by warm and humid condition in the Holocene, accounting for the rapid accumulation of an eolian soil above the former ground surface.

The lithic assemblage, unaffected by eolian transportation, is likely to have remained close to the exposed surface until around 14 ka, and then rapidly buried: a fact that fully justifies the OSL dates yielded by the sediment immediately below the artifacts (CR 3 sample, 18 ka) and immediately above them (CR2 and CR 1 samples, 15–14 ka).

Thus the quoted OSL ages do not refer to the depositional event but to a later sun exposure: specifically the last time of exposure before rapid burial of the archaeological layer.

The assemblage remains undated but the similarity to the La Fabbrica assemblage (see section “Discussion”) suggests that they may be broadly contemporaneous.

#### Lithic analysis of Colle Rotondo

Table 12 shows the classification of the assemblage into major artifact categories.

**Table 12:** Colle Rotondo. Assemblage composition.

Classes of artifacts	N	%
Debitage	490	74.5
Retouched pieces	95	14.4
Cores and core fragments	73	11.1
	658	100.0
Hammerstone	1	
Chunks, flakes < 2 cm, flake fragments, tested and broken cobbles, unclassified Siret breaks, flakes with thermal scars, whole cobbles	970	

**Raw material.** The majority of artifacts (Table 13) are on rounded flint pebbles and cobbles, a minority are also of chert, that is microcrystalline silica like flint but we distinguish them because chert has a coarser and opaque texture, while flint has a homogeneous glossy texture with smooth surfaces (see section on La Fabbrica Mousterian raw material). Well-rounded pebbles and cobbles less than 10 cm in size are reported as interstratified in the hill of Colle Rotondo.

**Table 13:** Colle Rotondo. Counts of raw material by main artifact classes^a^.

Raw material	Debitage		Retouched pieces		Cores and core fragments	
	N	%	N	%	N	%
Flint	325	66.3	86	91.5	64	87.7
Chert	165	33.7	8	8.5	9	12.3
Total	490	100.0	94	100.0	73	100.0

^a^ One indeterminate case is excluded

**Fig 21:**
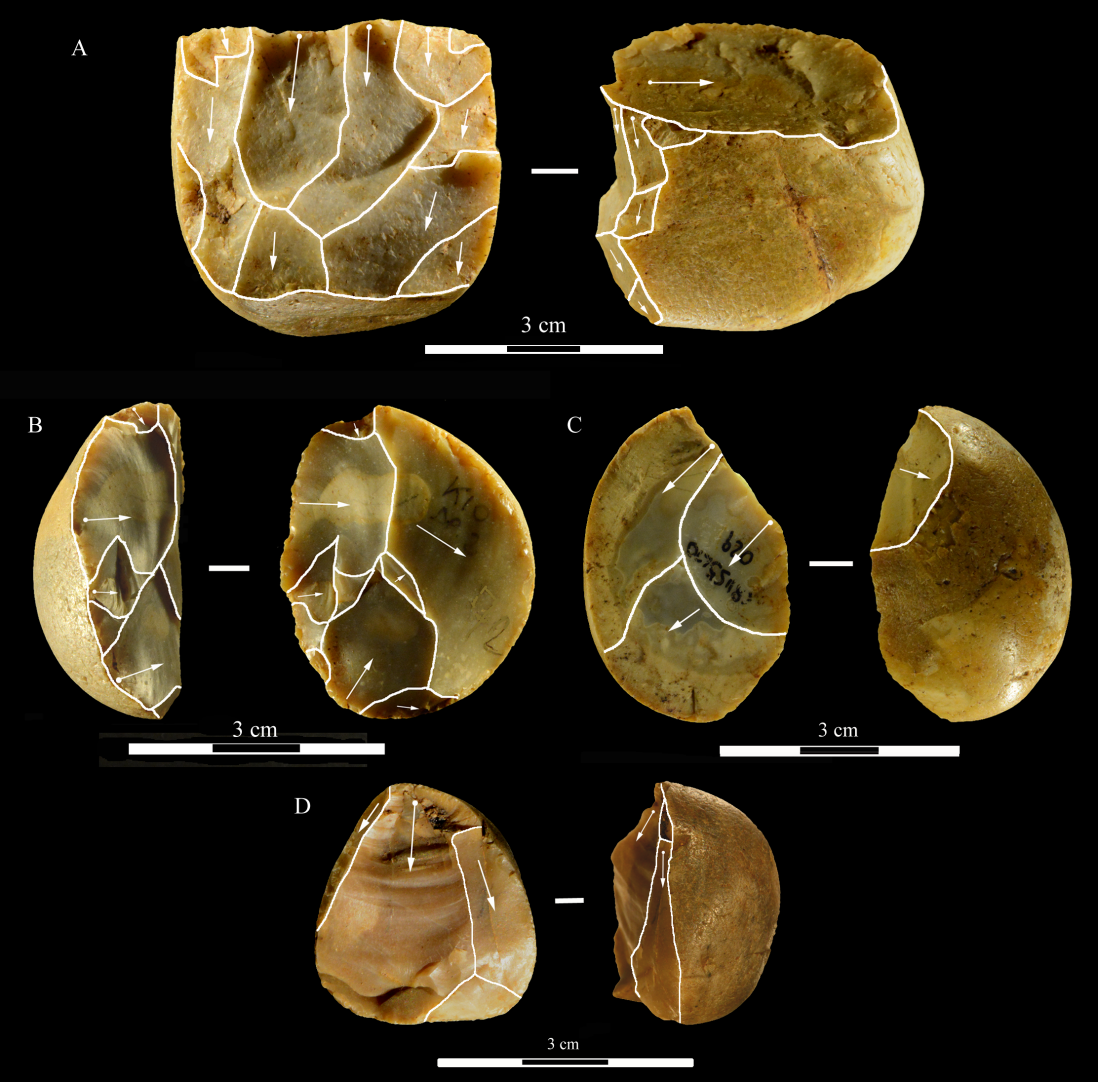
Colle Rotondo cores. Unidirectional cores with series of parallel removals on flint (B-D) or chert pebbles (A). The platform is cortical or a large scar. L of A-D: 44, 34, 36, 27 mm. Catalogue numbers: K10-1.63, K10-4.72, L10-1.29, K10-2.76.

**Fig 22:**
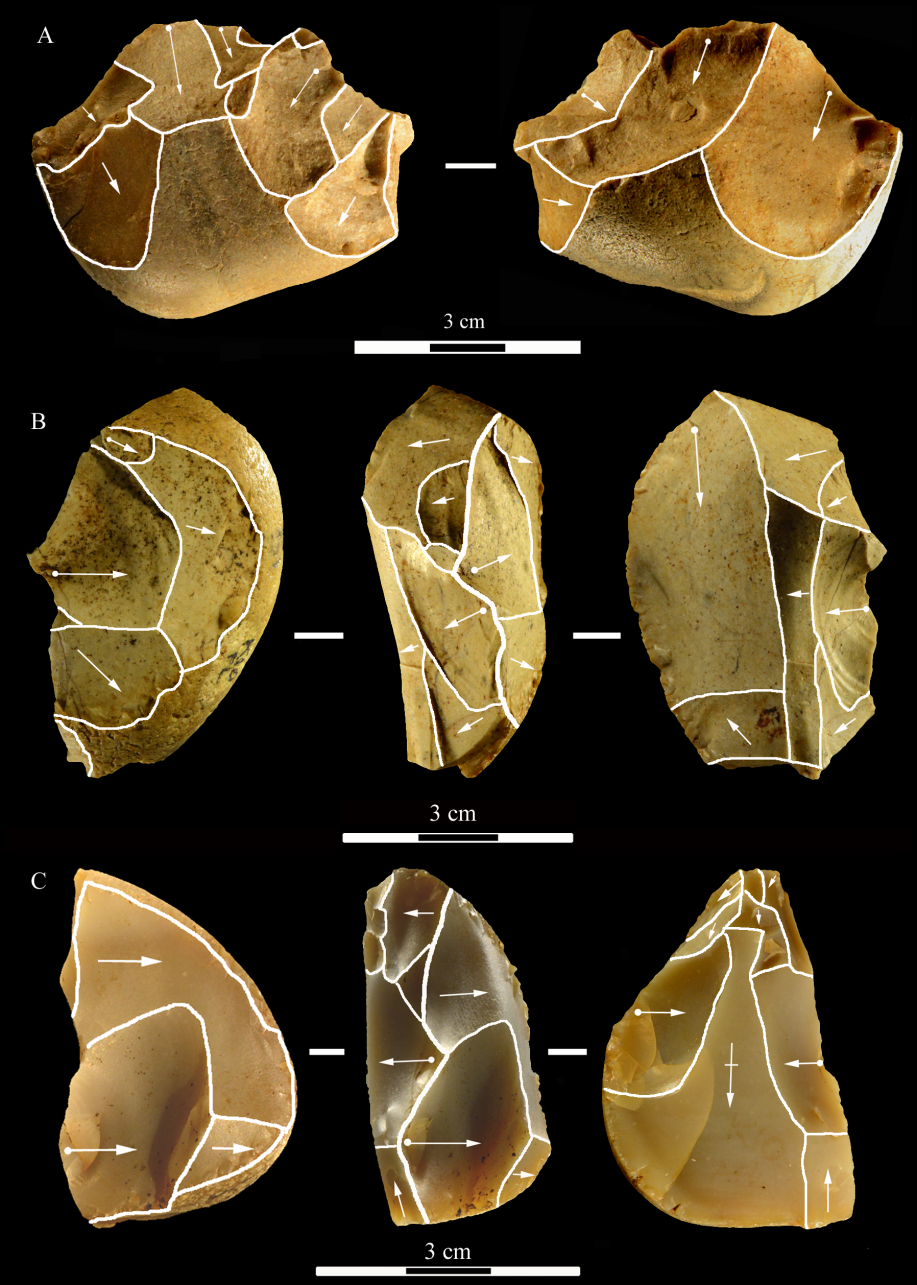
Colle Rotondo cores. Bifacial cores, with alternating removals on opposite faces, on flint (A, C) or chert (B) pebbles. (B) and (C) have additional non-bifacial removals. The last one on B (back view) is a large flake removal from a cortical platform. (C) is on a flake blank, the ventral face of the flake is clearly indicated on the back view; one of the last removals is again a flake from a cortical platform. L of A-C: 48, 50, 38 mm. Catalogue numbers: K11.22, K10-2.15, K10-3.20.

**Fig 23:**
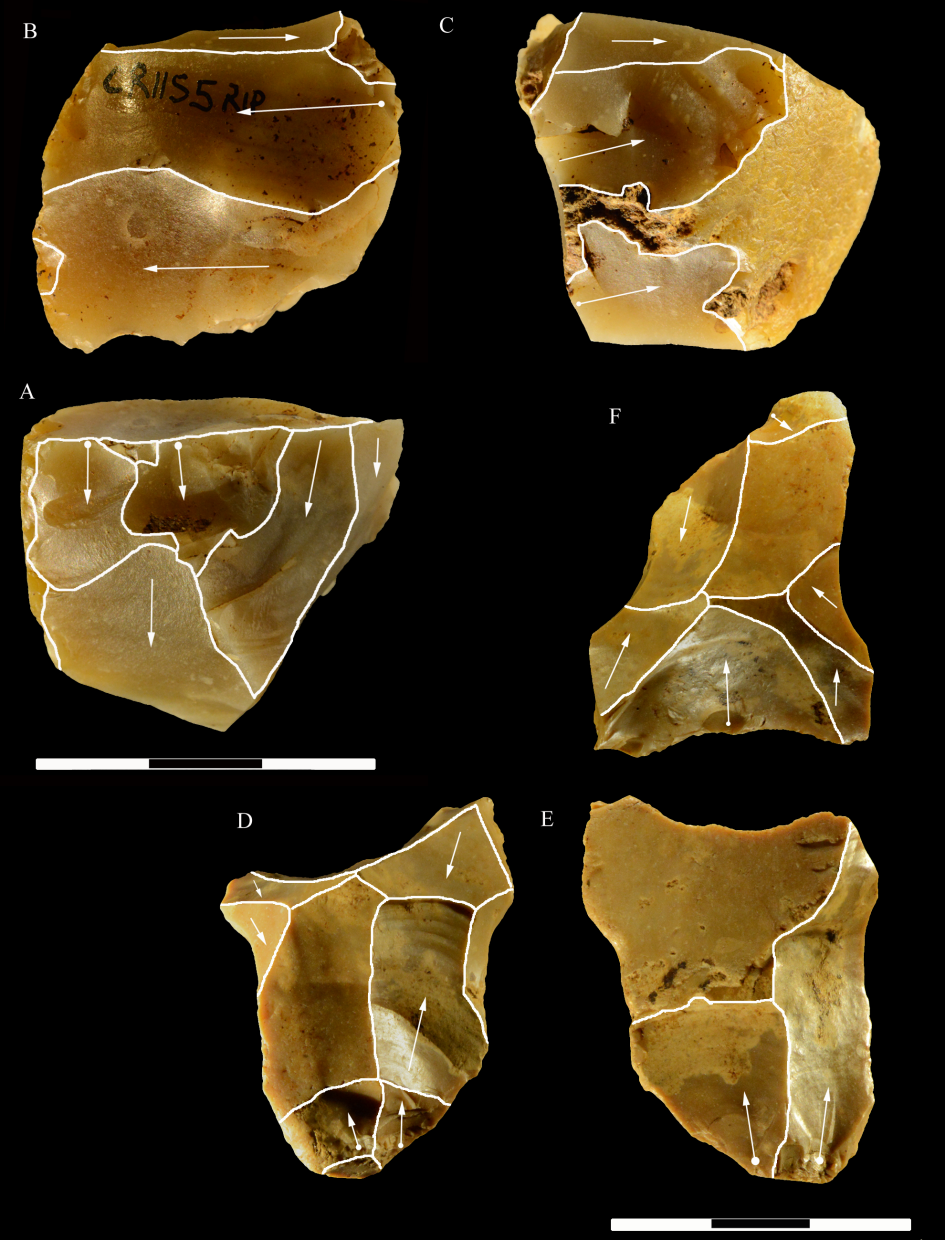
Colle Rotondo cores. (A-B-C) Front, top and back views of a multidirectional core with three debitage surfaces, on a flint pebble. The platforms are previous scars. (D-E-F) Front, back and top view of a multidirectional core on a flint block. The surfaces without an arrow are natural surfaces. The platforms are cortical or natural surfaces. L: 34, 36 mm. Catalogue numbers: L10-L11-1.no, and 1.no B.

**Fig 24:**
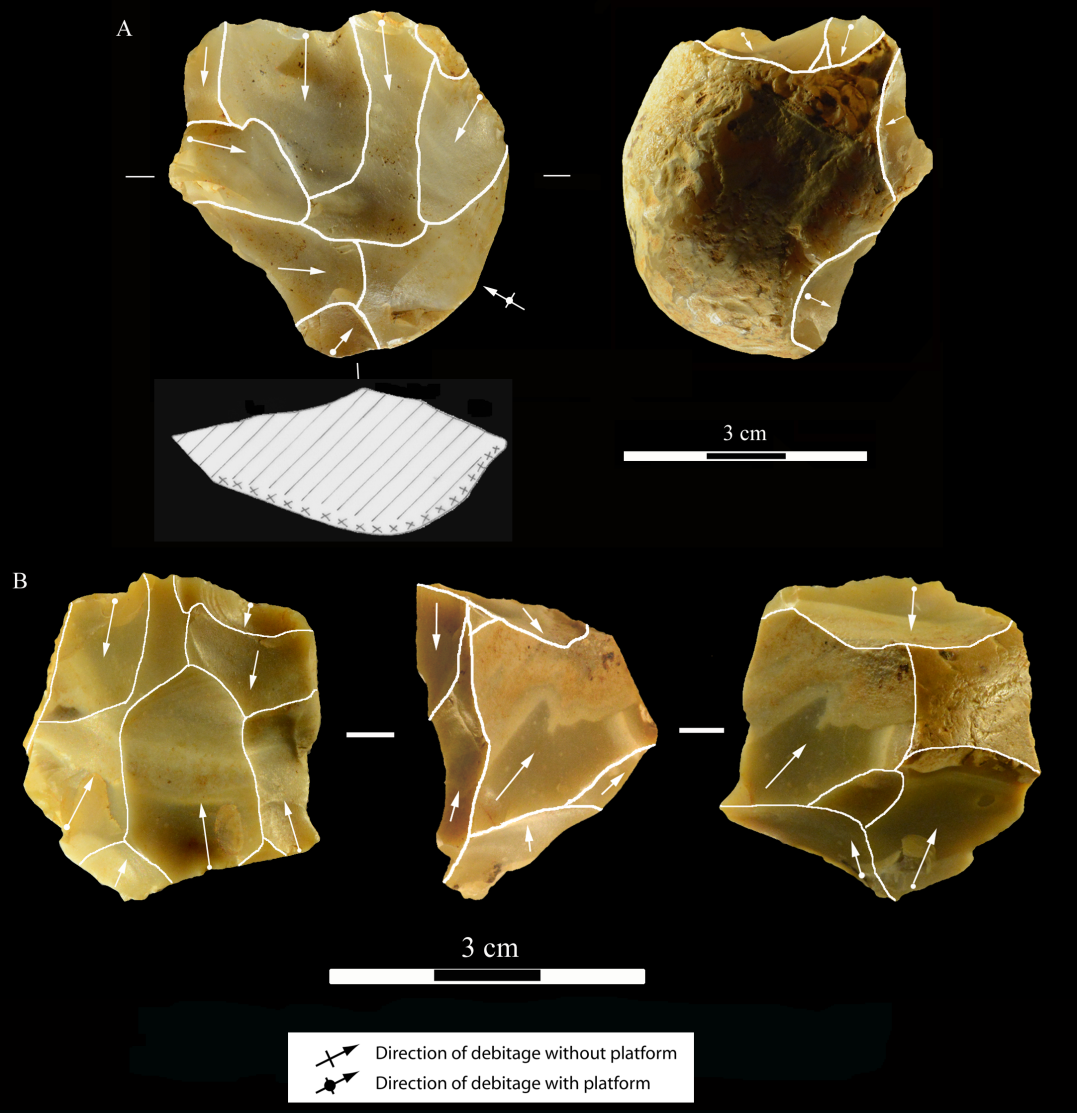
Colle Rotondo cores on flint pebbles. (A) Bidirectional passing to centripetal, single debitage surface and partial platform preparation, on a split cobble. (B) Bidirectional core with two debitage surfaces, front, right profile and back views. The platforms are previous scars. L of A-B: 45, 30 mm. Catalogue numbers: K11.11, and 1.no A.

**Core types.** As in the Uluzzian of La Fabbrica there are no Levallois or discoid cores. The most common cores are unidirectional, bidirectional or multidirectional cores with parallel removals on one or more debitage surfaces and a cortical or natural surface platform or a platform formed by a single large scar or multiple previous scars (Table 14, Figs 21–24). One bidirectional core on flake (Fig 24A) is passing to centripetal with partial platform preparation but it does not have any scars that would indicate preparation of the debitage surface or maintenance of convexities and cannot be classed as Levallois.

**Table 14:** Colle Rotondo. Counts of cores.

Core types	N	%
Core with unidirectional or bidirectional removals on a single or on adjacent debitage surfaces	26	35.6
Cores with multidirectional removals on three or more debitage surfaces	9	12.3
Bifacial core	8	11.0
Core bearing a single removal with non-conchoidal fracture	1	1.4
Bipolar cores and fragments	10	13.7
Unclassified core fragments	15	20.5
Core of undetermined type	4	5.5
Total	73	

**Fig 25:**
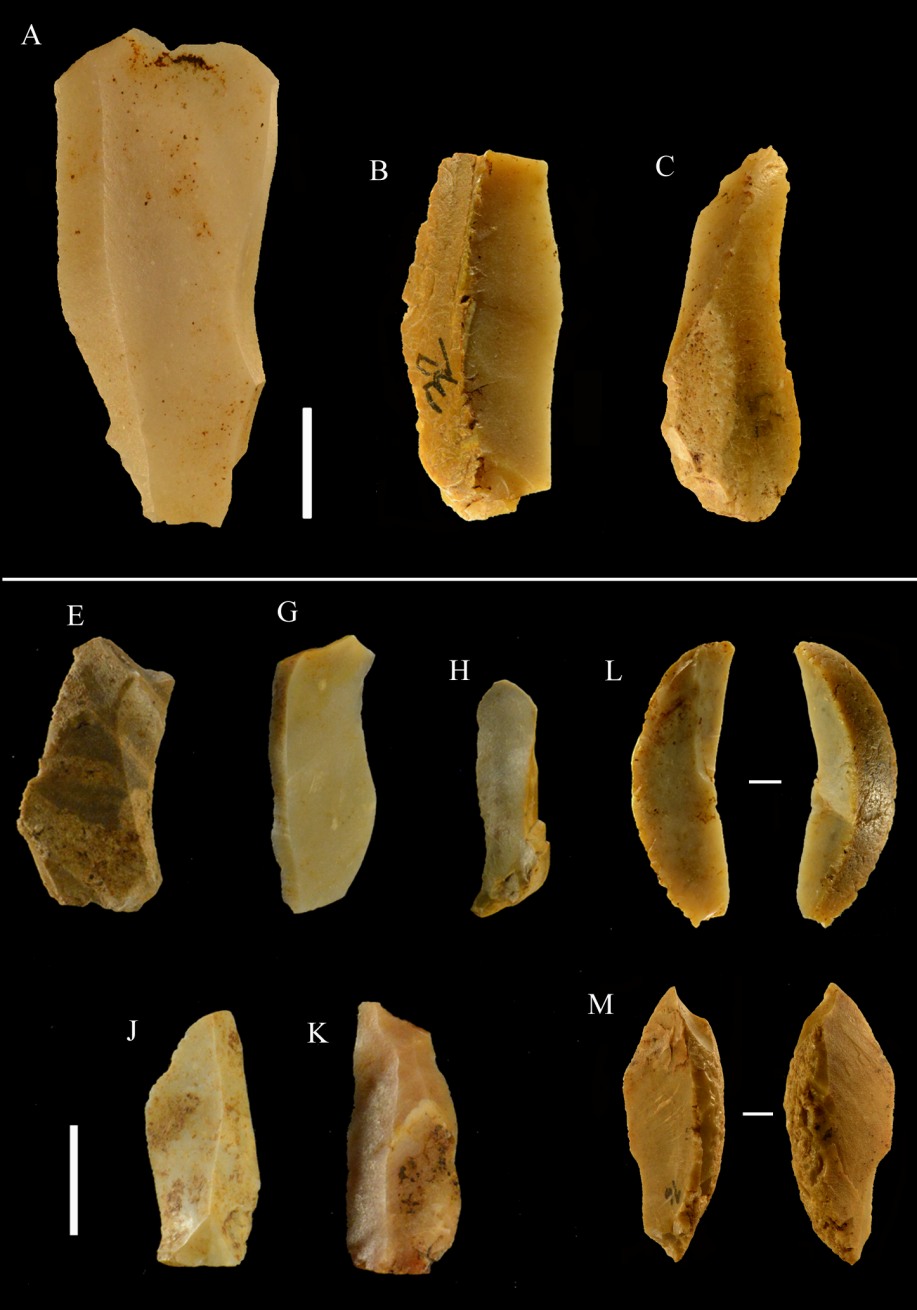
**Colle Rotondo, blades (A-C) and bladelets (E-M).** A to K are by direct percussion; L and M are by bipolar percussion. Length of A-C: 45, 34 (broken distal), 34 mm; Length of E-M: 24, 29, 21, 23, 24, 25, 26 mm. Catalogue numbers; K10.3, K11.3, K11.3, J1.1, J11.1, J11.1, J10 L11.1, J10 L11.1, K11.3, K10.3. Scale bar = 1 cm.

Relatively common are bifacial cores (Fig 22) with alternating removals on opposed surfaces and bipolar cores (Fig D: A in S1 File) some of which have produced some bipolar flakes and bladelets (Fig 25L and 25M). The lack of dedicated blade and bladelet cores suggest a non-systematic approach to their production. Bifacial cores are relatively common while at La Fabbrica there are only two cores which have a similar scar morphology (Fig 9B and 9C). Bipolar cores and flakes are less abundant than at La Fabbrica. This is probably due to the fact that the raw material were well rounded flint pebbles somewhat larger than larger than those of Middle Paleolithic sites on coastal Latium and that could be knapped by freehand percussion.

**Debitage of Colle Rotondo and comparisons with La Fabbrica.** Flat flakes (S3 File) are rare at Colle Rotondo, even rarer at La Fabbrica (Tables 9 and 15). Cores with removals bearing non-conchoidal fractures are also quite rare (absent at La Fabbrica and only 1 at Colle Rotondo). This is to be expected since the technique is often used on small pebbles to initiate a knapping sequence and flat flakes are fully cortical in the great majority of cases (17 of 19 at Colle Rotondo). The mode of production of flat flakes, which consists in striking the pebble resting on a soft anvil (e.g. wood or limestone) or on the ground, is typical of the Upper Pleistocene Mousterian of Latium, called “Pontinian” [59] but is not exclusive of that technocomplex because it is present, although quite rare, in older assemblages, such as the Torre in Pietra level *d* dated to MIS 7 (see Tables 12 and 13 in [60]). Its presence at Colle Rotondo is not really evidence of continuity with the regional Mousterian. As at La Fabbrica the most common reduction sequence is represented by unprepared cores of variable morphology and more than 65% of partly cortical or cortical flakes indicating short reduction sequences.

**Table 15:** Colle Rotondo. Counts of flakes and blades.

Debitage	N	%
Flakes from non-Levallois cores, with unidirectional, bidirectional, orthogonal or convergent dorsal scars	186	38.0
Pseudo-Levallois points and *débordant* flakes	7	1.4
Flakes with centripetal scars but non-Levallois	8	1.6
Ordinary cortical flakes	75	15.3
Ordinary partly cortical flakes	58	11.8
Ordinary non cortical flakes	37	7.6
Bipolar flakes	22	4.5
Flat flakes (see S3 File for description)	19	3.9
Blades	5	1.0
Bladelets	14	2.9
Bipolar blades	3	0.6
Bipolar bladelets	6	1.2
Flakes from scaled pieces	24	4.9
Kombewa	6	1.2
Flakes from tool reworking	2	0.4
Undetermined flakes	18	3.7
Total	490	100.0

At both sites bipolar blades and bladelets are few and do not have retouch, suggesting that their production was occasional. At Colle Rotondo blades and bladelets by direct percussion are fewer than at La Fabbrica but some were retouched, including 2 lunates and 2 truncations. The lack of dedicated bladelet cores suggests that bladelet production was just part of knapping sequences of unidirectional cores. Blades may have been produced during the initial knapping phases of unidirectional cores, as suggested by the fact that three of the five blades are cortical or partly cortical.

Dedicated bladelet cores are thus a difference between Colle Rotondo and La Fabbrica. At La Fabbrica there are two bladelet cores made by direct percussion and three made with the bipolar technique. However blades and bladelets have only some partial marginal retouch. In conclusion, at both sites blades and bladelets, whether by direct percussion or by the bipolar technique, represent only a minor component of the assemblage.

The bipolar technique appears to be more frequent at La Fabbrica (39 cores in Table 8) than at Colle Rotondo (10 cores in Table 14). However the productivity of the bipolar technique at La Fabbrica is actually rather low. Of the 39 cores in jasper only 9 produced good blanks (flakes or bladelets), the remaining cores are exhausted or failed due to bad raw material (the jasper often had fissures and fracture planes); the same is true of the 8 bipolar cores of quartz. Only 6 bipolar flakes were used as tool blanks.

**Fig 26:**
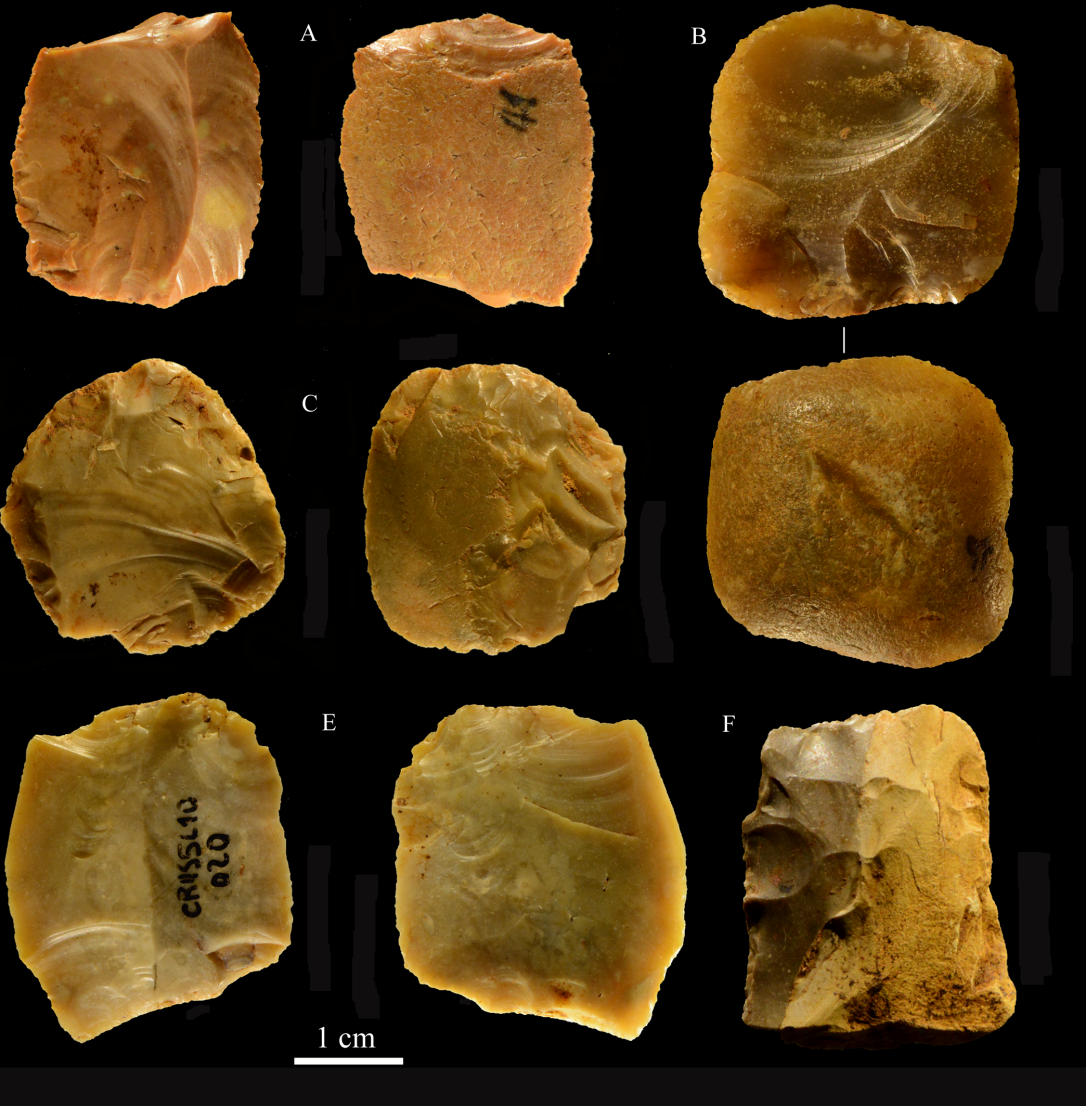
Colle Rotondo scaled pieces. Catalogue numbers: K11-2.41, K10-4.97, L11.2, L10-1.20, L11-1.44.

**Fig 27:**
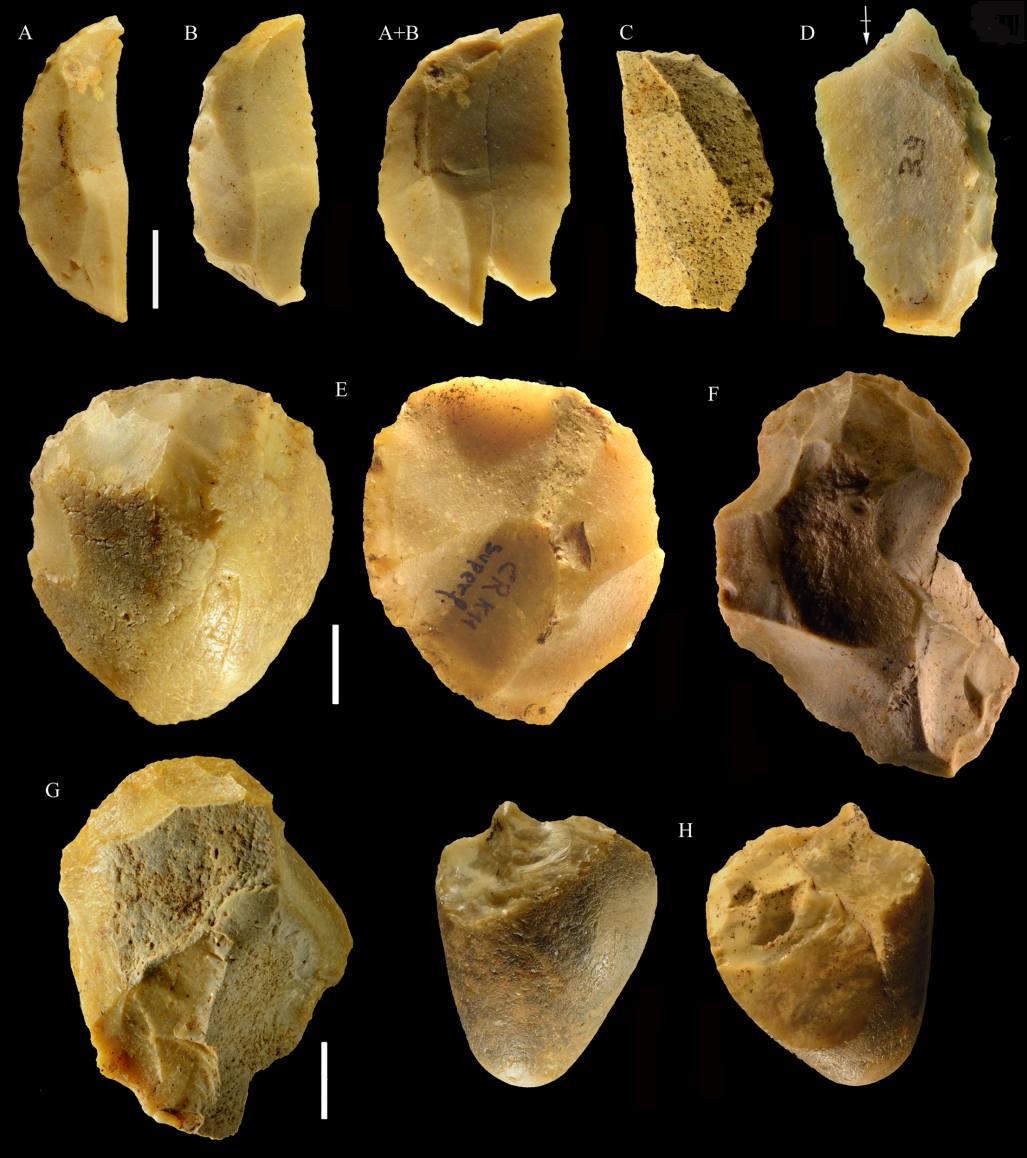
Colle Rotondo formal tools, all flint except C, F and G of chert. (A-D) Lunates, A and B are on blade; the two refit, the pieces come from the top and from the base of the layer (L 10–11 level 1 and K10 level 5). (E-G) end scrapers, E on core, F and G on flake. (H) bec on flint pebble. Scale bar = 1 cm.

**Small tools** (Tables 16 and 17, Figs 26 and 27)

**Table 16:** Colle Rotondo. Counts of small tools.

Type	N	%
Scaled pieces	36	37.9
Backed pieces (lunates)	4	4.2
End scrapers	7	7.4
Side scrapers	1	1.1
Denticulates and notches	13	13.7
Truncations	4	4.2
Bec^a^	3	3.2
Retouched and utilized blades and flakes	25	26.3
Tool fragments	2	2.1
Total	95	100.0

^a^ Bec is a commonly used French term indicating a thick awl or perforator done by alternate retouch.

**Table 17:** Colle Rotondo. Blanks of small tools.

Categories^a^	N	%
Flakes, flake fragments	64	83.1
Bipolar flakes	1	1.3
Flat flakes	1	1.3
Blades, bladelets	5	6.5
Chunks, cores	4	5.2
Pebbles	2	2.6
Total	77	100.0

^a^Undeterminate cases are excluded. As at La Fabbrica bipolar blades and bladelets were not selected as tool blanks. Bipolar flakes and flat flakes are only 1% of tool blanks.

## Discussion

Both La Fabbrica layer 2 and Colle Rotondo are flake industries with high frequencies of flakes used as tool blanks. Both assemblages are characterized by very high frequencies of scaled pieces, presence of lunates and truncations, low frequencies of bladelets and high frequencies of unprepared cores with short reduction sequences and little or no shaping of the debitage surface. These features are typical of Uluzzian assemblages as defined by Grotta del Cavallo and Castelcivita. At Castelcivita in Campania [37] the Uluzzian assemblage is also characterized by simple cores with short reduction sequences and high frequencies of scaled pieces. Scaled pieces are the dominant kind of artifact at all three sites while lunates are present in low frequencies. In all three sites blades and bladelets represent a small minority and end scrapers, made on short thick flakes, have comparable low frequencies. No Levallois cores or products are reported from the Uluzzian at Castelcivita and Grotta del Cavallo; this is also true at La Fabbrica and Colle Rotondo. There can be no doubt that La Fabbrica and Colle Rotondo assemblages are coherent and definitely Uluzzian.

Detailed comparisons of the La Fabbrica and Colle Rotondo assemblages with the Uluzzian of Castelcivita are possible only for retouched pieces (Table 18).The Castelcivita monograph is based on a predominantly typological analysis which means that specific core types, detailed classification of flakes and blades, and blanks of retouched pieces are not quantified. However the text contains useful observations and these and the numerous, clear drawings allow the assessment of some of the technological features of the assemblage indicated above. As an example, bipolar cores are not mentioned in the Castelcivita publication. However flakes described with a splintered platform (see table 15 in [37]) are clearly bipolar flakes. Very likely the counts of scaled pieces include a few bipolar cores (e.g. fig 21:8–9 in [37]). Features distinguishing scaled pieces from bipolar cores are described in the “Methods” section. The ratio of length/width of retouched and not retouched pieces in (see p. 107 in [37]) also provides data on the frequencies of unretouched and retouched blades and bladelets. This allows us to present some comparative data on debitage products from the three sites (Table 19).

**Table 18:** Retouched pieces of la Fabbrica, Colle Rotondo and Castelcivita.

Retouched pieces^a^	La Fabbrica		Colle Rotondo		Castelcivita	
	N	%	N	%	N	%
Scaled pieces	50	44.2	36	37.9	378	49.1
Backed pieces (lunates)	2	1.8	4	4.2	18	2.3
End scrapers	4	3.5	7	7.4	29	3.8
Side, transverse and convergent scrapers	10	8.8	1	1.1	121	15.7
Denticulates and notches	15	13.3	13	13.7	174	22.6
Truncations, incl. backed truncations	1	0.9	4	4.2	13	1.7
Bec	1	0.9	3	3.2	2	0.3
Retouched and utilized blades and flakes	28	24.8	25	26.3	27	3.5
Tool fragments^b^	2	1.8	2	2.1	NA	NA
Burins^c^	0	0.0	0	0.0	8	1.0
Total	113	100.0	95	100.0	770	100.0

^a^ Counts and percentages from Tables 10 and 16 and from Gambassini [37] table 4 p. 108. Comparisons of retouched pieces between La Fabbrica, Colle Rotondo with Castelcivita required some adjustment because Gambassini uses Laplace’s typology while we use a simplified version of Bordes’ typology, see S3 File for details.

^b^ There is no information on tool fragments in the Castelcivita monograph.

^c^ There are no burins at Colle Rotondo. At La Fabbrica four burins were reported in [10] but the in situ Uluzzian material contained only a possible one. However the burin facet was actually a fracture and we rejected the identification. The few burins at Castelcivita were also described as simple burins, some were said to be doubtful.

**Table 19:** Partial data on debitage at La Fabbrica, Colle Rotondo and Castelcivita.

Debitage^a^	La Fabbrica		Colle Rotondo		Castelcivita	
	N	%	N	%	N	%
Levallois cores and products	0	0	0	0	0	0
Bipolar flakes	40	9.9	22	4.5	317	13.4
Bipolar flakes used as tool blanks	6	6.7	1	1.3	15	6.8
Unretouched blades and bladelets^b^	43	10.6	19	3.9	270	11.4
Retouched blades and bladelets^b^	8	8.9	1	1.3	75	13.1

^a^Counts and percentages from Tables 9, 11, 15 and 17 and from (figs 15 and 16 in [37]).

^b^Counts of blades and bladelets at Castelcivita are based on distributions of L/W on p. 107 of [37]. They may be slight underestimates since they based on complete pieces only while counts of La Fabbrica and Colle Rotondo include broken blades and bladelets evaluated on the basis of their technical features. Bipolar blades and bladelets are excluded from these counts since we there are no observations of these items at Castelcivita.

### The problem of Dufour bladelets

Five Dufour bladelets (bladelets with marginal, semi-abrupt retouch) have been described in the Castelcivita Uluzzian. They come from near the top of the Uluzzian layer and are interpreted by [37] as precursors to the Dufour bladelets which are a typical and common artifact of the overlying Protoaurignacian. However these bladelets are inconsistent with the core types described and illustrated by [37] so they may possibly be intrusive. They are not included in the counts of retouched pieces in Table 18.

Eight Dufour bladelets in the Uluzzian of Cavallo Cave are considered by [25] as evidence of stratigraphic problems. It is interesting that Dufour bladelets are completely absent in the Uluzzian of La Fabbrica and Colle Rotondo, suggesting that the two assemblages are free of mixing. The Protoaurignacian of La Fabbrica which overlies the Uluzzian contains Dufour bladelets.

We have not done detailed comparisons with the Uluzzian layers of Grotta del Cavallo (E and D) described and abundantly illustrated by Palma di Cesnola [61–62] for two reasons: 1. Like the Castelcivita monograph the analysis of the Uluzzian layers at Cavallo is predominantly typological so only types of small tools are accurately quantified; 2. The major raw material consisted of thin slabs of siliceous limestone (“lastrine”) which were knapped by bipolar percussion producing flat blanks delimited by vertical facets. The technical constraints on the shape and type of retouch limits comparison with tools on flakes from pebbles. Scaled pieces are not included in counts of retouched pieces (following the examples of Bordes’and Laplace’s); it is true that the overlap between scaled pieces, bipolar cores and bipolar products at the time precluded a clear distinction. Thus the frequency of scaled pieces may be as high as 70% of all small tools in the lowest level (E III). The lack of detailed information on retouched blades and flakes with partial or marginal retouch is an additional problem for calculating percentages.

The ongoing controversy about the stratigraphic position of two modern human teeth attributed to the Uluzzian layers [3, 25, 63, 64] concerns the question of whether the Cavallo artifact assemblages contains some lithic material derived from disturbed areas of the deposit. However, this does not prevent us from assessing the general features of the Cavallo assemblage which correspond with what we know from the other three sites: low proportions of end scrapers and backed pieces, very high numbers of scaled pieces, a good presence of scrapers and denticulates and very low numbers of bladelets.

This is clear evidence that the Uluzzian is a coherent and valid culture-stratigraphic unit with an approximate duration of 5 millennia. We find difficult to fit in this picture the Uluzzian assemblages from Fumane Cave of layers A4 and A3 [7]. The Levallois technology is present in both layers, especially in A4. Counts of Table 1 in [7] show that in A4 Levallois cores are 44% of all flake cores (8 of 18, excluding blade and bladelet cores) and Levallois flakes are 34% of all flakes (152 of 452, excluding undetermined flake and flake fragments). In A3 there are no Levallois cores but Levallois flakes are 11% of all flakes (51 of 446). Scaled pieces are present in very low quantities (3.8 and 7.4% in A4 and A3 respectively) while scrapers, such as side scrapers, convergent and transverse (see Table 2 in [7]) are the most common type of tool in both layers. Specifically the presence of Levallois cores and products, which are completely absent in sites from southern and central Italy (La Fabbrica, Colle Rotondo, Castelcivita, Cavallo) implies that the Uluzzian of Fumane developed with a different trajectory compared to those sites, as also suggested by the very low frequencies of scaled pieces. Surprisingly the assemblage from Riparo del Broion, 60 km east of Fumane, appears to have all the typical features of the Uluzzian [8].

### The origins of the Uluzzian

Three alternative hypotheses have been proposed for the authorship of the Uluzzian: that it is the product of modern humans dispersing into Eurasia from the African continent [3–4], that Neandertals were the makers of the Uluzzian [36] or that it may have been made by social mixed groups, given the genetic evidence for interbreeding between Neandertals and anatomically modern humans [7, 65]. The absence of fossil human remains in Uluzzian layers at Castelcivita, La Fabbrica, Colle Rotondo and Fumane (where a fragment of a permanent molar is said to be undiagnostic [66] and the controversy on the stratigraphic position of human teeth at Grotta del Cavallo, identified as modern humans by [3] implies that, pending the discovery of DNA or further human remains, these hypotheses must be evaluated by archaeological arguments.

The late age of the Klissoura assemblage would seem to exclude a migration of populations from Greece into Italy [14]. We agree with Douka et al. [5] that the origins of the Uluzzian cannot be sought in industries of Howiesons Poort affinities from South Africa or East Africa because there is no evidence of Uluzzian assemblages in the intermediate regions. We can also note that the classic Howiesons Poort industry, which is rich in backed pieces and dated between c. 65 to 59 ka [67–69] is defined by a debitage almost exclusively oriented to the production of blades, hardly comparable to the Uluzzian. The post-Howiesons Poort industries are characterized by a significant production of flakes as tool blanks, however blades are still largely predominant at Border Cave (layer 2WA dated to about 60 ka) [35] at Sibudu (layer RSP, dated to about 53 ka) [70] and in the MSA3 of Klasies (dated between 60 and 50 ka) [69]. In contrast flake manufacture is predominant in the post-HP at Rose Cottage (between 50 to 47 ka) [67]. However it is hardly possible to argue for a migration wave of modern humans from Southern or East Africa after 60 ka. In the Levant modern humans are documented at 55 ka at Manot Cave [71]. Because the Manot cranium is unequivocally modern yet different from most other early anatomically modern humans in the Levant, it was suggested that the Manot people might be closely related to the first modern humans who later successfully colonized Europe.

We can look at evidence of continuities or discontinuities between the Uluzzian and the preceding and succeeding culture units in Italy. We are aware of the fact that techno-typology or stratigraphic continuity/discontinuity are not necessarily a predictor of hominin taxonomy. But most work in science is purely observational and we expect that the implications of the patterns we describe will require some time to fall into place and may provide some insights into a theory of cultural transmission and relatedness which in prehistory is at the elementary level.

### The Uluzzian and the Mousterian

The Uluzzian occupation is sometime, but not always, separated from the Mousterian by a hiatus in the stratigraphic sequence. At Castelcivita the stratigraphic contact between the Mousterian and the Uluzzian is marked by a clear sedimentological difference indicating a hiatus (of unknown length) [37]. At Grotta del Cavallo the Uluzzian is separated from the underlying Mousterian by a thin level of green volcanic sand, the Green Tuff of Pantelleria dated to 45.5 ± 1.0 ka [18]. At La Fabbrica an erosional surface separates the thin Uluzzian layer (layer 2) from the underlying Mousterian (layer 1a). The OSL dates also suggest a temporal interval, since layer 1a is dated to 44±2.1 and layer 2 is dated to 40 ± 1.6 ka (S2 File).

At Fumane on the contrary there is no separation between the last Mousterian layer (A5) and the first Uluzzian layer (A4). The ^14^C dates partly overlap [72] and in some area there was mixing of materials [7]. At Grotta Bernardini which is near Grotta del Cavallo (Fig 1) there is no stratigraphic discontinuity between the Uluzzian and the final Mousterian [73].

According to several authors [11, 15, 37] there is no evidence of cultural continuity between the final Mousterian of Italy, often characterized by a Levallois debitage, and the Uluzzian. Clearly Fumane is an exception.

High frequencies of side scrapers and denticulates in the Uluzzian of Cavallo and Castelcivita (Table 19) have been cited as persistence of Mousterian elements [15] but denticulates, notches and side scrapers on flake are also present in non- insignificant quantities in the Protoaurignacian [1, 74]. Excluding notches and denticulates which may be considered as indispensable tools, the proportions of Middle Paleolithic types (side scrapers, end scrapers on flake, becs, even choppers or Mousterian points) in Aurignacian, Gravettian, Solutrean and Magdalenian assemblages from sites such as Le Flageolet I and II, Laugerie Haute Est and Ouest, La Ferrassie, La Madeleine and Corbiac can be as high as 8 to 15% in Aurignacian and Gravettian assemblages, 17% in Solutrean assemblages and only less than 5% in Magdalenian assemblages (see SI Appendix p. 24 in [35]). Clearly typochronology has predictive limits in the Paleolithic if only simple flake tools are considered.

A debitage oriented to the production of flakes is certainly not a characteristics of the Early Upper Paleolithic. To find a Western European industry with an abundance of flakes used as tools blanks we have to wait about 20,000 years for the Badegoulian which is dated between 22 and 20 ka calibrated BP [75]. However the assemblage composition, reduction sequences and tool types of the Badegoulian are completely different from the Uluzzian [76].

Unprepared cores with short reduction sequences are a characteristic feature of the Uluzzian. This kind of cores occurs throughout the Paleolithic but they are a common component of the Middle Paleolithic and are found also in the Châtelperronian [77]. This kind of cores cannot be attributed to a bad raw material. At La Fabbrica Mousterian craftsmen were making Levallois cores on the same jasper used by the Uluzzians. At Cavallo the Mousterians were making Levallois cores and flakes and even blades with on the same siliceous slabs of limestone on which Uluzzian were making scaled pieces, denticulates and bladelets. Thus the Uluzzian debitage was by choice, not by constraints. On balance, however, the evidence in favor of continuity between the Mousterian and the Uluzzian is limited.

The general view seems that elements such as very high frequencies of scaled pieces, bone tools, bladelets, lunates, use of ochre and a few marine shell ornaments (found at Cavallo but not at Castelcivita, La Fabbrica, Colle Rotondo and Fumane) are seen as innovations that distinguish and separate the Uluzzian from the Mousterian. The significance of these components is discussed below.

### Are the Uluzzian “innovations” unequivocal indicators of a modern human culture?

#### Scaled pieces

Scaled pieces (the French term is pièces esquillées) are not indicators of specific industries. As is often the case with stone artifacts their significance depends on frequencies. Scaled pieces occur in the Developed Oldowan of Middle Bed II at Olduvai, in the Acheulian site of TK in Upper Bed II and in other Olduvai localities up to the Masek Beds [78] but hey always represent less than 5% of the tool component. Scaled pieces occur in low frequencies in the MSA industries of South Africa. Bipolar percussion and scaled pieces show a significant increase after 49–45 ka cal BP and especially at 44–42 ka cal BP at Border Cave [35].

Compared to the Mousterian we do observe a strong increase in the number of scaled pieces in the Uluzzian of Castelcivita, Cavallo, La Fabbrica and Colle Rotondo (Table 18), in the Châtelperronian of Grotte du Renne (10.8% in layer Xc) [79] in the Protoaurignacian with Dufour bladelets of Castelcivita (12%) [37] and the Protoaurignacian of Grotte du Renne (21.3%) [74] as well as in the Aurignacian of Brassempouy (44% in layer 2F) [80] and of Flageolet I (22% in layer XI) [81] in southern France.

In general, Italian Mousterian assemblages contain scaled pieces in low frequencies. In the Mousterian of Castelcivita scaled pieces are 2.2% (10 of 444) (see p. 101 in [37]). There are no scaled pieces in the Middle Paleolithic assemblages of Latium (so-called Pontinian) dated to MIS 3 (Guattari, Sant’Agostino) ([82] and ongoing research by Sylvain Soriano and PV) However, higher frequencies are sometime documented in late Mousterian layers. This is the case of Fossellone level 23 alpha which is the last Mousterian separated from the Aurignacian above by a sterile layer. Scaled pieces there are 27.3% (62 of 227; ongoing research by PV and Sylvain Soriano). At Grotta del Cavallo the final Mousterian layers FIIIa-FI have 37% of scaled pieces (59/159) [73]. However the Cavallo scaled pieces may include bipolar cores which were not kept separate.

#### Personal ornaments and use of ochre

Archaeological evidence shows that personal ornaments such as perforated and ochred marine shells are documented in the Mousterian at Cueva de los Aviones and Cueva Anton in Spain [83–84] and at Fumane [85]. The use of eagle claws as pendants is documented by cutmarks on terminal phalanges of eagles at eight Mousterian sites in France, Italy and Croatia (Pech de l’Azé I, Combe Grenal, les Fieux, Pech de l’Azé IV, Grotte Mandrin, Rio Secco, Fumane, Krapina) indicating the removal of the claw [86–91].

Fragments of non-local hematite have been found at Maastricht-Belvédère Site C, dated to minimally MIS 7 (250–200 ka). Lumps of ochre and ochre staining on stalagmite containers have been found also at other sites such as Amsterade-Allée (Netherlands) with an estimated date 75,000 BP, Les Bossats (France) dated to 47,000 y ago and Ciaorei Cave in Romania, proving that the use of red ochre was a component of Neandertal cultural behavior [92–96]. Seven Châtelperronian sites have yielded reliable evidence of the use of red pigment; lumps of pigment from three of these sites have been verified by elemental and mineralogical analysis [97].

#### Bladelets

The idea that backed bladelets provided modern humans with projectile weaponry that gave them a huge advantage on Neandertals equipped only with hand-cast spears, once modern humans moved out of Africa, was suggested a few years ago by Brown et al [98]. Supporting evidence was provided by (a) occurrences of backed bladelets in South Africa at the Pinnacle Point Site 5–6 in layers dated between 71 and 60 ka, and (b) the idea that the success of bladelet production in Eurasia began with the Ahmarian in the Levant and in the Proto-Aurignacian in Western Europe. In fact, the evidence of bladelet production in layers 29–30 at Combe Grenal (SW France) dated to MIS 4, about 70–60 ka [99], of bladelets and microlithic points at Grotte Mandrin (Rhone Valley, France) dated to about 50 ka [100] suggest that bladelet manufacture and projectile technology were already available to Neandertals before the arrival of modern humans. A recent study of the late Mousterian industry from Grotta del Cavallo layer FIIIe also shows the autonomous development of a bladelet and blade reduction sequence by direct percussion and using the same thin slabs of siliceous limestone that were knapped by Uluzzians in layers EIII to D [101].

#### Bone tools

Formal bone tools made with techniques specifically conceived for bone materials such as splitting and wedging, scraping and polishing are common in Upper Paleolithic sites but some also occur in the Mousterian. Four lissoirs (a French term meaning “smoothers”) described by [102] come from two Mousterian of Acheulian Tradition sites, Pech de l’Azé 1 and Abri Peyrony in southwest France and dated 51.4±2.0 ka and between 47 and 41ka cal BP respectively. These are tools made on ungulate ribs and shaped by grinding and scraping.

In the Châtelperronian of Grotte du Renne 48 bone awls made by scraping and some marked with notches have been studied by [40]. Almost all come from the lowest Châtelperronian level X.

### Concluding remarks on the Uluzzian

It could be argued that many of these apparent novelties were just episodically present in Neandertal assemblages and only later became a stable component of cultural behavior and that it is in the Uluzzian that these novelties appear to be stable elements. However this review makes it clear that the question of hominin authorship cannot be answered by an archaeological approach to patterns of continuity or discontinuity because in the temporal context of “transitional industries” technology and typology are in fact controlled by too many variables. Innovations and adoptions of new technologies are not the exclusive behavior of one hominin species, as exemplified by the Châtelperronian, defined as a fully Upper Paleolithic industry with blade and bladelet production and bone-tool production yet made by Neandertals [79, 103–110]. This is now confirmed by molecular evidence of small bone fragments from the Châtelperronian layers at Grotte du Renne [111].

The replacement of the Mousterian by the Uluzzian with all its defining elements and the replacement of the Uluzzian by the Protoaurignacian appear rapid with no evidence of a gradual change. A similar abrupt replacement of the last Mousterian level by the Protoaurignacian has been observed at Grotte Mandrin (Rhone Valley, France) where a very short time, in the order of a few seasons or a few years, separates the two occupations. This is documented by a precise occupation sequence established by using sooted concretions and clasts in stratigraphic order [112]. This supports the hypothesis proposed by several authors that at the end of the Middle Paleolithic both Neandertals and modern humans were contemporaneous and living in the same region.

If we consider that the Protoaurignacian arrived in southwest Europe, including Italy, between 42,000–41,000 [9, 113, 114] this suggests that, prior to that time,only Neandertals existed in Italy and the Uluzzian and the Châtelperronian, both documented well before 42 ka, in fact between 45 and 40 ka [18,109] are industries not affected by the spread of modern humans except at the end of their temporal span.

An alternative hypothesis is that the “innovative” elements such as blades, bladelets, bone tools and ornaments would indicate modern culture influence through stimulus diffusion (i.e. acculturation) [110] since the date of the Aurignacian has been pushed back to 43.5 in central Europe [115]. However recent work has shown that the dates of 45–40 ka suggested for the appearance of modern humans in Europe (at Kent’s Cavern and Cavallo) are questionable and the date of 43.5 ka for the first appearance of the Aurignacian in Central Europe is unsupported (21). Furthermore we have shown that these innovative elements also occur in older Mousterian assemblages. The rapid replacement of a technology or cultural unit by another cannot be taken as an indication of migrating moderns taking over a foreign territory since the same pattern of rapid discontinuity and replacements has been observed within the Mousterian sequence between the Quina Mousterian and the Neronian (the latter is a Middle Paleolithic industry in the Rhone valley, with bladelets and microlithic points, dated to about 50 ka cal BP [100]. We take this as a further indication that at for this time period the typo-technology as an indicator of hominin authorship has limited prediction value.

## Conclusions

This research has allowed us: (a) to present detailed technological and typological analyses of the assemblages from Grotta La Fabbrica and from the new site of Colle Rotondo with particular attention to each site age, chronostratigraphic context and taphonomy (b) to show that the general technological and typological features of the assemblages of Castelcivita, Grotta del Cavallo, La Fabbrica and Colle Rotondo define the Uluzzian as a valid cultural unit with an approximate duration of five millennia. The Uluzzian was replaced by the Protoaurignacian about one or short of two millennia before the eruption of the Campanian Ignimbrite; (c) to show that the question of who were the makers of the Uluzzian cannot be answered by an archaeological approach to patterns of stratigraphic and techno-typological continuity or discontinuity. In the specific context of transitional industries, with disputed dates about the arrival of modern humans in Western Europe and assemblages from central and southeastern Europe only partly known, technology and typology cannot provide conclusive evidence of hominin taxonomy and there is ongoing controversy about the stratigraphic position of two modern human teeth attributed to the Uluzzian layers of Grotta del Cavallo. The answer will have to come from DNA evidence and/or more human remains. DNA analysis of La Fabbrica sediment is being planned. (d) Our work confirms previous suggestions [113] that the Middle-to-Upper Paleolithic transition was not one of gradual transformation but that it occurred as steps of rapid changes with some site abandonment and geographically uneven rates of spread, as documented by the 3,000 year persistence of Neandertals in Iberia south of the Ebro frontier, the abandonment of the Cavallo site, the rapid replacement of the Uluzzian by the Protoaurignacian and the short duration of the Protoaurignacian occupation at sites in southern Italy impacted by the Campanian Ignimbrite eruption.

## Supporting information

S1 File Figures. (PDF)

S2 File OSL dating of Grotta La Fabbrica and Colle Rotondo. (DOCX)

S3 File Lithic analysis. (PDF)

S4 File X-ray diffraction and chemical analysis. (DOCX)

S5 File Permissions from copyright holders. (PDF)
