# Supplementary material for: From Neandertals to modern humans: New data on the Uluzzian
Source: PLoS One. 2018 May 9;13(5):e0196786. doi: 10.1371/journal.pone.0196786 (PMC5942857; doi:10.1371/journal.pone.0196786)
Supplement: S1 File — (PDF) [file pone.0196786.s001.pdf]

Supporting Information

From Neandertals to modern humans: New data on the Uluzzian

Paola Villa*, Luca Pollarolo, Jacopo Conforti, Fabrizio Marra, Cristian Biagioni, Ilaria Degano, Jeannette J. Lucejko, Carlo Tozzi, Massimo Pennacchioni, Giovanni Zanchetta, Cristiano Nicosia, Marco Martini, Emanuela Sibilia, Laura Panzeri.

*villap@colorado.edu

S1 File. Figures

This PDF file includes:
S1 File, Figures A-H

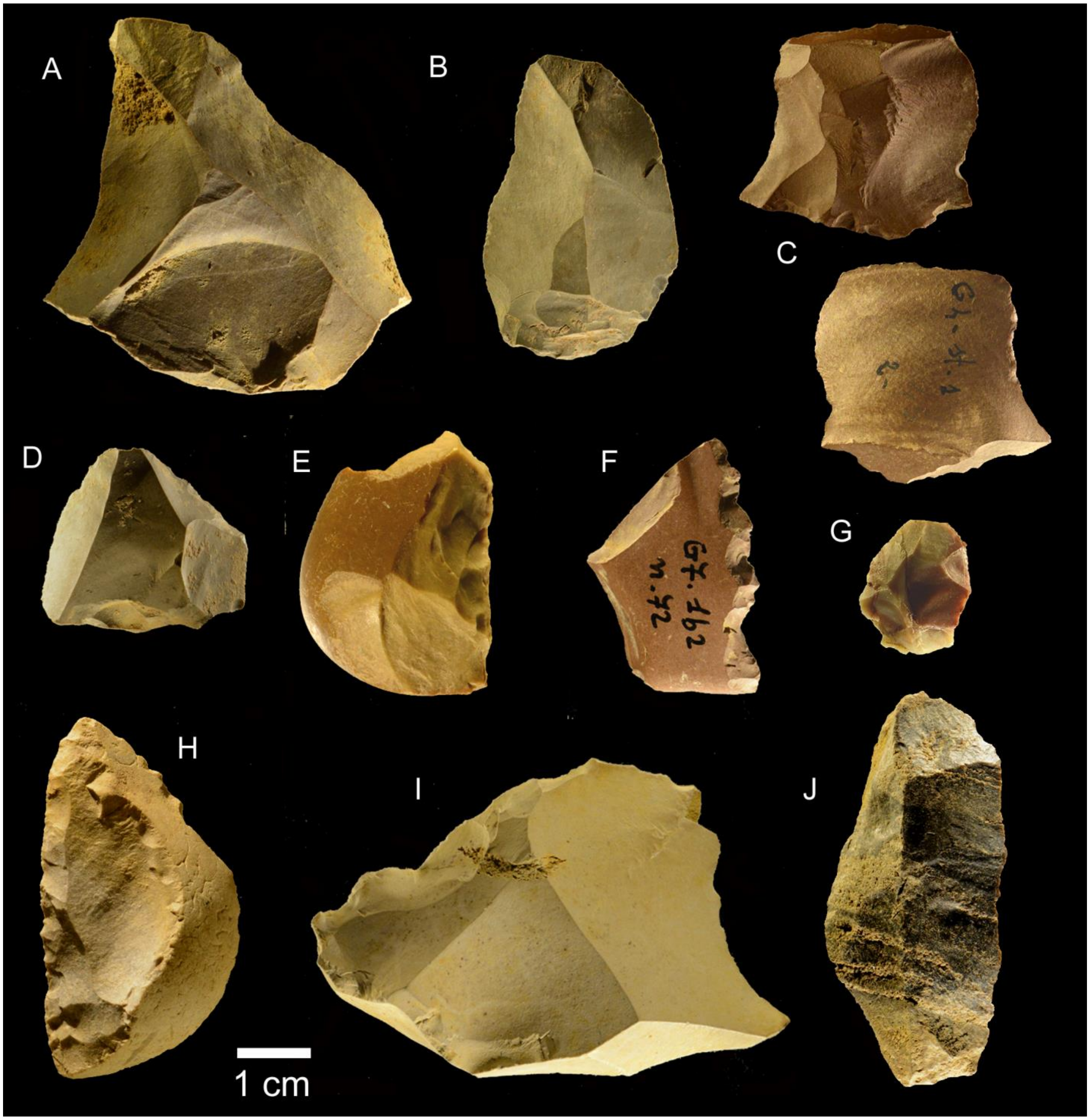

Figure A. Grotta La Fabbrica, Mousterian. (A, B, D) Unretouched Levallois flakes (A, B chert, D flint). (C). Flake with unidirectional scars and distal cortex, with marginal retouch, jasper. (E) Side scraper on a flint flake with unidirectional scars and lateral cortex. (F) Denticulate on a jasper flake fragment. (G) Levallois flake with partial retouch, flint (H) Side scraper on a chert flake with bidirectional scars and lateral cortex. (I) Denticulate on a Levallois flake of chert. (J) Unretouched blade of chert with lateral cortical edge.

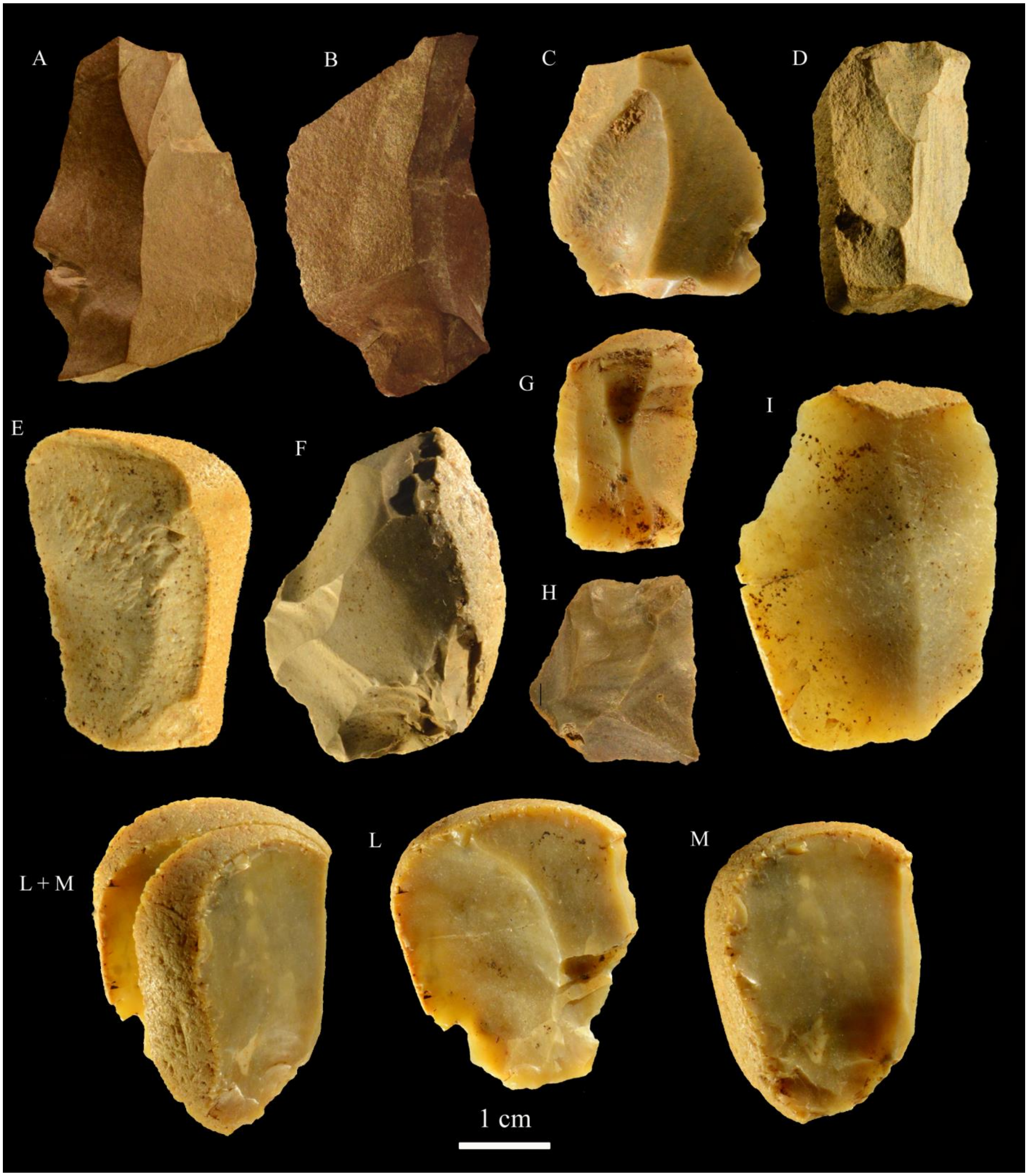

Figure B. Uluzzian. Flakes produced from non-Levallois cores with series of unidirectional, bidirectional or multidirectional removals without any preparation of the debitage surface or very limited shaping of the cores. They may be without cortex (A, C, H) or have a cortical abrupt back or end (D - G, I, L, M) or an oblique cortical facet on the dorsal face (B). (A,B, D, H) from La Fabbrica, (C, E-G, I-M) from Colle Rotondo. (L+M) is a refit of two flakes from Colle Rotondo, square L10-11 level 1 and K 10 level 3.

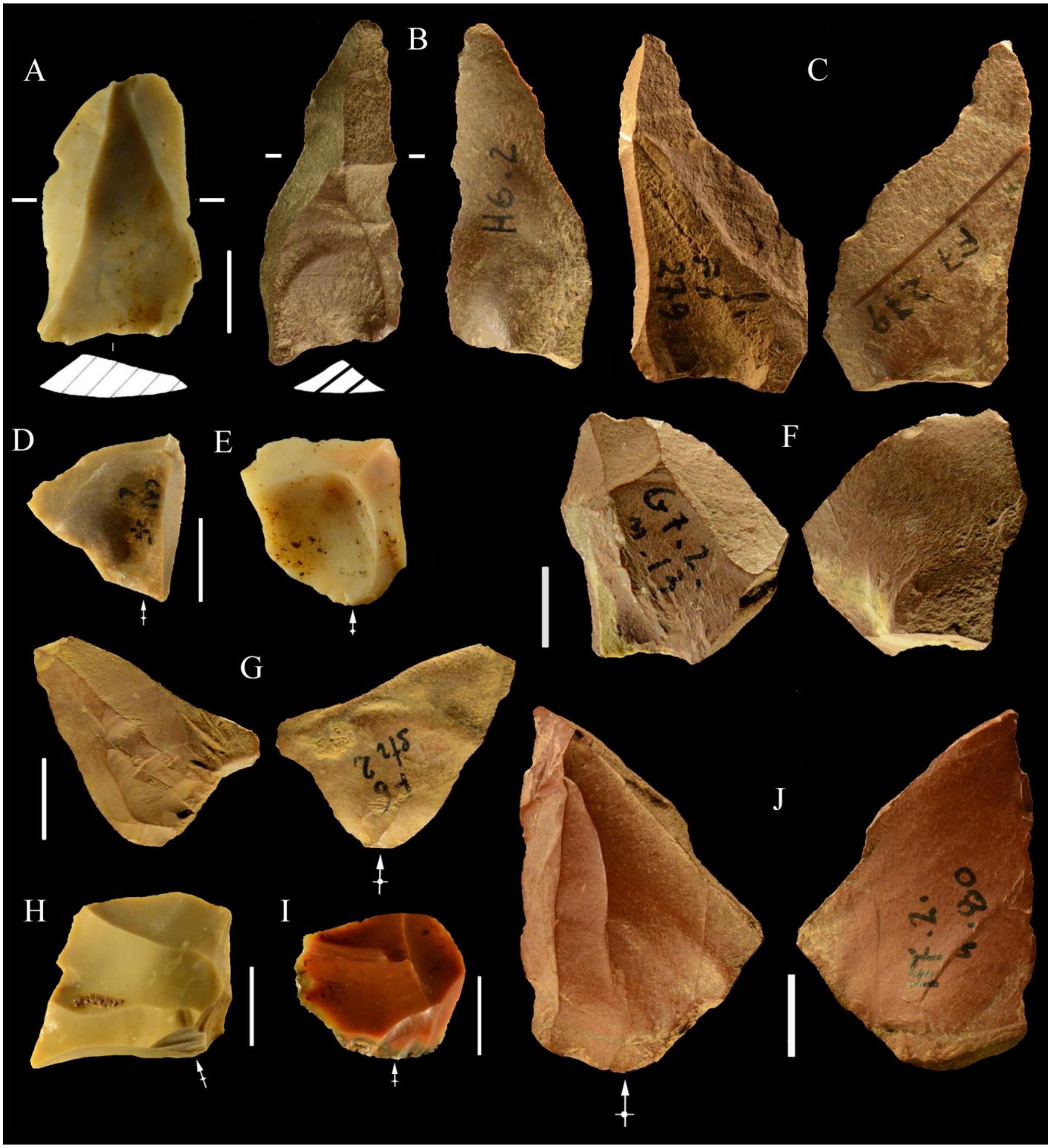

Figure C. Uluzzian. (A-C) are similar to Levallois flakes and might be mistakenly classified as Levallois. Note however that flake scars on the dorsal face to the left of each flake is very oblique (i.e. secant, see transverse section of A) instead of parallel to the debitage surface of the other scars, as expected if these were Levallois flakes. In all three cases the platform is thin and plain. In other words these flakes have been produced by non-Levallois cores like those illustrated in Figs. 11 and 20. (D, E, F, G, J) pseudo-Levallois points. (H) is a débordant flake with unidirectional scars of laminar flakes, orthogonal to the debitage axis. (A, D, E, H, I) from Colle Rotondo; (F, G, J) from La Fabbrica. (J) has partial retouch at the tip. (I) is a centripetal, non-Levallois flake. Scale bar = 1 cm.

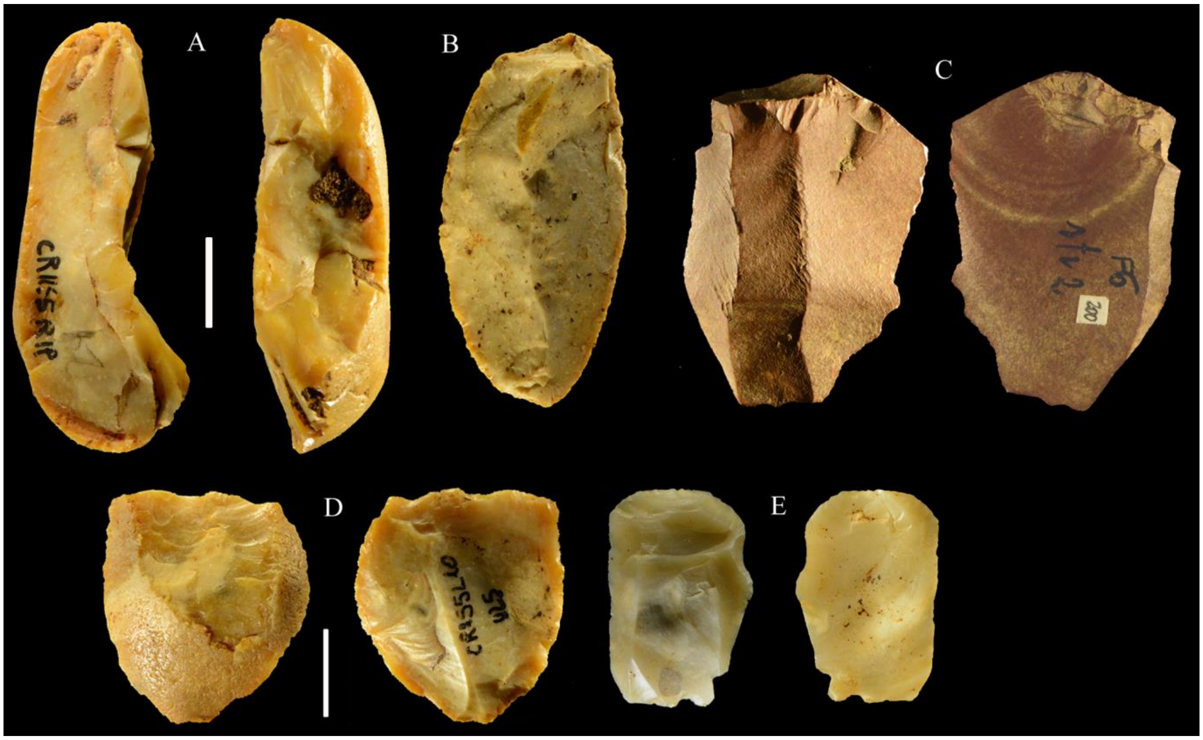

Figure D. Uluzzian. (A) Bipolar core. (B) Bipolar laminar flake. (C) Bipolar flake. (D-E) flakes from scaled pieces. (A, B, D, E) from Colle Rotondo, (C) from the Uluzzian of La Fabbrica. Scale bar = 1 cm.

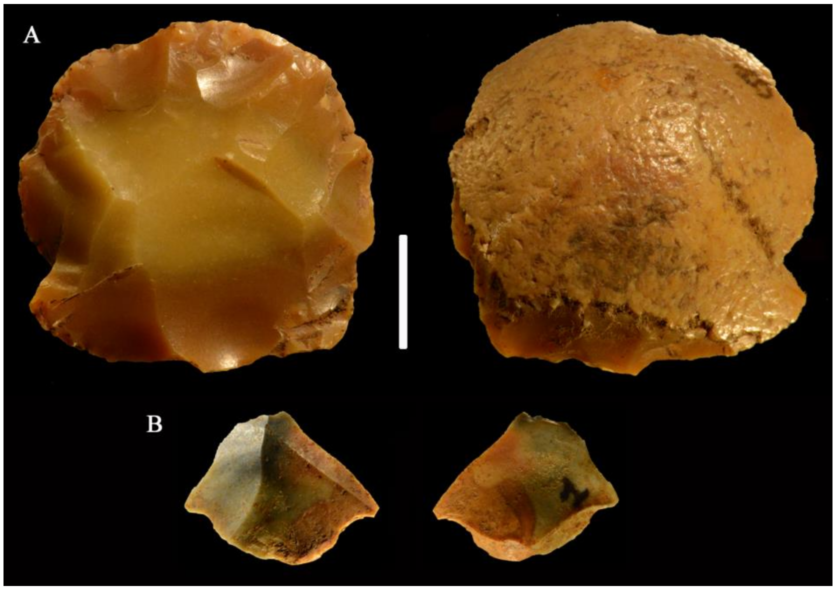

Figure E. Colle Rotondo. Levallois core with preferential flake scar. The Levallois flake has a facetted platform, it does not refit on the core. Note the fluviatile polish and microfractures on edges. These two pieces are redeposited and are not part of the Uluzzian assemblage (see Colle Rotondo "Taphonomy" in main text). Scale bar = 1 cm.

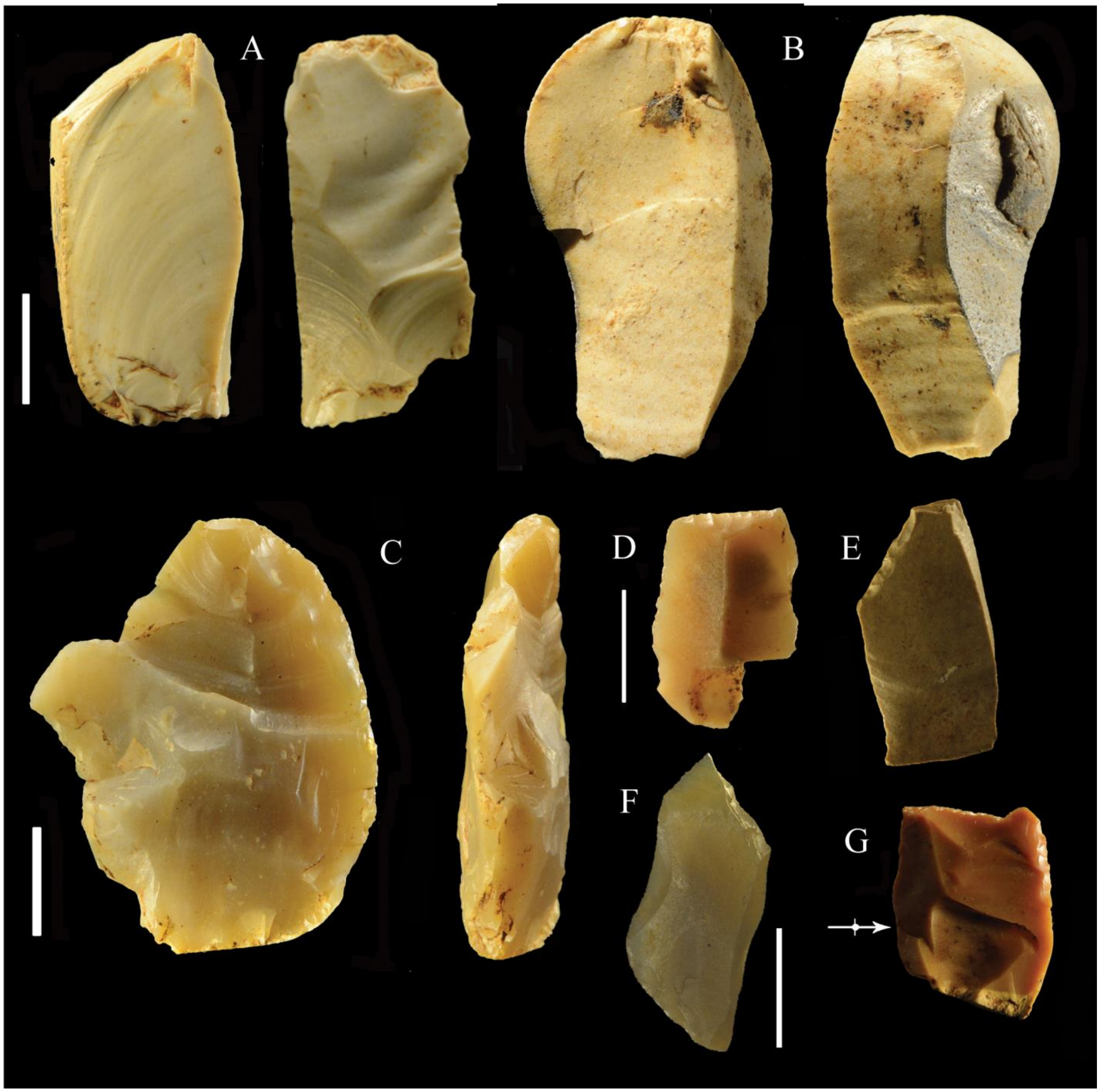

Figure F. Colle Rotondo. (A-C) bipolar cores. (D-G) truncations. (D) distal truncation on blade; (E) oblique truncation on a flake with a Siret break; (F) oblique truncation on a bladelet; (G) on a bipolar flake, the abrupt retouch is lateral. Scale bars = 1 cm.

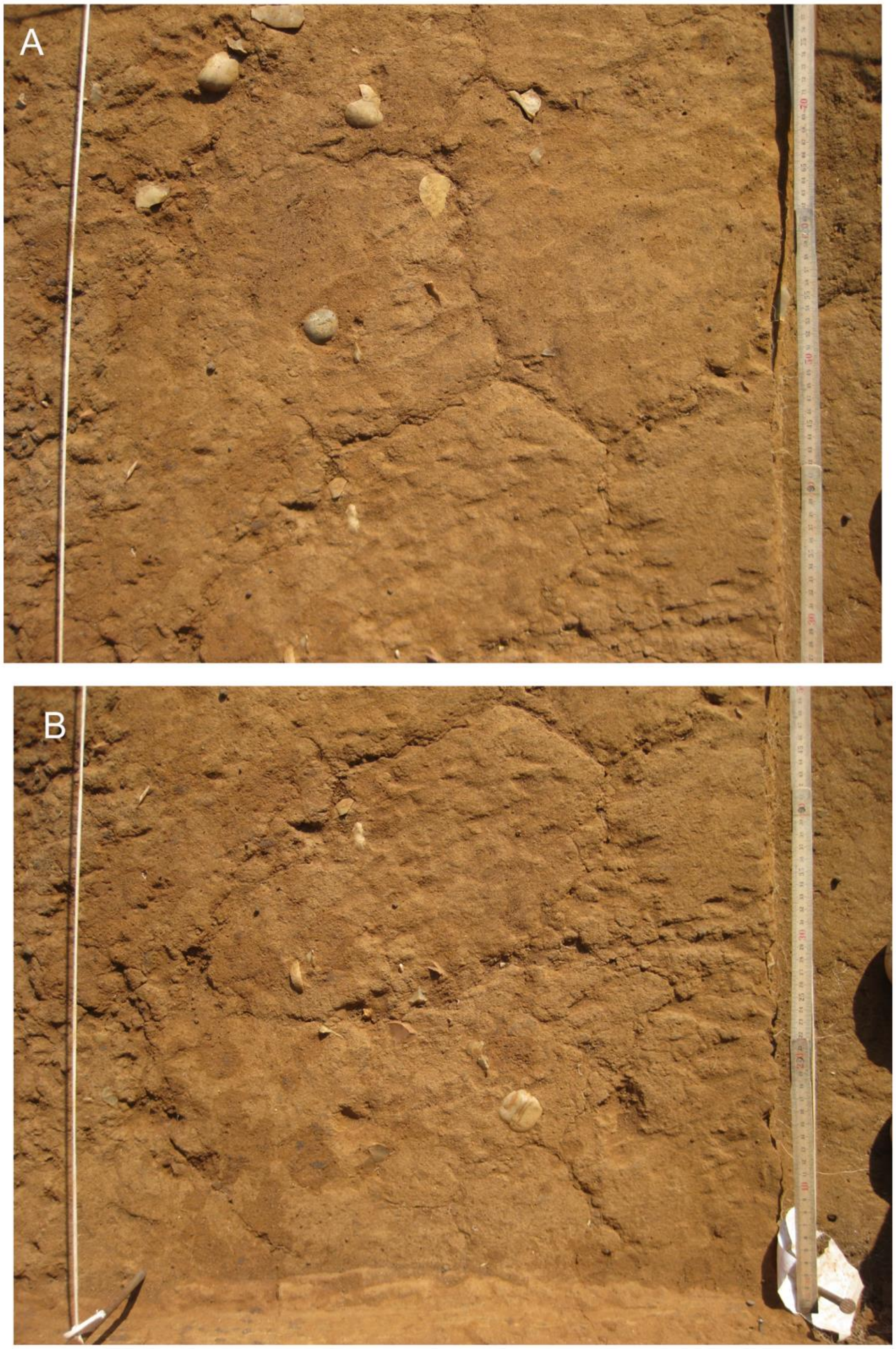

Figure G. Colle Rotondo. View of localized small channeling and run-off in square K10 (A and B overlap).

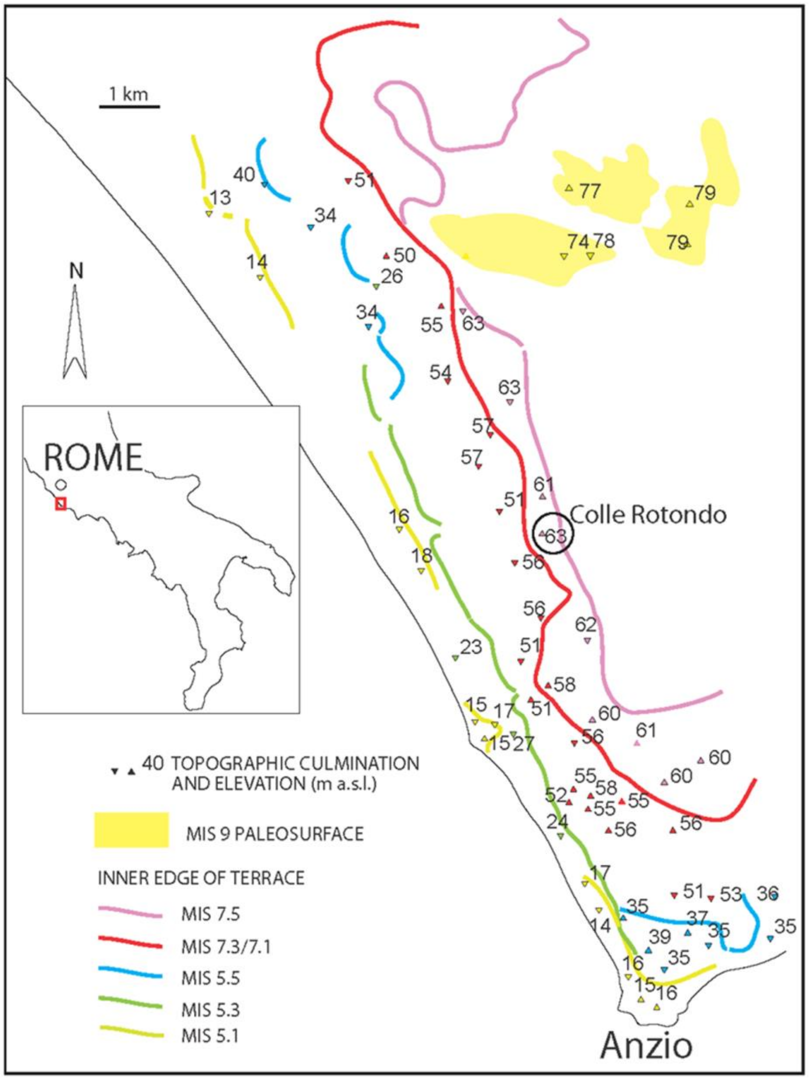

Figure H. Coastal terraces in southern Latium.
