# Supplementary material for: From Neandertals to modern humans: New data on the Uluzzian
Source: PLoS One. 2018 May 9;13(5):e0196786. doi: 10.1371/journal.pone.0196786 (PMC5942857; doi:10.1371/journal.pone.0196786)
Supplement: S2 File — (DOCX) [file pone.0196786.s002.docx]

**Supporting Information**

**From Neandertals to modern humans:**

**New data on the Uluzzian**

**Paola Villa*, Luca Pollarolo, Jacopo Conforti, Fabrizio Marra, Cristian Biagioni, Ilaria Degano, Jeannette J. Lucejko, Carlo Tozzi, Massimo Pennacchioni, Giovanni Zanchetta, Cristiano Nicosia*,* Marco Martini, Emanuela Sibilia,Laura Panzeri.**

*[villap@colorado.edu](mailto:villap@colorado.edu)

**S2 File.** OSL Dating of Grotta La Fabbrica and Colle Rotondo

This file includes: OSL Analytical procedures and results

Figures A-B

Table A

References

**Analytical procedures and results**

OSL analyses were carried out at the Dipartimento di Scienza dei Materiali, Università di Milano-Bicocca, Italy. Samples were prepared under controlled red light conditions following the conventional procedure [1-3]. They were wet sieved to extract the 180-250 μm sand fraction that was chemically treated to eliminate organic matter (H_2_O_2_ 36vol for 4 hours) and carbonates (HCl 37% for 4 hours). The separation of quartz was obtained by gravimetric separation using sodium politungstate (Na_6_[H_2_W_12_O_40_]·xH_2_O) followed by hydrofluoric acid treatment (40%; 40 minutes) to remove feldspars and the outer α-irradiated external skin. A further HCl treatment (15%, 15 minutes) was done. Quartz grains were fixed on stainless steel discs (10mm diameter) with silicon oil.

The measurements were performed following the Single-Aliquot Regeneration (SAR) dating protocol [4] using an automated luminescence system (Risø TL/OSLDA-20) equipped with a ^90^Sr/^90^Y beta source delivering 0.12 Gy/s (± 3%) to the sample position. Quartz OSL was stimulated by an array of blue LEDs *(*470 ± 30 nm) with a constant stimulation power of 54 mW/cm^2^ and OSL shine-down curves were detected using a photon counting technique with an EMI 9635QB photomultiplier tube coupled to a 7.5 mm Hoya U-340 filter. The samples were checked for the absence of feldspar contamination using IR stimulation (830 ± 10 nm; constant stimulation power of 360 mW/cm^2^) on irradiated samples.

To calculate the annual dose, Th and U concentrations of the sediments were measured with total alpha counting using ZnS scintillator discs [5]. ^40^K content was deduced from the total concentration of K, obtained by flame photometry. Attenuation of the beta dose was taken into account [6]. The cosmic ray contribution to the final dose rate was based on [7]. The concentrations of the principal radionuclides, the assumed average water content and the corresponding annual dose rates are shown in Table A.

OSL measurements (100 s) were made at 125°C; the preheat value was experimentally derived from the results of a dose recovery pre-heat plateau test (260°C for samples from Grotta La Fabbrica and 240°C for sample from Colle Rotondo). A cut-heat of 200°C was applied to each aliquot before the test dose measurement. To evaluate the Equivalent Dose (*D*_e_), the initial part of the OSL decay curve was used, specifically the first 1.2 s. The background was assumed as the mean signal of the last 8 s of stimulation. Example of OSL SAR growth curves are reported in Figure A. The thermal transfer was evaluated by calculating the recuperation point, which never exceeded 5% of the natural emission [8]. All aliquots had acceptable recycling values, i.e. within the range of 0.90 and 1.10 [9-10].

The OSL age of quartz samples is limited by the saturation of the natural signal for the high dose values (Wintle and Murray, 2006). If the D_e_ of a sample is lower than 85% of the saturation point (2D_0_), the OSL age corresponds to a reliable indication of the sedimentary event, otherwise it corresponds to a *minimum age*. For the samples from Colle Rotondo, a relevant percentage of the total aliquots analyzed for each samples was beyond the saturation point (see table A) and they were not included in the D_e_ evaluation.

In Figure B the equivalent dose (D_e_) distributions are reported. The dating results are summarized in Table A.

S2 File, Figure A. Example of OSL SAR growth curve obtained on the analyzed samples.

S2 File, Figure B. Example of the Equivalent Dose distribution obtained for the samples.

Table A. Concentrations of the principal radionuclides, average water content, dose rates, equivalent dose and ages obtained with OSL.

| **Location** | **Sample** | **% of aliquots beyond the D_e_ saturation** | **ppm U**  **( ± 5%)** | **ppm Th**  **(± 5%)** | **ppm ^40^K**  **(± 3%)** | **% H2O**  **(± 5 %)** | **Dose rate**  **(mGy/a)** | **D_e_**  **(Gy)** | **Ages**  **(years)** |
| --- | --- | --- | --- | --- | --- | --- | --- | --- | --- |
| **Grotta La Fabbrica** | **GeGLF1** | 0 | 0.95 | 2.84 | 0.75 | 10 | 0.98 ± 0.03 | 43.1 ± 1.6 | 44,000 ± 2,100 |
|  | **GeGLF2** | 0 | 0.91 | 2.74 | 0.90 | 10 | 1.07 ± 0.03 | 42.8 ± 1.3 | 40,000 ± 1,600 |
| **Colle Rotondo** | **CR1** | 31 | 5.47 | 17.27 | 2.99 | 22 | 4.01 ± 0.12 | 58.7 ± 3.4 | 14,640 ± 960 |
|  | **CR2** | 47 | 5.08 | 16.06 | 2.25 | 22 | 3.41 ± 0.10 | 54.1 ± 3.4 | 15,870 ± 1,100 |
|  | **CR3** | 62 | 4.20 | 13.26 | 3.82 | 22 | 4.06 ± 0.12 | 73.8 ± 3.2 | 18,180 ± 950 |

**References**

1. Stokes, S. (1992). Optical dating of young (modern) sediments using quartz: results from a selection of 26 depositional environments. *Quaternary Sci. Rev., 11, 153-159.*

2. Mejdahl, V. and Christiansen, H.H. (1994). Procedures used for luminescence dating of sediments. *Quaternary Sci. Rev., 13, 403-406.*

3. Lang, A., Lindauer, S., Kuhn, R. and Wagner, G.A. (1996). Procedures used for Optically and Infrared Stimulate Luminescence Dating of Sediments in Heidelberg. *Ancient TL, 14, 7-11*.

4. Murray, A.S. and Wintle, A.G. 2000. [Luminescence dating of quartz using an improved single-aliquot regenerative-dose protocol](http://www.sciencedirect.com/science/article/pii/S135044879900253X). *Radiat. Meas., 32, 57-73.*

5. Aitken, M.J. (1985). Thermoluminescence Dating. Academic Press, London, 267 pp.

6. Bell, W.T. (1979) Attenuation factors to absorbed dose in quartz inclusions for thermoluminescence dating. *Ancient TL, 8, 2–13.*

7. Prescott, J.R. and Hutton, J.T. (1994) Cosmic ray contribution to dose rates for luminescence and ESR dating: large depths and long-term time variations. *Radiat. Meas., 23, 497-500.*

8. Wintle, A.G. and Murray, A.S. (2006). A review of quartz optically stimulated luminescence characteristics and their relevance in single-aliquot regeneration dating protocol. *Radiat. Meas., 41, 369-391.*

9. Armitage, S.J., Duller, G.A.T. and Wintle, A.G. (2000). Quartz from Southern Africa: sensitivity changes as a result of thermal pre-treatment. *Radiat. Meas., 32, 571–577.*

10. Roberts, H.M. and Wintle, A.G. (2001) Equivalent dose determinations for polymineralic fine-grains using the SAR protocol: application to a Holocene sequence of the Chinese Loess Plateau. *Quaternary Sci. Rev., 20, 859–863*.
