# Supplementary material for: From Neandertals to modern humans: New data on the Uluzzian
Source: PLoS One. 2018 May 9;13(5):e0196786. doi: 10.1371/journal.pone.0196786 (PMC5942857; doi:10.1371/journal.pone.0196786)
Supplement: S3 File — (PDF) [file pone.0196786.s003.pdf]

Supporting Information

From Neandertals to modern humans: New data on the Uluzzian

Paola Villa*, Luca Pollarolo, Iacopo Conforti, Fabrizio Marra, Cristian Biagioni, Ilaria Degano, Jeannette J. Lucejko, Carlo Tozzi, Massimo Pennacchioni, Giovanni Zanchetta, Cristiano Nicosia, Marco Martini, Emanuela Sibilia, Laura Panzeri.

*villap@colorado.edu

S3 File. Lithic analysis

Only attributes or categories used in the analyses of la Fabbrica and Colle Rotondo are described.

Taphonomic attributes indicative of the state of preservation and formational history.

- State of preservation and dulling of the working edges, observations of two separate edges: fresh, slightly abraded [1]. Blocks and slabs can be rounded, indicating collecting from a river bank.
- Syn-or postdepositional chemical alteration: patinated, double patina, weathered.
- Burning: yes, no, partial.
- Breakage: complete, broken, almost complete

Attributes related to the mode of production

Blank:

- flake
- flake fragment
- pebble
- slab
- block
- core
- core fragment
- chunk

Cortex:

- presence or absence,
- type of cortex (pebble cortex, fresh, abraded, natural surface)
- percentage of cortex or natural surface (intervals of 0-4).

Platform type:

- cortical,
- plain,
- dihedral,
- facetted,
- ridge (i.e. dihedral or facetted but no individual preparation),

- punctiform,
- shattered (on ventral face),
- broken at knapping,
- removed.

Cores (all cores are by direct percussion, except bipolar cores).

Levallois cores. They are coded as in [1]**.** There are no Levallois cores in the Uluzzian layer of Grotta la Fabbrica. There is only one small Levallois core at Colle Rotondo which is polished by water action and with microfractures on the edges (Fig E in S1 File) and must be older than the rest of the Colle Rotondo assemblage which is fresh, if sometime patinated.

Non-Levallois cores: without any special preparation of the debitage surface or shaping of the core and a debitage surface subparallel to the great plane of the volume.

- With successive series of unidirectional parallel removals (Fig 9: A). Variation in core morphology depends on how the series of removals are organized, with a single platforms or two opposed platforms or orthogonal removals on a single debitage surface (Fig 10:A) or on adjacent surfaces (Fig 10:B).
- Bifacial cores, with alternating removals on opposite faces. Fig 9: B-C)
- Multidirectional core, with more than one debitage surface. Fig 11:A.
- Bladelet core with semi-rotating removals by direct percussion (Fig 12: A-B) and bladelet cores by the bipolar technique (Fig 12: C-D)
- Bipolar core (see Methods for description). Fig 11:B-C
- Core with non-conchoidal removals (also called flat cores). These are cores showing a particular variant of the bipolar technique (Fig A:B, this file); they are common in "Pontinian" assemblages [6] but extremely rare in the Uluzzian. None is present at La Fabbrica, only one at Colle Rotondo.
- Undetermined core (irregular or too few negatives or broken at knapping).

Types of flakes

Levallois flakes are coded in our Excel files as in [1: SI Fig Y]. However the number of Levallois flakes in the Mousterian assemblage is rather small (9 including tool blanks) so they are simply called Levallois. (Fig A: A, B, D, G, I in S1 File).

Flakes produced from non-Levallois, unprepared cores with a cortical or simply prepared platform. Remains of cortex appear on most blanks. Flakes from these cores have unidirectional, bidirectional, or orthogonal scars on the dorsal face**;** they may be without cortex, or have a cortical abrupt back or an oblique cortical facet on the side or on the end (Fig A: C in S1 File; Fig B in S1 File).

Some of these non-Levallois cores have produced flakes that might be classified as Levallois because they appear similar to Levallois flakes that do not preserve on their dorsal face negatives of removals controlling the convexities [1: 40]. There are 3 flakes of this kind (Fig C: A-C in S1 File), one from Colle Rotondo and two from La Fabbrica. This interpretation would, however, be incorrect. Note that the flake scars on the dorsal face to the left of each flake is very oblique, instead of parallel to the debitage surface of the other scars, as expected if these were Levallois flakes. In all three cases the platform is thin and plain. In other words these flakes have been produced by unidirectional or multidirectional cores.

Pseudo-Levallois points and *débordant* flakes. These are flakes that have removed a portion of the core edge, thus have a back formed by prior platform preparation removals. Dorsal scars can be convergent (points) or parallel or orthogonal with respect to the debitage axis (*débordant* flakes). We use the French term *débordant* because it has no exact equivalent in English. There is no core corresponding to the classic definition of discoid cores [2] and no systematic production of pseudo-Levallois points or *débordant* flakes. There are only two cases of pseudo-Levallois points at Colle Rotondo and three at La Fabbrica, illustrated in S1 File, Fig C: D-G, J in S1 File. We consider these flakes as products of multidirectional cores or cores with unidirectional scars on two adjacent debitage surfaces. A débordant flake with unidirectional scars of laminar flakes, orthogonal to the debitage axis is in Fig C: H in S1 File.

Flakes with centripetal scars but non-Levallois. Fig C: I in S1 File.

Ordinary flakes. These are the generic product of any kind of core, with no specific pattern. They may be cortical, partly cortical or non-cortical.

Bipolar flakes cores and flakes and scaled pieces are described in the Methods section.

Flat flakes are a special kind of flakes with non-conchoidal fracture [1]. Often confused with bipolar flakes these flakes are produced by striking a pebble resting on a soft anvil (e.g. wood or soft limestone) or resting the pebble on the ground and maintaining in a vertical position [3]. Flakes produced with this technique have a flat ventral face, a concave or crushed bulb of percussion but no measurable platform nor an opposing bulb or shattering of the distal end, which is typical of the bipolar technique. We call them flat flakes using a terminology first used at the Acheulian site of Terra Amata [4]. This technique is typical of "Pontinian" assemblages [5] but is very rare in the Uluzzian of Colle Rotondo and Grotta La Fabbrica (Fig. A).

Blades and bladelets made by direct percussion and by the bipolar technique. Blades and bladelets are classed following the common rule of length equal or greater than two times the width. Bladelets have width less than 12 mm, following the width boundary used by [6]. The width distribution at La Fabbrica and Colle Rotondo is only weakly bimodal (due to the small size of the samples) so the boundary is arbitrary. We set the maximum length of complete bladelets at 3 cm, based on size of scars on bladelet cores. Laminar (or elongated) flakes are like blades but do not have parallel sides (Fig. D: B in S1 File).

Small tool classification

We follow a simplified version of Bordes' typology [7] and classification is based on primary types (e.g. scrapers, end scrapers, denticulates, notches). Our categories do not cross-cut Bordes' types, they just lump them together. We do not use the term "atypical" and have eliminated edge shape from the definition of single, double, transverse and convergent scrapers. The retouch attributes (position, location, angle, extent; [8]) are coded in Excel files and used if deemed significant. Pieces with marginal retouch but continuous and regular on a fresh piece, hence very probably not due to postdepositional damage, are classified as 'Retouched piece". The term "bec" used in the main text is a commonly used French term indicating a thick awl or perforator done by alternate retouch.

"Utilized" pieces are artifacts showing signs of wear assumed to be due to human use i.e. discontinuous small scars along the edges, but with a form and intensity not suggestive of retouch. This term is in current use in the English literature [9] and corresponds to Bordes' éclats utilisés [10].

Scaled pieces which are tools by utilization, not by retouch, are not present in either Bordes' or Laplace's typology [11] but are defined in Upper Paleolithic industries [12]. See "Methods" for more detailed description.

Symbolic conventions used in artifact figures are as in [8].

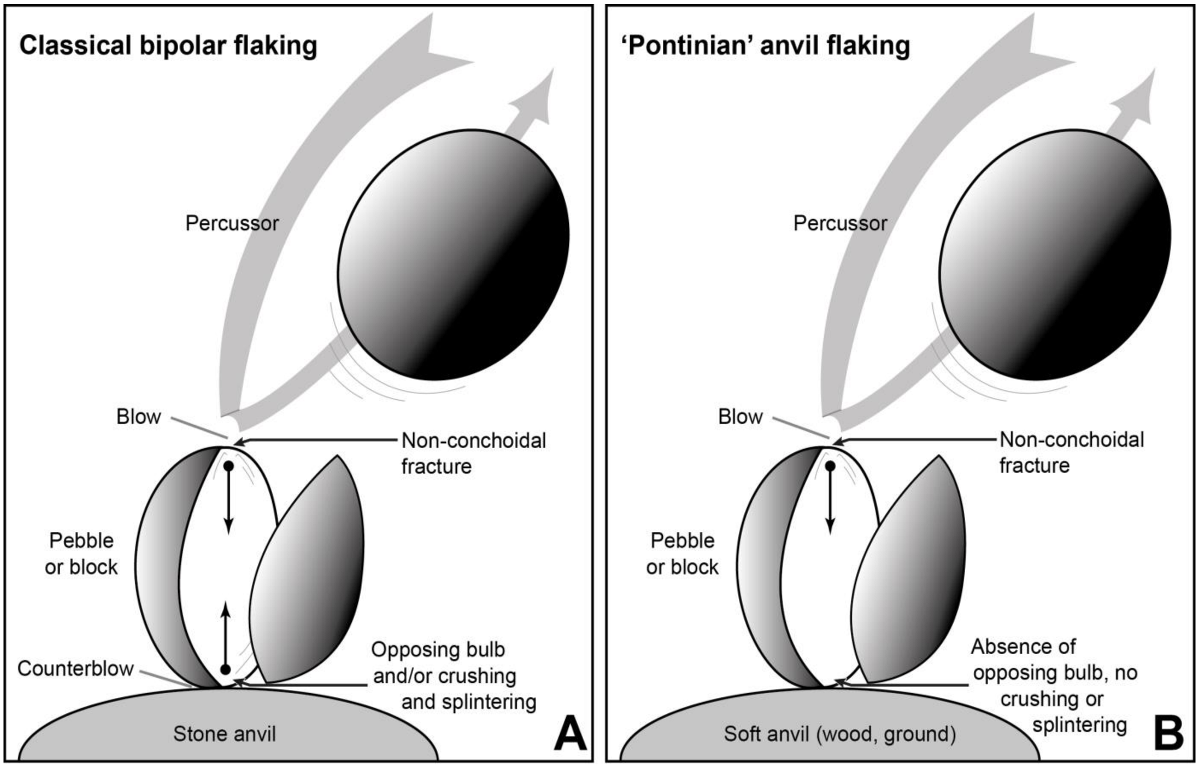

Fig. A. Differences between classical bipolar flaking and `Pontinian' anvil flaking. (A) With the typical bipolar flaking the development of the counterblow results in the formation of an opposing bulb and/or crushing and splintering. (B) With a soft anvil the counterblow doesn't generate any feature on flakes or cores. After [5].

References

1. Villa P, Soriano S, Grün R, Marra F, Nomade S, Pereira A et al. The Acheulian and Early Middle Paleolithic in Latium (Italy): Stability and Innovation. PLoS ONE 11(8): e0160516.

2. Boëda E. Le débitage discoïde et le débitage Levallois récurrent centripète. Bull Soc Préhist Française 1993; 90: 392-404.

3.Faivre JPh, Geneste JM, Turq A.La fracturation en split, une technique de production dans l'industrie lithique des Tares (Sourzac, Dordogne). Paléo 2009-2010; 133–142.

4. Villa, P. Terra Amata and the Middle Pleistocene archaeological record of Southern France. Berkeley and Los Angeles, University of California Press; 1983.

5. Soriano S, Villa P. Early Levallois and the beginning of the Middle Paleolithic in Central Italy. PLoS ONE 12(10): e0186082. https://doi.org/10.1371/journal.pone.0186082.

6. Tixier J. Typologie de l'Epipaléolithique du Maghreb. Paris: Arts et métiers graphiques (Étampes, Impr. SRIP); 1963.

7. Villa P, Delagnes A, Wadley L. A late Middle Stone Age artifact assemblage from Sibudu (KwaZulu-Natal). Comparisons with the European Middle Paleolithic. J Archaeol Sci 2005; 32, 399–422.

8. Inizan M-L, Reduron-Ballinger M, Roche H, Tixier J. Technology and Terminology of Knapped Stone. Nanterre: Cercle de Recherches et d'Etudes Préhistoriques; 1999.

9. Isaac Gl L. Olorgesailie. University of Chicago Press; 1977.

10. Bordes F. Typologie du Paléolithique ancient et moyen. Bordeaux: Delmas; 1961.

11. Laplace G. Essai de typologie systématique. Ferrara, Annali Università di Ferrara, n.s. sezione XV, suppl II: 1964.

12. Demars PY, Laurent P. Types d'outils lithiques du Paléolithique supérieur en Europe. Paris: Editions CNRS; 1989.
