# Supplementary material for: From Neandertals to modern humans: New data on the Uluzzian
Source: PLoS One. 2018 May 9;13(5):e0196786. doi: 10.1371/journal.pone.0196786 (PMC5942857; doi:10.1371/journal.pone.0196786)
Supplement: S4 File — (DOCX) [file pone.0196786.s004.docx]

**Supporting Information**

**From Neandertals to modern humans:**

**New data on the Uluzzian**

Paola Villa*, Luca Pollarolo, Jacopo Conforti, Fabrizio Marra, Cristian Biagioni, Ilaria Degano, Jeannette J. Lucejko, Carlo Tozzi, Massimo Pennacchioni, Giovanni Zanchetta, Cristiano Nicosia, Marco Martini, Emanuela Sibilia, Laura Panzeri.

*[villap@colorado.edu](mailto:villap@colorado.edu)

S4 File. X-ray diffraction, chemical analysis

This Word file includes:

X-ray diffraction of La Fabbrica ochre powder

Chemical analysis of hafting adhesives

**X-ray diffraction of La Fabbrica pigment (CB)**

Samples of ochraceous material collected in the La Fabbrica site, similar to those occurring as thin coatings of the bone awl and the stone flakes, were investigated through X-ray powder diffraction using a Bruker D2 Phaser diffractometer (30 kV, 10 mA) operating in Bragg-Brentano geometry (θ-θ geometry) and equipped with a one-dimensional Lynxeye detector. Ni-filtered Cu*K*α radiation was used. The X-ray powder diffraction pattern (Figure A) was collected in the scan range 4-65° in 2θ, with scan step of 0.02° (in 2θ) and counting time 0.1 s/step. Samples are mainly composed by hematite and quartz, in agreement with EDS chemical data. Very weak reflections at ~ 4.2 Å and 3.00 Å, corresponding to the strongest reflections of goethite and calcite, respectively, suggest the occurrence of trace amounts of these minerals.

#
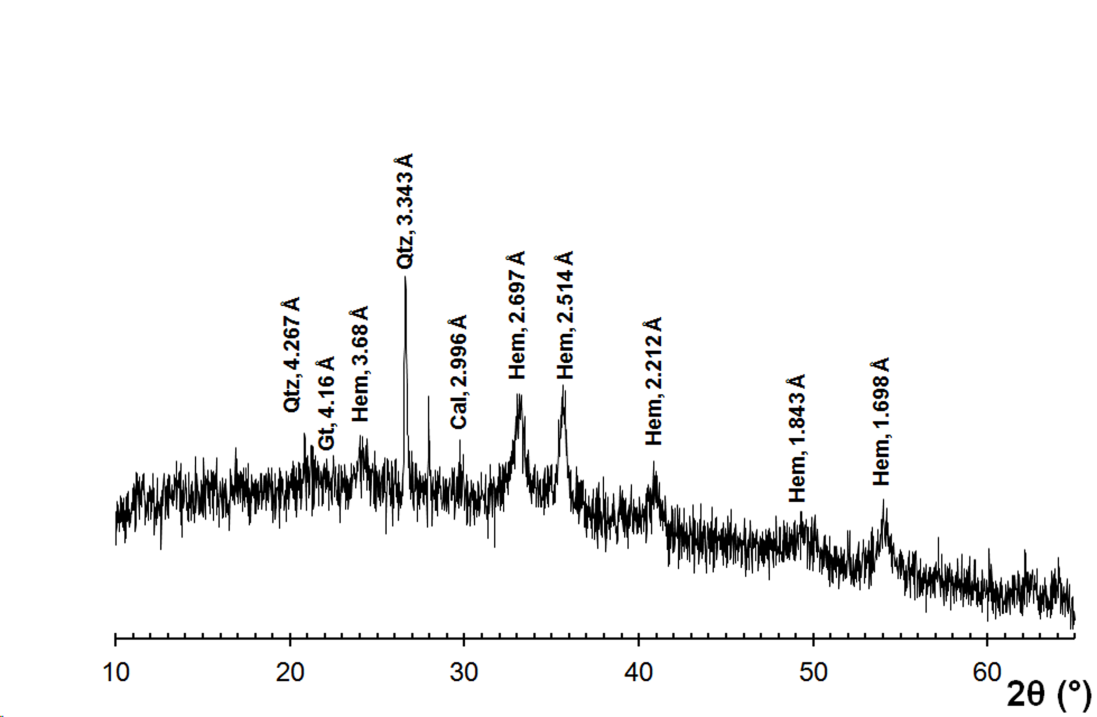


Figure A. X-ray powder diffraction pattern of the ochre sample from La Fabbrica. Labels: Cal = calcite; Hem = hematite; Gt = goethite; Qtz = quartz. The values of the d_hkl_ (in Å) are shown.

**Chemical analysis of hafting adhesives by GC/MS (ID, JJL)**

# Sample treatment

Samples are subjected to alkaline hydrolysis (saponification) in 1mL of KOH solution (KOH_CH3OH_/KOH_H2O_ 2:3; 10% KOH w/w), 60 °C, 3h. After hydrolysis, the neutral organic compounds have been extracted with n-hexane (500 µL 3 times; the extract contains the neutral fraction). After acidification of the residual to pH=2, the acidic organic components are extracted with diethylether (500 µL 3 times; the extract is the acidic fraction). The two fractions are combined, dried and derivatised for GC/MS analysis, by adding 5 µL of internal standard (tridecanoic acid in isooctane) and 20 µL of derivatising agent (*N,O*-trimethyl-silyl-trifluoroacetamide containing 1% of trimethylchlorosilane). The reaction takes place at 60 °C for 30 min, in 150 µL isooctane. 5 µL of the injection internal standard (hexadecane in isooctane) are added to the solution before injection in the GC/MS (2 µL).

# Apparatus

The GC/MS instrumentation consists in a 6890N Network GC System (Agilent Technologies, Palo Alto, CA, USA) equipped with a PTV injector and coupled to a 5973 MS detector with quadrupole analyser. The pyrolyser coupled to the GC/MS was a 5150 CDS Pyroprobe 5000 Series pyrolyser with a platinum filament.

MS parameters: electron impact ionisation (EI, 70 eV) in positive mode; ion source temperature 230 °C; scan range 50-700 m/z; interface temperature 280 °C.

GC separation was performed on a HP-5MS column (J&W Scientific, Agilent Technologies, stationary phase 5% phenil–95% methylpolysiloxane, 30m lenght, 0.25 mm i.d., 0.25 μm film thickness) connected to a deactivated fused silica precolumn (J&W Scientific, Agilent Technologies, 2 m length, 0.32 mm i.d.). The PTV injector was used in splitless mode at 300 °C and the chromatographic oven was programmed as follows 80° C, for 2 min isothermal, 10 °C/min up to 200 °C, 4 min isothermal, 6 °C/min up to 280°C, 40 minutes isothermal; constant He flow 1.2 mL/min, injector temperature 280 °C.

**
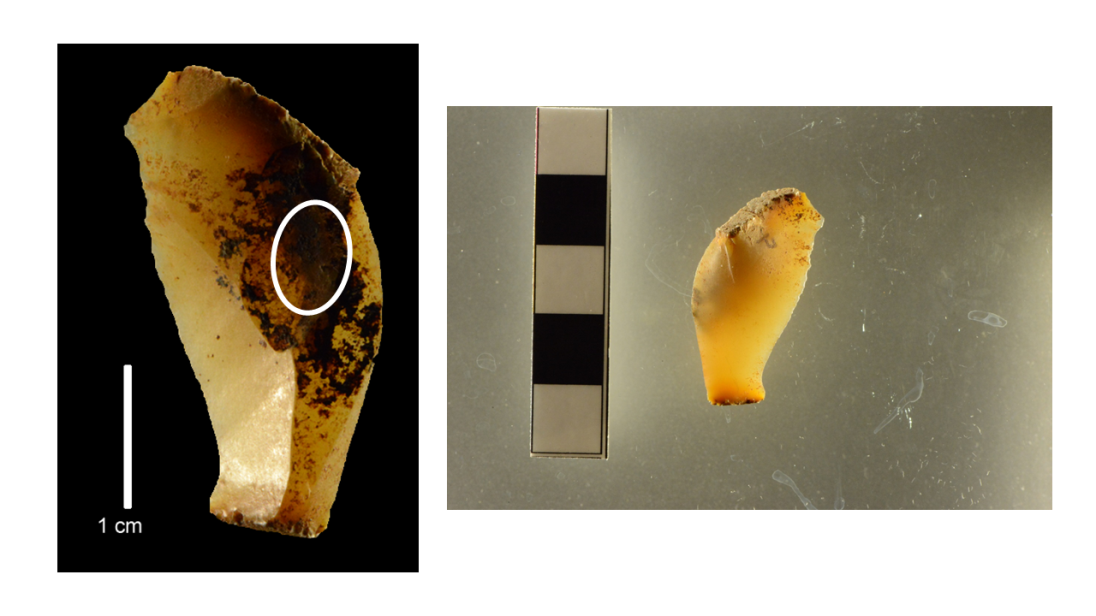
**

Figure B. Sample 1. Unretouched flake from Colle Rotondo. The white circle indicates the location of sampling.


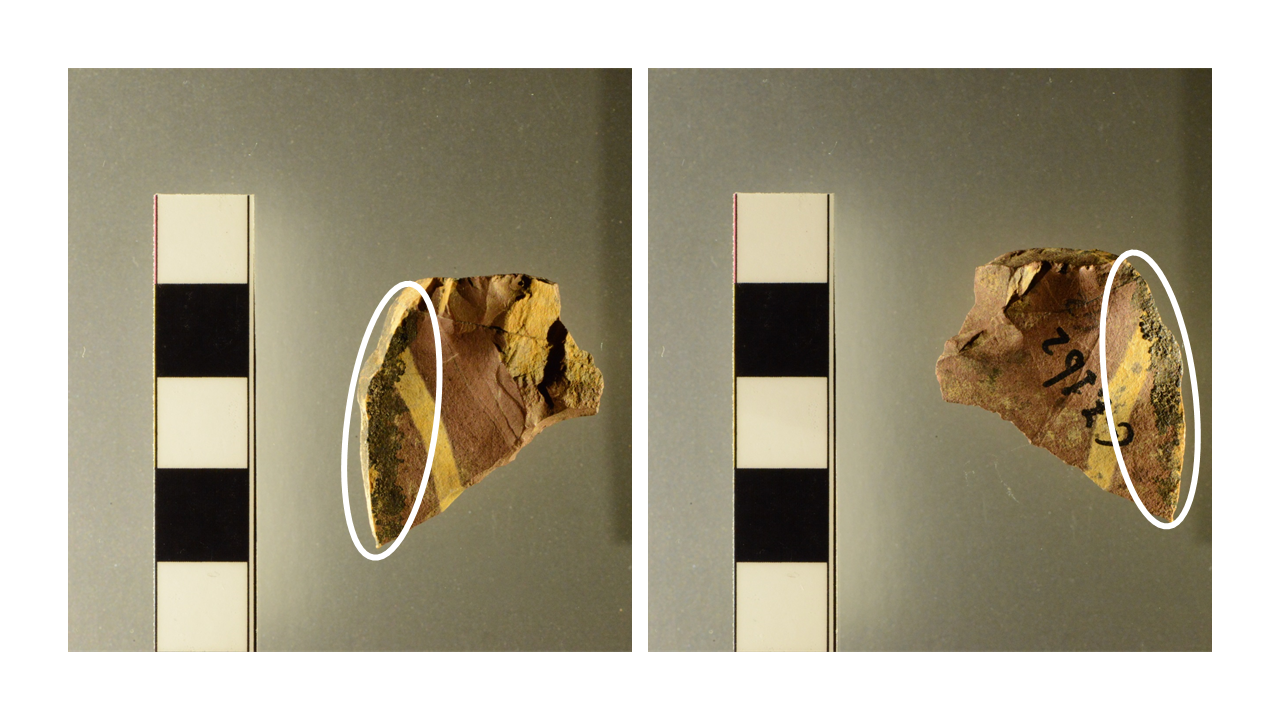


Figure C. Sample 2. Grotta La Fabbrica, side scraper from Mousterian layer 1b. The white ellipse indicates the location of sampling.


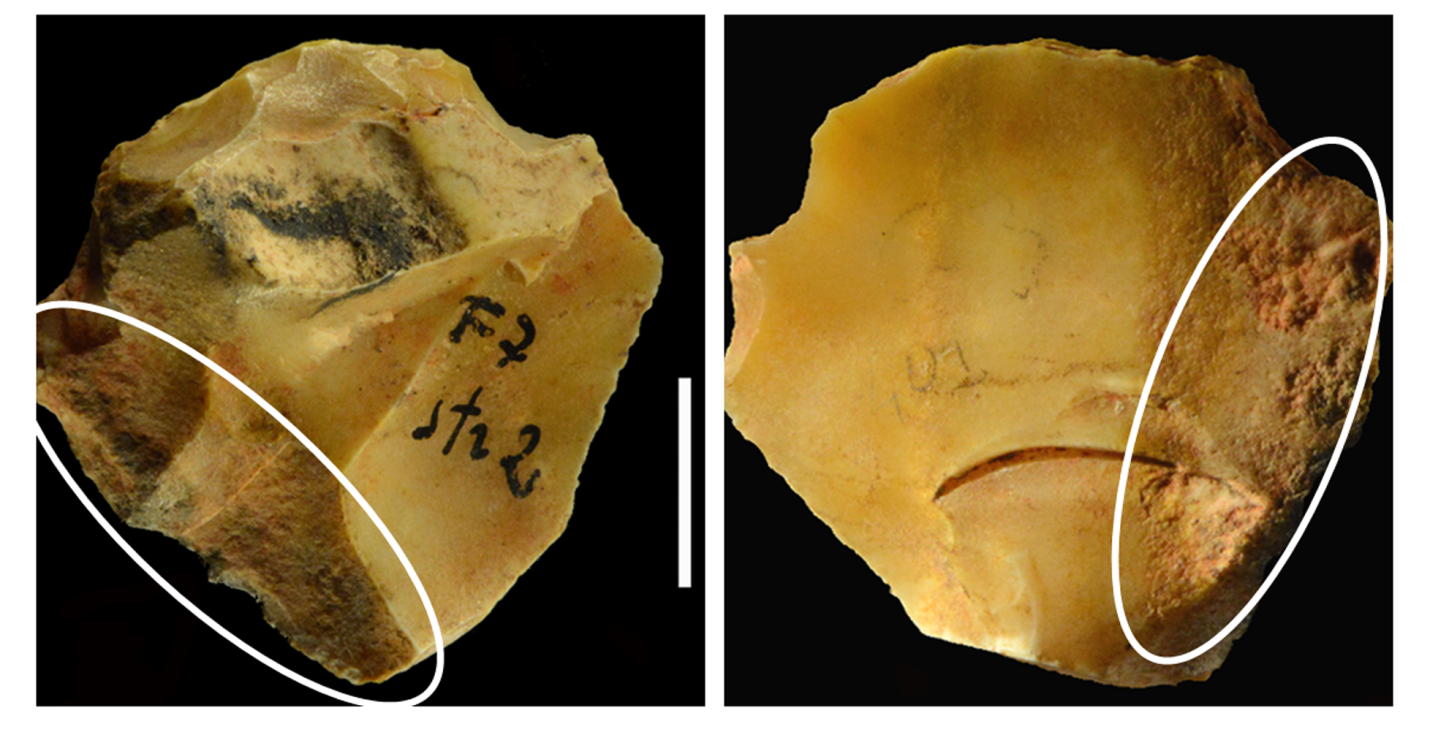


Figure D. Sample 3. Grotta La Fabbrica, End scraper from Uluzzian layer 2. The white ellipse indicates the location of sampling.

# Results

The chromatograms are reported in figures E-F. Table A reports the peak assignments for all the samples. Gas chromatography/mass spectrometry (GC/MS) was applied to the characterization of the amorphous materials on three samples (two from Fabbrica and one from Colle Rotondo sites). The sediment corresponding to the sampled pieces was also analyzed to rule out external contamination.

The three samples had clearly visible residues on the backed edge. Microsamples (less than 0.1 mg) were collected from the specimens and submitted to a combined analytical procedure for the identification of lipids, waxes, and resinous materials^[[1]](#endnote-1)^.

With regard to the sample 1. collected from Colle Rotondo, the chromatographic profile of the sample’s extract and the sediment’s perfectly matched (Figure F). Thus, no hafting material was identified.

With regard to the two samples from Fabbrica, the profile of sample 2 (chromatogram (a) in Figure E) the Mousterian side scraper did not differ from that of the sediment (chromatogram (b) in Figure E) except for the relative abundances of the compounds. Although more peaks were identified in the side scraper than in the sediment, their qualitative profile is the same. Thus, no conclusion can be drawn on the presence of hafting material.

With regard to sample 3, the Uluzzian end scraper , the chromatogram (Figure E: c) showed the presence of a relevant amount of diterpenes (peaks #22, #23 and #28), indicating the use of a conifer resin. The main components of the diterpenic fraction are dehydroabietic and 7-oxo-dehydroabietic acids, indicating a resin from a plant belonging to the conifer group such as *Pinaceae*. Moreover, the profile of the lipid components differs from that shown in the sediment (Figure E:d) and thus suggests the co-presence of plant and animal fats, due to the occurrence of fatty acids with even and odd carbon number, ranging from C12 (dodecanoic acid) to C25 (pentacosanoic acid) [1].


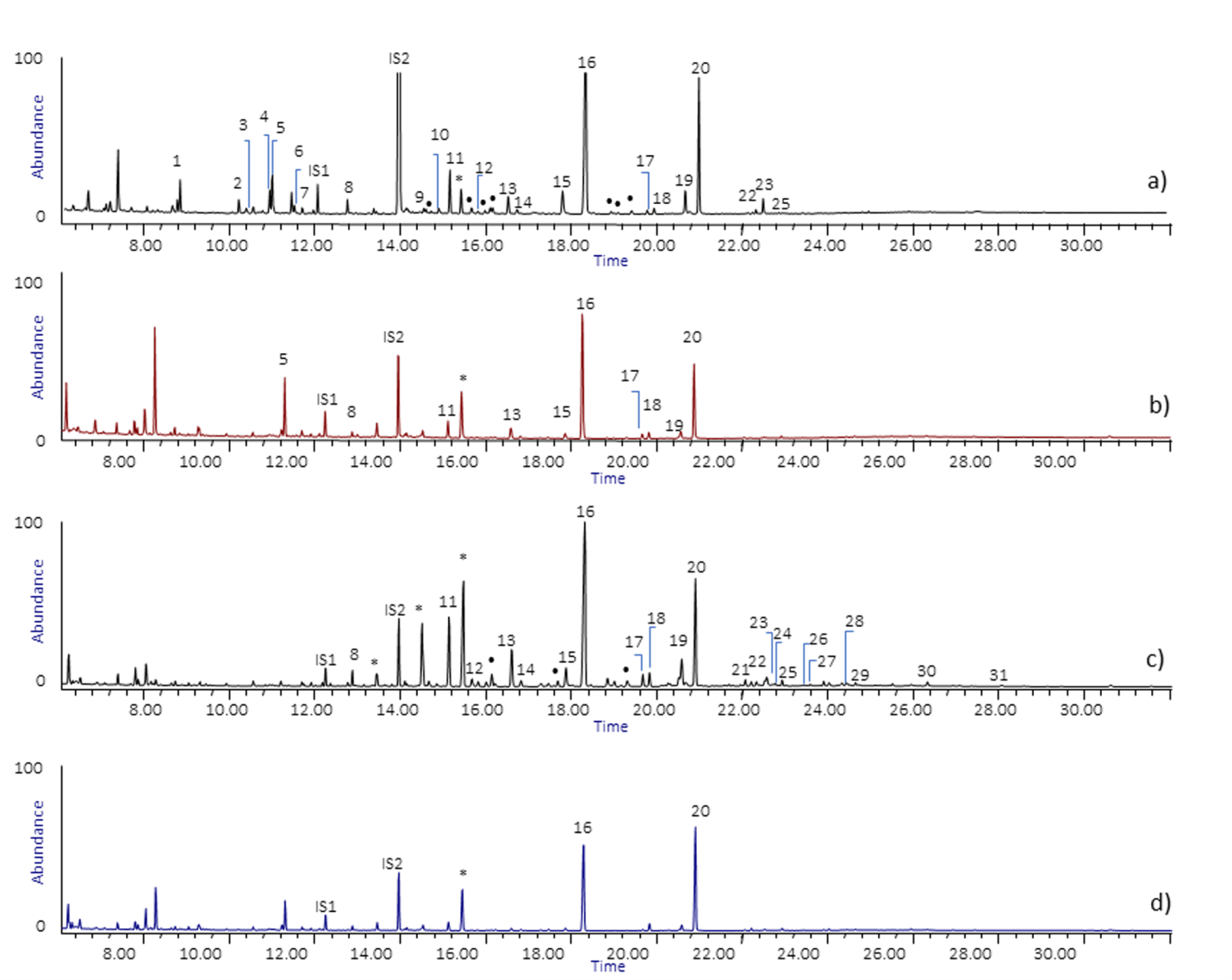


Figure E: Gas chromatograms of the combined acidic and neutral fractions of the samples from La Fabbrica: a) Mousterian side scraper; b) Mousterian level sediment; c) Uluzzian end scraper; d) Uluzzian level sediment.

Figure F: Gas chromatograms of the combined acidic and neutral fractions of the samples from Colle Rotondo: e) unretouched flake; f) sediment.

Table A. Peak assignments for chromatograms

| # | **Compound** |
| --- | --- |
| 1 | nonanoic acid |
| 2 | decanoic acid |
| 3 | acetic acid |
| 4 | 2,4-ditertbutylphenol |
| 5 | ditertbutyl-p-cresol |
| 6 | undecanoic acid |
| 7 | dodecanol |
| IS1 | hexadecane (is) |
| 8 | dodecanoic acid |
| IS2 | tridecanoic acid (is) |
| 9 | nonandioic acid (azelaic acid) |
| 10 | myristoleic acid |
| 11 | tetradecanoic acid (myristic acid) |
| 12 | decandioic acid (sebacic acid) |
| 13 | pentadecanoic acid (also branched) |
| 14 | hexadecanol |
| 15 | hexadecenoic acid (palmitelaidic acid) |
| 16 | hexadecanoic acid |
| 17 | eptadecanoic acid (also branched) |
| 18 | octadecanol |
| 19 | octadecenoic acid |
| 20 | octadecanoic acid |
| 21 | nonadecanol |
| 22 | compounds with abietadienic structure |
| 23 | dehydroabietic acid (dha) |
| 24 | eicosanol |
| 25 | eicosanoic acid |
| 26 | heneicosanoic acid |
| 27 | docosanol |
| 28 | 7-oxo-dehydroabietic acid |
| 29 | docosanoic acid |
| 30 | tetracosanoic acid |
| 31 | pentacosanoic acid |

**References**

1. Ribechini E, Modugno F. GC/MS in the characterisation of resinous materials. In: Colombini MP, Modugno F, editors. Organic Mass Spectrometry in Art and Archaeology. John Wiley and Sons; 2009: 158–16.

1. [↑](#endnote-ref-1)
