# Supplementary material for: From Neandertals to modern humans: New data on the Uluzzian
Source: PLoS One. 2018 May 9;13(5):e0196786. doi: 10.1371/journal.pone.0196786 (PMC5942857; doi:10.1371/journal.pone.0196786)
Supplement: S5 File — (PDF) [file pone.0196786.s005.pdf]

Supporting Information

From Neandertals to modern humans: New data on the Uluzzian

Paola Villa*, Luca Pollarolo, Iacopo Conforti, Fabrizio Marra, Cristian Biagioni, Ilaria Degano, Jeannette J. Lucejko, Carlo Tozzi, Massimo Pennacchioni, Giovanni Zanchetta, Cristiano Nicosia, Marco Martini, Emanuela Sibilia, Laura Panzeri.

*villap@colorado.edu

S5 File. Permissions from copyright holders

This Word file includes: Permission from Carlo Cavanna to use and modify Figure 1C
Permission from Regione Lazio to use and modify Figure 2A

permission

Carlo Cavanna <carlocavanna1@gmail.com>

Tue 3/6/2018 7:56 AM

To:Paola Villa <paola.villa@colorado.edu>;

Cc:conforti.jacopo@gmail.com <conforti.jacopo@gmail.com>;

Dear Dr. Conforti,

You have my permission to use in modified form the figure (p. 31) published in **Dini M., Mezzasalma S., Tozzi C.** - *Il Paleolitico medio di Grotta La Fabbrica (Parco dell'Uccellina - Grosseto) pp. 31-52.* In: a cura di Cavanna C. - La preistoria nelle grotte del Parco Naturale della Maremma - Atti del Museo di Storia Naturale della Maremma, Supplemento al n° 22. Your proposed changes to our figure certainly modify the original enough so as to fall within the parameters of Fair Use. I would appreciate if you would include attribution that your new figure is modified from our publication.

Carlo Cavanna

Mail priva di virus. www.avg.com

****

MASSIMO PENNACCHIONI <massimo.pennacchioni@persociv.difesa.it>
Thu 3/15, 1:33 AMPaola Villa

Dear Paola

I spoke on the telephone with Sergio Varcasia at "Direzione Regionale Territorio, Urbanistica
e Mobilità" (svarcasia@regione.Lazio.it) asking for permission to publish a portion of the Latium map at 1:10000.

He said it is **open data** and it is enough to cite the provenience.

Best regard.

dott. Massimo Pennacchioni
Italian Air Force
Centro Informazioni Geotopografiche Aeronautiche
Servizio Elaborazioni Geotopografiche - Sezione Cartografia
Nucleo Basi Geografiche

mail: massimo.pennacchioni@persociv.difesa.it
